# Supplementary material for: The PARTNER trial of neoadjuvant olaparib with chemotherapy in triple-negative breast cancer
Source: Nature. 2024 Apr 8;629(8014):1142–8. doi: 10.1038/s41586-024-07384-2 (PMC11136660; doi:10.1038/s41586-024-07384-2)
Supplement: Supplementary file 1 — Summary from protocol; and PARTNER trial consortium members. [file 41586_2024_7384_MOESM1_ESM.pdf]

nature portfolio

https://doi.org/10.1038/s41586-024-07384-2

Supplementary information

The PARTNER trial of neoadjuvant olaparib with chemotherapy in triple-negative breast cancer

In the format provided by the authors and unedited

The PARTNER trial of neoadjuvant olaparib with chemotherapy in triple-negative breast cancer

Supplementary Information

Summary from protocol

Randomised, phase II/III, 3 stage trial to evaluate the safety and efficacy of the addition of olaparib to platinum-based neoadjuvant chemotherapy in breast cancer patients with TNBC

1. Details of study stages

This was an open label randomised 3-stage phase II/III trial. Eligible patients with early breast cancer were assessed for basal status using the following immune-histological assessments: CK5/6 and/or EGFR and if appropriate Androgen receptor.

In Stage 1 and 2, patients were randomised with a ratio 1:1:1 to either control or one of the two research arms. At the end of Stage 2, one of the two research arms was dropped. The recruitment continued into all three arms while waiting for the results of analysis of Stage 1 and Stage 2. One research group (olaparib on day 3 to day 14) was selected following the recommendation of the independent data safety monitoring committee (IDSMC), based on pre-specified criteria. Patients were then randomised with a ratio 1:1 to either control arm or the selected research arm (olaparib on day 3 to day 14). It was planned to recruit a minimum of 780 patients in total. The recruitment of patients with TNBC gBRCAwt and patients with gBRCA mutated (gBRCAm) breast cancer were completed independently. It was planned to recruit a minimum of 478 TNBC non-gBRCAwt and 188 gBRCAm patients.

Stage 1 (n=75, 25 patients in each of the two research arms) – Safety.

The primary outcome measure in stage 1 was safety within the research arms. Although all toxicities were considered, particular considerations were given to CTCAE grade 3 and 4 toxicities that appeared to be associated with olaparib especially grade 3 and 4 febrile neutropenia and thrombocytopenia, or events that require dose reduction or discontinuation of olaparib. If there was no evidence that the grade 3 and 4 adverse events as specified above exceeded 35% (i.e. more than 14 out of 25 patients for each of the research arm, or more than 24 out of 50 patients combining both research arms), the trial was continued to Stage 2. Otherwise, the research regimens would be amended. Safety was also compared between the control and experimental arms in an exploratory manner.

Stage 2 (n=159, 53 in each of the two research arms) – Selection of a research arm

Pathological complete response rate (pCR) rate post-surgery was taken together with completion rate of olaparib protocol treatment. It was a "pick-the winner" design with 53 patients in each research arm. This had a 90% power, 5% one-sided significance level to test null hypothesis of pCR ≤ 35% versus an alternative hypothesis of pCR ≥ 55% in each of the research arms.

Recruitment continued while waiting for the results of the analysis based on the 106 patients randomised in both research arms. Assuming that it would take 9 months (6 month to surgery + 3 months data cleaning and analysis) from the date when 106 patients were randomised in both research arms to the date when decision on Stage 2 was made, there were approximately 90 patients randomised in the trial (60 between the two research arms). All 166 patients randomised to the two research arms until end of Stage 2 are being followed-up, and thus provide information between the two research arms; a difference of pCR around 18% between the two research arms could be detected with a 10% significance level (two-sided) and 80% power.

Stage 3 (n=666 between control and the research arm selected in Stage 2) - Efficacy

The primary outcome measure was pCR. Based on the evidence from GeparSixto and CALGB 40603 [1–3] the anticipated pCR was ~45-55% for TNBC gBRCAwt patients and ~50-60% for gBRCA mutated patients, receiving platinum-based chemotherapy. The trial was powered to detect an absolute improvement of 15% for TNBC gBRCAwt and 20% for gBRCAm patients by adding olaparib to chemotherapy.

References:

1. von Minckwitz G, Schneeweiss A, Loibl S, *et al.* Neoadjuvant carboplatin in patients with triple-negative and HER2-positive early breast cancer (GeparSixto; GBG 66): a randomised phase 2 trial. *Lancet Oncol* 2014; **15**:747–756.

2. Sikov WM, Berry DA, Perou CM, *et al.* Impact of the addition of carboplatin and/or bevacizumab to neoadjuvant once-per-week paclitaxel followed by dose-dense doxorubicin and cyclophosphamide on pathologic complete response rates in stage II to III triple-negative breast cancer: CALGB 40603 (A. *J Clin Oncol* 2015; **33**:13–21.

3. Gunter Von Minckwitz, Eric Hahnen, Peter A. Fasching, Jan Hauke, Andreas Schneeweiss, Christoph Salat, Mahdi Rezai, Jens U. Blohmer, Dirk Michael Zahm, Christian Jackisch, Bernd Gerber, Peter Klare, Sherko Kummel, Holger Eidtmann, Stefan Paepke, Valentina and G and A-BSG. Pathological complete response (pCR) rates after carboplatin-containing neoadjuvant chemotherapy in patients with germline BRCA (gBRCA) mutation and triple-negative breast cancer (TNBC): Results from GeparSixto. *J Clin Oncol* 2014; **32:15_supp**:1005–1005.

PARTNER Trial Consortium Members

Project team (in alphabetical order, by surname):

Current: Jean Abraham (Chief Investigator); Anita Chhabra; Elizabeth Cromwell; Alimu Dayimu; Erdem Demir; Nikolaos Demiris; Helena Earl; Steven Frost; Alexander Fulton; Louise Grybowicz; Debra Hall; Caron Harvey; Nicola Johnson; Karolina Lazarowicz; Rebecca Lucey; Jessica Martin; Simon Morrison; Abirami Nandakopan; Karen Pinilla; Camila Pontes; Elena Provenzano; Nicola Robinson; Philip Schouten; Jeschito Serrano; Marc Tischkowitz; Anne-Laure Vallier; Nicky Watson; [Addenbrooke's Hospital].

Past: Angels Kateb Castellnou; Vasiliki Chronopoulou; Edmund Chui; Luke Hughes Davies; Lynsey Drewett; Richard Hardy; Sian Loi; Emma McMurtry; Oluwatofunmi Okude; Wendi Qian; Anne Roberts; Cherry May Sanchez; Stanly Thomas; Melanie Weiss; Joanna Worley; Gemma Young;

Trial Management Group (current) includes Project team members and the following site PIs (in alphabetical order, by surname):

Anne Armstrong (Co-Chair); The Christie NHS Foundation Trust. Ellen Copson (Co-Chair); Southampton General Hospital. Karen McAdam; Peterborough City Hospital. Jacqueline Newby; Royal Free Hospital.

Surgical Input: Parto Forouhi, Addenbrooke's Hospital

Site Hospitals and Principal Investigators (in alphabetical order, by Hospital Name)

Basingstoke and North Hampshire Hospital and Royal Hampshire Hospital, Winchester; Sanjay Raj. Bedford Hospital; Zaheer Abbas; Shahzeena Aslam; Sarah Smith. Cambridge University Hospitals NHS Trust; Jean Abraham. Churchill Hospital; Nicola Levitt. Colchester Hospital; Mukesh Mukesh. Hinchingbrooke Hospital; Cheryl Palmer. Ipswich Hospital; Elizabeth Sherwin; Ramachandran Venkitaraman. Mount Vernon Cancer Centre; Stephanie Sutherland. Nottingham City Hospital; Steve Chan. Peterborough City Hospital; Sarah Ayers; Karen McAdam. Pinderfields Hospital; David Glassman; Sreedevi Kumar; Rasheid Mekki; Jay Naik. Poole Hospital; Amit Chakrabarti. Queen's Hospital; Emma Staples. Queen's Hospital Burton; Mojca Persic. Royal Bournemouth Hospital; Maria Esther Una Cidon; Tamas Hickish. Royal Free Hospital; Jacqueline Newby. Russells Hall Hospital; Rozenn Allerton; Ravi Dandamudi; Devashish Tripathi. Singleton Hospital; Gianfilippo Bertelli; David Mark Davies. Southampton General Hospital; Ellen Copson. The Christie NHS Foundation Trust; Anne Armstrong. University College Hospital; Rebecca Roylance. University Hospital Crosshouse; Lucy Scott. University Hospitals Bristol and Weston NHS Foundation Trust; Jeremy Braybrooke. Velindre Cancer Centre; Jacinta Abraham; Annabel Borley. West Suffolk Hospital; Margaret Moody. Worcestershire Royal Hospital, Kidderminster Hospital and Treatment Centre; Alexandra Redditch Hospital, Redditch; Mark Churn; Chris Price.
