# Supplementary material for: The PARTNER trial of neoadjuvant olaparib with chemotherapy in triple-negative breast cancer
Source: Nature. 2024 Apr 8;629(8014):1142–8. doi: 10.1038/s41586-024-07384-2 (PMC11136660; doi:10.1038/s41586-024-07384-2)
Supplement: Supplementary file 2 — Reporting Summary [file 41586_2024_7384_MOESM2_ESM.pdf]

nature portfolio

Corresponding author(s): Jean E Abraham

Last updated by author(s): Mar 15, 2024

Reporting Summary

Nature Portfolio wishes to improve the reproducibility of the work that we publish. This form provides structure for consistency and transparency in reporting. For further information on Nature Portfolio policies, see our Editorial Policies and the Editorial Policy Checklist.

Statistics

For all statistical analyses, confirm that the following items are present in the figure legend, table legend, main text, or Methods section.

n/a	Confirmed	
☐	☒	The exact sample size (n) for each experimental group/condition, given as a discrete number and unit of measurement
☒	☐	A statement on whether measurements were taken from distinct samples or whether the same sample was measured repeatedly
☐	☒	The statistical test(s) used AND whether they are one- or two-sided<br>*Only common tests should be described solely by name; describe more complex techniques in the Methods section.*
☐	☒	A description of all covariates tested
☐	☒	A description of any assumptions or corrections, such as tests of normality and adjustment for multiple comparisons
☐	☒	A full description of the statistical parameters including central tendency (e.g. means) or other basic estimates (e.g. regression coefficient) AND variation (e.g. standard deviation) or associated estimates of uncertainty (e.g. confidence intervals)
☐	☒	For null hypothesis testing, the test statistic (e.g. F, t, r) with confidence intervals, effect sizes, degrees of freedom and P value noted<br>*Give P values as exact values whenever suitable.*
☒	☐	For Bayesian analysis, information on the choice of priors and Markov chain Monte Carlo settings
☐	☒	For hierarchical and complex designs, identification of the appropriate level for tests and full reporting of outcomes
☒	☐	Estimates of effect sizes (e.g. Cohen's d, Pearson's r), indicating how they were calculated

Our web collection on statistics for biologists contains articles on many of the points above.

Software and code

Policy information about availability of computer code

Data collection	Data were collected with the MACRO database.
Data analysis	All statistical analyses were performed with R v4.1.0 (also mentioned in the manuscript within the methods section)

For manuscripts utilizing custom algorithms or software that are central to the research but not yet described in published literature, software must be made available to editors and reviewers. We strongly encourage code deposition in a community repository (e.g. GitHub). See the Nature Portfolio guidelines for submitting code & software for further information.

Data

Policy information about availability of data

All manuscripts must include a data availability statement. This statement should provide the following information, where applicable:

- Accession codes, unique identifiers, or web links for publicly available datasets
- A description of any restrictions on data availability
- For clinical datasets or third party data, please ensure that the statement adheres to our policy

Data collected within the PARTNER study will be made available to researchers whose full proposal for their use of the data has been approved by the PARTNER Trial Management Group and whose research includes a clear and comprehensive research plan with statistical considerations adequately completed. The data required for the approved, specified purposes and the trial protocol will be provided, after completion of a data sharing agreement. Data sharing agreements will be set up

by the Trial Steering and Management Groups and will include clear instructions on publication, reporting and usage policy. A minimum dataset of anonymised data will be made available after full publication of the trial and related work. Please address requests for data to ja344@cam.ac.uk

Research involving human participants, their data, or biological material

Policy information about studies with human participants or human data. See also policy information about sex, gender (identity/presentation), and sexual orientation and race, ethnicity and racism.

Reporting on sex and gender	The study focused on breast cancer patients. Although gender or sex identifying information was not collected, it is likely that the majority of participants in this study were assigned female at birth.
Reporting on race, ethnicity, or other socially relevant groupings	The study included all participants who met eligibility criteria for the study with no data collected on race, ethnicity, or other socially relevant groupings.
Population characteristics	Patients aged between 16 and 70 years with histologically confirmed stage T1-4, N0-2 (tumour or axillary lymph node diameter ≥ 10mm) invasive breast cancer, confirmed ER-negative and HER2-negative, and Eastern Cooperative Oncology Group performance status (ECOG PS) 0-1 were eligible. Detailed eligibility criteria are provided in the methods section.
Recruitment	Participants were assessed after consent at 29 UK centres. Eligible participants were randomised to either treatment group(s) or the control. All patients across all sites were assessed for eligibility criteria during their standard clinical evaluation and multidisciplinary team meeting. The trial was offered when it was considered clinically appropriate. There were no self-selection or site-based biases involved.
Ethics oversight	The PARTNER trial protocol (NCT03150576) was approved by North West - Haydock Research Ethics Committee (ref: 15/NW/0926)

Note that full information on the approval of the study protocol must also be provided in the manuscript.

Field-specific reporting

Please select the one below that is the best fit for your research. If you are not sure, read the appropriate sections before making your selection.

☒ Life sciences ☐ Behavioural & social sciences ☐ Ecological, evolutionary & environmental sciences

For a reference copy of the document with all sections, see nature.com/documents/nr-reporting-summary-flat.pdf

Life sciences study design

All studies must disclose on these points even when the disclosure is negative.

Sample size	In this TNBC (gBRCAwt) cohort, a total of 454 patients were needed to attest with 90% power and 5% significance level the null hypothesis of no difference in pCR rate between the two groups versus the alternative of 50% in control group and 65% in research group. Considering a non-compliance of 5%, it was planned to recruit a total of 478 TNBC (gBRCAwt) patients between the control and the selected research group.
Data exclusions	The main analysis was conducted based on the modified intention-to-treat (mITT) principle, which included all randomised, eligible patients excluding only those who did not start treatment. The safety analyses included patients who had at least one dose of trial treatment.
Replication	The main analyses were performed by another independent statistician and checked against the results of the trial statistician. Two pathologists independently reviewed the slides. The main analysis were performed by another independent statistician and checked against the results of the trial statistician at the final stage of the study. Two pathologist independently reviewed the slides simultaneously. All replications were successful.
Randomization	The trial was open label and eligible patients were randomly assigned to either the control group (chemotherapy: paclitaxel 80mg/m2 on day 1, 8 & 15 and carboplatin AUC5 on day 1), or one of the two research groups (chemotherapy with olaparib 150mg twice daily on day -2 to day 10 OR day 3 to day 14) using a minimisation method in a 1:1:1 ratio in Stage 1 and Stage 2 of the trial with a web-based central randomisation system. In Stage 3 (reported here), patients were randomly assigned with a 1:1 ratio to either control or research arm (olaparib 150mg bd on days 3 to day 14).
Blinding	This is an open label study. The pathologists were blinded to the treatment arm.

Reporting for specific materials, systems and methods

We require information from authors about some types of materials, experimental systems and methods used in many studies. Here, indicate whether each material, system or method listed is relevant to your study. If you are not sure if a list item applies to your research, read the appropriate section before selecting a response.

Materials & experimental systems

n/a	Involved in the study
☒	☐ Antibodies
☒	☐ Eukaryotic cell lines
☒	☐ Palaeontology and archaeology
☒	☐ Animals and other organisms
☐	☒ Clinical data
☒	☐ Dual use research of concern
☒	☐ Plants

Methods

n/a	Involved in the study
☒	☐ ChIP-seq
☒	☐ Flow cytometry
☒	☐ MRI-based neuroimaging

Clinical data

Policy information about clinical studies

All manuscripts should comply with the ICMJE guidelines for publication of clinical research and a completed CONSORT checklist must be included with all submissions.

Clinical trial registration: NCT03150576

Study protocol: Available in supplementary materials and uploaded separately as a full protocol.

Data collection: Participants were assessed after consent at 29 UK centres between September 2016 to December 2021

Outcomes: The primary endpoint was pathological complete response (pCR), and secondary endpoints included event-free (EFS), and overall survival (OS).

Plants

Seed stocks: *Report on the source of all seed stocks or other plant material used. If applicable, state the seed stock centre and catalogue number. If plant specimens were collected from the field, describe the collection location, date and sampling procedures.*

Novel plant genotypes: *Describe the methods by which all novel plant genotypes were produced. This includes those generated by transgenic approaches, gene editing, chemical/radiation-based mutagenesis and hybridization. For transgenic lines, describe the transformation method, the number of independent lines analyzed and the generation upon which experiments were performed. For gene-edited lines, describe the editor used, the endogenous sequence targeted for editing, the targeting guide RNA sequence (if applicable) and how the editor was applied.*

Authentication: *Describe any authentication procedures for each seed stock used or novel genotype generated. Describe any experiments used to assess the effect of a mutation and, where applicable, how potential secondary effects (e.g. second site T-DNA insertions, mosiacism, off-target gene editing) were examined.*
