# Supplementary material for: The PARTNER trial of neoadjuvant olaparib with chemotherapy in triple-negative breast cancer
Source: Nature. 2024 Apr 8;629(8014):1142–8. doi: 10.1038/s41586-024-07384-2 (PMC11136660; doi:10.1038/s41586-024-07384-2)
Supplement: Supplementary file 4 — PARTNER Protocol [file 41586_2024_7384_MOESM4_ESM.pdf]

Partner

Platinum and PARP inhibitor for Neoadjuvant treatment of Triple NEgative and/or BRCA positive breast cancer

---

Trial Title:	Randomised, phase II/III, 3 stage trial to evaluate the safety and efficacy of the addition of Olaparib to platinum-based neoadjuvant chemotherapy in breast cancer patients with TNBC and/or gBRCA.
EudraCT Number:	2015-002811-13
Clinicaltrials.gov Number:	NCT03150576
IRAS number:	178681
Investigational Products:	Olaparib, Carboplatin, Paclitaxel
Protocol Version:	13.0, 07 July 2023 (PARTNER protocol only. Redactions relate to a confidential unreported substudy

Chief Investigator:	Prof Jean Abraham<br>The University of Cambridge, Department of Oncology<br>Addenbrooke's Hospital (Box 193-R4),<br>Hills Road, Cambridge, CB2 0QQ<br>✉ ja344@medschl.cam.ac.uk
Trial Sponsors:	Cambridge University Hospitals NHS Foundation Trust and<br>The University of Cambridge<br>R&D Department, Box 277, Addenbrooke's Hospital<br>Hills Road, Cambridge, CB2 0QQ<br>✉ cuh.research@nhs.net
SAE Reporting & Trial Coordination:	PARTNER Office – CCTU–Cancer Theme<br>Box 279 (S4), Addenbrooke's Hospital<br>Hills Road, Cambridge, CB2 0QQ<br>Direct line: 01223 348447 / 01223 348086<br>✉ cuh.partner@nhs.net

CANCER RESEARCH UK | CAMBRIDGE CENTRE

UNIVERSITY OF CAMBRIDGE

Cambridge University Hospitals NHS
NHS Foundation Trust

NCRI National Cancer Research Institute

1 Protocol Signatures

Chief Investigator

☐ I give my approval for the attached Protocol entitled "PARTNER, Platinum and PARP inhibitor for Neoadjuvant treatment of Triple NEgative and/or BRCA positive breast cancer", version 13.0 dated 07 July 2023

Name: Prof Jean Abraham

Signature: [redacted]

Date: 07 July 2023

Principal Investigator

I have read the attached Protocol entitled "PARTNER, Platinum and PARP inhibitor for Neoadjuvant treatment of Triple NEgative and/or BRCA positive breast cancer", version 13.0, dated 07 July 2023 and agree to abide by all provisions set forth therein.

☐ I agree to comply with the conditions and principles of Good Clinical Practice (GCP) and all applicable regulatory requirements.

☐ I agree to ensure that the confidential information contained in this document will not be used for any other purpose other than the evaluation or conduct of the clinical investigation without the prior written consent of the Sponsor

Name: ____

Signature: ____

Date: ____________________________

2 Trial Committees

Protocol Contributors
Chief Investigator: Jean Abraham
Trial Geneticist: Marc Tischkowitz
Trial Statisticians: Nikos Demiris and Alimu Dayimu
Trial Coordinators: Louise Grybowicz, = Erdem Demir, Karolina Lazarowicz, Camila Maidadepontes and Sonia Chukwuka
Trial Pharmacist: Anita Chhabra
Lead pathologist / UK Central Trial Pathologist: Elena Provenzano
Trial Clinical Research Fellows: Karen Pinilla and Rebecca Lucey

Trial Management Group
Protocol Contributors
Medical Oncologist with an interest in Genetics: Ellen Copson
Other Medical Oncologists: Anne Armstrong, Karen McAdam, and Rebecca Roylance.
Lead pathologist / UK Central Trial Pathologist: Elena Provenzano

Trial Steering Committee
Trial Management Group
Principal Investigators
Independent external clinician (Chair): Charlie Gourley

Independent Data and Safety Monitoring Committee
3 independent external clinicians: Judy Garber (Chair), Lajos Pusztai and Rita Nanda
1 independent external statistician: Mark Brady
1 Patient and Public Involvement representative: Safia Danovi

3 Table of Contents

1 Protocol Signatures 2
2 Trial Committees 3
3 Table of Contents 4
4 Abbreviations 9
5 Trial Synopsis 11
6 Trial Flow Charts 15
7 Introduction 18
8 Rationale underlying the PARTNER trial 19
[redacted]
9 Trial Design 28
9.1 Statement of design 28
9.2 Number of Centres 28
9.3 Number of Subjects 28
9.4 Duration 29
9.5 Trial objective 29
9.6 Trial Endpoints 29
9.6.1 Primary Endpoint 29
9.6.2 Secondary endpoints 30
9.6.3 Exploratory endpoints 30
10 Selection and withdrawal of subjects 31
10.1 Inclusion Criteria 31
10.2 Exclusion criteria 33
[redacted]
10.4 Registration & Central testing prior to Randomisation 36
10.4.1 gBRCA test 37
10.4.2 Tumour Infiltrating Lymphocytes (TILs) score 37
10.4.3 Assessment of the TNBC Basal phenotype 38
10.4.4 Registration and pre-screening 39
10.5 Screening 40
10.6 Treatment Assignment 42
PARTNER Randomisation 42
10.6.1 Method of Randomisation 42
10.6.2 Procedure of Randomisation 42
[redacted]
10.7 Discontinuation of trial treatment and patient withdrawal 44
10.7.1 Discontinuation of trial treatment 44
10.7.2 Withdrawal of consent 45
11 Trial Treatments 46
11.1 Treatment Summary 46
11.2 Maximum duration of treatment of a patient 48
11.3 Procedures for monitoring treatment compliance 48
11.4 Supply, accountability and dispensing 48
11.4.1 Pharmacy responsibilities 48
11.4.2 Drug Accountability 49
11.5 Olaparib 49

11.5.1 Legal Status 49
11.5.2 Description 49
11.5.3 Supply 49
11.5.4 Labelling 49
11.5.5 Storage Conditions 50
11.5.6 Returns and Destruction 50
11.5.7 Dose 50
11.5.8 Administration 50
11.5.9 Known drug reactions 50
11.6 Paclitaxel 51
11.6.1 Legal Status 51
11.6.2 Supply 51
11.6.3 Labelling 51
11.6.4 Storage Conditions 51
11.6.5 Dose 51
11.6.6 Administration 51
11.6.7 Known drug reactions & interaction with other therapies 52
11.7 Carboplatin 52
11.7.1 Legal Status 52
11.7.2 Supply 53
11.7.3 Label 53
11.7.4 Storage Conditions 53
11.7.5 Carboplatin dose 53
11.7.6 Administration 54
11.7.7 Known drug reactions & interaction with other therapies 54
[redacted]
11.10 Non-Investigational Medicinal Products 59
11.11 Concomitant therapy and washout Periods 59
11.11.1 Supportive therapies 59
11.11.2 Restricted concomitant medications 59
11.11.3 Wash-out periods 61
11.11.4 Food & Drink 61
11.11.5 Vaccines 61
11.11.6 Anticoagulant Therapy 61
11.11.7 Anti-emetics/Anti-diarrhoeals 61
11.11.8 Herceptin® (trastuzumab) 61
[redacted]

12 Management of toxicity 63
12.1 Maximum dose delay 63
12.2 G-CSF specific toxicity 63
12.3 Chemotherapy specific toxicity 63
12.4 Olaparib specific toxicity 66
[redacted]
13 Procedures and assessments 69
13.1 Prior to each cycle of Partner treatment 69
13.2 Prior to D8 and D15 of Paclitaxel 70
13.3 Mid-chemotherapy (After cycle 4, prior to cycle 5) 70
13.4 After end of chemotherapy and prior to surgery 70
13.5 After surgery 71
13.6 Long Term Follow-up 71
13.7 Clinical and radiological monitoring during neo-adjuvant chemotherapy 72
13.8 Surgery 73
13.9 Adjuvant Hormonal Treatment after Surgery 74
13.10 Radiotherapy 74
13.11 Disease Progression 74
13.12 Follow-Up and Relapse 74
13.13 Concurrent studies 75
14 Assessment of Safety and Safety Reporting 76
14.1 Definitions (table 10) 76
14.2 Evaluation and recording of adverse events (non-serious) 77
14.3 Adverse Events of Special Interest (AESI) 77
14.4 Reporting serious adverse events 77
14.4.1 General principles and responsibilities 77
14.4.2 Reporting period for SAEs 78
14.4.3 Evaluation of SAEs by the Principal Investigator 78
14.4.4 Table 11: Assessment of causality 78
14.4.5 Assessment of expectedness 79
14.4.6 Notification of SAEs 79
14.5 Reporting of Suspected Unexpected Serious Adverse Reactions (SUSARs) 80
14.5.1 Who should report and whom to report to? 80
14.5.2 When to report? 80
14.5.3 How to report? 80
14.6 Pregnancy Reporting 81

15 Evaluation of Results : Definitions and response / evaluation of outcomes measures 83
15.1 Pathological Complete Response 83
15.2 Overall Survival 84
15.3 Neurotoxicity* 84
15.4 Quality of life – Optional sub-study 84
15.5 Data related to Covid-19 85
16 Statistics 86
16.1 Sample size 86
16.2 Statistical Analysis methods 88
16.2.1 Analysis population 88
16.2.2 Statistical Analysis 88
16.2.3 Interim Analyses 89
16.3 Futility Analysis 89
16.4 Criteria for the premature termination of the trial 89

16.5 Definition of the end of the trial 90
17 Data handling and record keeping 91
17.1 Case Report Forms 91
17.2 Data quality assurance & monitoring 91
17.3 Source Data 91
17.4 Data Protection & Patient Confidentiality 91
18 Translational Studies and Sample collections 93
18.1 Mandatory blood samples for pharmacogenomic markers 94
18.2 Mandatory histopathology material and reports 94
18.3 Fresh Tissue samples collection 96
18.4 Sequential blood samples 96
18.5 Optional sequential microbiome samples sub-study 97
19 Trial conduct 98
19.1 Conduct of trial 98
19.2 Trial Committees 98
19.2.1 Trial Management Group (TMG) 98
19.2.2 Independent Data and Safety Monitoring Committee (IDSMC) 98
19.2.3 Trial Steering Committee (TSC) 98
19.3 Relationship between Trial Committees 99
20 Ethical & Regulatory considerations 100
20.1 Consent 100
20.2 Ethical committee review 100
20.3 Regulatory Compliance 100
20.4 Protocol Amendments 100
20.5 Peer Review 101
20.6 Declaration of Helsinki and Good Clinical Practise 101
20.7 GCP Training 101
20.8 Sponsorship, Financial and Insurance 101
20.9 Monitoring, Audit & Inspection 101
20.10 Protocol Compliance and Breaches of GCP 102
20.11 Publications policy 102
21 References 103
22 Appendices 106
Appendix 1: Synthetic Lethality 107
Appendix 2: ECOG performance status 107
Appendix 3: PARTNER -Timetable of events and investigations 108
Appendix 5: Schedule for follow-up visits 112
Appendix 6: TNM Staging System for Breast Cancer 113
*TO N1,N2 tumours must be histopathologically confirmed by LN biopsy (trucut or whole LN) Appendix 7: Criteria for BRCA1 and BRCA2 genes mutation testing within PARTNER (UK patients only) 1135
Appendix 8: Formulae, Calculations, and Abbreviations 116
Appendix 9: Contraception Guidelines 116
Appendix 10: Possible exclusion exceptions in cases of severe pre-existing sensory or motor disability 117
Appendix 11: Autoimmune or inflammatory disorders that exclude patients for inclusion into PartnerING- 118
Appendix 12 .... Guidelines regarding Olaparib /[redacted] potential interactions with concomitant medications 118
[redacted]

[redacted]
Appendix 14: Prolonged haematological toxicities while on study treatment. 127
Appendix 15: Actions Required in Cases of Increases in Liver Biochemistry and Evaluation of Hy’s Law 128
[redacted]

4 Abbreviations

AE	Adverse Event
AESI	Adverse Event of Special Interest
ALT	Alanine Aminotransferase
AML	Acute myeloid leukemia
ANC	Absolute Neutrophil Count
AR	Androgen Receptor
ATRi	Ataxia telangiectasia and rad3 related kinase
AST	Aspartate Aminotransferase
AUC	Area Under the curve
b.d.	bis die (twice a day)
BCRP	Breast Cancer Resistance Protein
BCSS	Breast cancer specific survival
BSA	Body Surface Area
CA	Competent Authority
CCTU-CT ("PARTNER Office")	Cambridge Clinical Trials Unit – Cancer Theme
CHK1	Checkpoint kinase 1
CI	Chief Investigator
CK5/6	Cytokeratin 5/6
CRF	Case Report Form
CRUK	Cancer Research UK
CT	Computerised Tomography
CTA	Clinical Trial Authorisation
CTCAE	Common Toxicity Criteria for Adverse Events
ctDNA	Circulating tumour DNA
CYP	Cytochrome P450
DCIS	Ductal Carcinoma in situ
DDR	DNA damage response
DFS	Disease Free Survival
DNA	DeoxyriboNucleic Acid
DSUR	Development Safety Update Report
ECG	Electrocardiogram
ECOG	Eastern Cooperative Oncology Group
EDTA	EthyleneDiamineTetraacetic Acid
EGFR	Epidermal Growth Factor Receptor
EOC	Epithelial Ovarian Cancer
ER	Oestrogen Receptor
FISH	Fluorescence in situ Hybridization
FNA	Fine needle aspiration
G	Grams
gBRCA	germline BRCA
GCP	Good Clinical Practice
G-CSF	Granulocyte-colony stimulating factor
GFR	Glomerular Filtration rate
GI90	Concentration giving 90% growth inhibition
GP	General Practitioner
H&E	Haematoxylin and eosin
Hb	Haemoglobin
HER2	Human Epidermal Growth Factor Receptor 2
HRD	Homologous Recombination Deficient
IB	Investigator's Brochure
IC90	Concentration giving 90% of the drug-induced inhibitory effect
IDSMC	Independent Data and Safety Monitoring Committee
IHC	Immunohistochemistry
IM	Intra-muscular

imAE	Immune-mediated Adverse Events
IMP	Investigational Medicinal Product
INR	International Normalised Ratio
I.V	Intravenous
Kg	Kilogram
L	Litre
LLN	Lower Limit of Normal
LN	Lymph node
LPBC	Lymphocyte Predominant Breast Cancer
MDS	Myelodysplastic syndrome
mcg	Microgram
mg	Milligram
MHRA	Medicines and Healthcare products Regulatory Agency
miRNA	MicroRNA
msec	Milliseconds
MRI	Magnetic Resonance Imaging
NB	Nota Bene
NCI	National Cancer Institute (USA)
NHS	National Health Service
OATP	Organic anion transporting polypeptide
ONS	Office of National Statistics
OS	Overall survival
PARP	Polyadenosine 5'diphosphoribose (PAR) polymerisation (PARP)
PARPi	Polyadenosine 5'diphosphoribose (PAR) polymerisation (PARP) Inhibitors (PARPi)
pCR	Pathological complete response
PD1	Programmed cell death protein
Pgp	Permeability glycoprotein
PI	Principal Investigator
PK	Pharmacokinetic
PLT	Platelets
PO	By mouth
PR	Progesterone Receptor
PS	Performance Status
QoL	Quality of Life
R&D	Research and Development
RCB	Residual Cancer Burden
REC	Research Ethics Committee
RFS	Relapse Free Survival
RRM	Risk Reducing Mastectomy
RNA	Ribonucleic Acid
RPA	Replication Protein A
RS	Replication Stress
RSI	Reference Safety Information
SAE	Serious Adverse Event
SAR	Serious Adverse Reaction
SC	Subcutaneous
SLN	Sentinel Lymph Node
SLD	Sum of Longest Single Diameter
SmPC	Summary of Product Characteristics
SUSAR	Suspected Unexpected Serious Adverse Reaction
TILs	Tumour Infiltrating Lymphocytes
TKIs	Tyrosine kinase inhibitors
TMG	Trial Management Group
TNM	Tumours Nodes Metastasis
TNBC	Triple Negative Breast Cancer
TOPBP1	DNA topoisomerase binding protein 1
TRICC	Translational Research in Clinical Trials Committee
TSC	Trial Steering Committee

TTSC	Time to Second cancer
UK	United Kingdom
ULN	Upper Limit of Normal
US FDA	United States Food and Drug Administration
WCBP	Women of Child Bearing Potential

5 Trial Synopsis

Title of clinical trial	Randomised, phase II/III 3 stage trial to evaluate the safety and efficacy of the addition of Olaparib to platinum-based neoadjuvant chemotherapy in breast cancer patients with TNBC and/or gBRCA.
Sponsor name	Cambridge University Hospitals Foundation NHS Trust and the University of Cambridge
EudraCT number	2015-002811-13
Disease under investigation	Breast Cancer
Purpose of clinical trial	**PARTNER** aims to establish if the addition of Olaparib to neoadjuvant platinum-based chemotherapy for Triple Negative Breast Cancer (TNBC) and/or germline BRCA (gBRCA) breast cancer is safe and improves efficacy.<br>[redacted]
Trial Design	**PARTNER** is an open label, randomised, 3-stage Phase II/III, with the optional pathway for patients with residual disease called PartnerING.<br>[redacted]
Primary Endpoint	**Stage 1:** Primary outcome measure – **safety** of the addition of Olaparib to three weekly Carboplatin/weekly Paclitaxel chemotherapy.<br>**Stage 2:** Primary outcome measure – pCR in each of the two research arms. At the end of stage 2, one of the research treatments will be dropped using the 'pick the winner' method.<br>**Stage 3:** Primary outcome measure - **pCR** at surgery after neoadjuvant treatment.<br>[redacted]
---	• pCR, pCR in breast alone and RCB<br>• Relapse-Free Survival (RFS)<br>• Breast cancer specific survival

	• Distant disease-free survival<br>• Local recurrence-free survival<br>• Overall survival<br>• Time to second cancer<br>• Radiological response after 4th and final cycles<br>• Treatment related toxicities<br>• Quality of life (sub-study)
Exploratory endpoints	Discovery and validation of prognostic, pharmacogenetic and pharmacogenomic markers that can be correlated with outcomes (pCR and RFS).<br>[redacted]
Sample Size	Minimum of 780 patients including:<br>• At least 478 TNBC non-gBRCA patients equally allocated to the control and the selected research arm.<br>and<br>• At least 188 gBRCA patients equally allocated to the control and the selected research arm.<br>• *A maximum of 15 evaluable patients to be allocated to PartnerING cohort B.*
Summary of eligibility criteria	**Inclusion criteria** (See section 10.1 for full criteria)<br>• Aged between 16 and 70 (at the time of the main Informed Consent).<br>• Written informed consent.<br>• Histologically confirmed invasive breast cancer.<br>• Clinical stage T1-4 N0-2 (tumour or metastatic node diameter > 10mm).<br>• Confirmed ER-negative and HER2- negative.<br>Or<br>• Germline BRCA mutation positive, irrespective of hormone status.<br>• Performance Status 0-1.<br>**Exclusion criteria** (See section 10.2 for full criteria)<br>• T0 tumour in absence of axillary node >10mm.<br>• TNBC with a non-basal phenotype and over-expressing Androgen Receptor.<br>• Not suitable for neoadjuvant chemotherapy.<br>• Distant metastases apparent prior to randomisation.<br>• Prior history of invasive breast cancer within the last 5 years.<br>• Any previous chemotherapy or targeted agent used for the treatment of cancer in the last 5 years.<br>[redacted]

	[redacted]<br><br>[redacted]
Investigational Medicinal Products, dosage and route of administration	**4 cycles of:**<br>**Paclitaxel (IMP):** I.V, 80mg/m^2, on Days 1, 8 & 15 every 3 weeks<br>**Carboplatin (IMP):** I.V, AUC 5 on Day 1 every 3 weeks<br>**+/-**<br>**Olaparib (IMP):** oral, 150mg b.d. for 12 days, every 3 weeks<br>Research Arm 1: Olaparib on Day -2 to Day 10 *(dropped after stage 2)*<br>Or Research Arm 2: Olaparib on Day 3 to Day 14 *(selected after stage 2)*<br><br>[redacted]-[redacted]<br><br>[redacted]
Non Investigational Medicinal Products	Prophylactic **G-CSF** to be given as per local practice during PARTNER cycles 1-4, and in response to clinical need during all subsequent cycles<br>and<br>3 cycles of **anthracyclines** as per local practice.
Treatment period	A minimum of 21 weeks of chemotherapy followed by surgery.<br>[redacted]
Procedures:<br><br>Screening & enrolment	Eligible patients with early breast cancer will be registered and consented for screening:<br>BRCA mutation test<br>Tumour Infiltrating Lymphocytes (TILs) score<br>CK5/6 and/or EGFR and if appropriate Androgen receptor status by IHC<br>Standard assessment prior to chemotherapy<br>Standard staging to exclude metastatic disease<br>When eligibility is confirmed, patients will be randomised via a web-based central system which will allocate each patient a unique randomisation number associated with one of the treatment arms.<br>*Patients showing **residual disease** on biopsy after Cycle 6 of chemotherapy will be centrally assessed for biomarkers expression (TC*

	and TILs). [redacted]
End of Trial	For patients, the end of trial is after the last follow-up visit or contact with the research team planned 10 years after surgery.
Procedures for safety monitoring during trial	The Trial Management Group and the Independent Data and Safety Monitoring Committee will regularly review the patient safety data.<br>[redacted]<br>Pharmacovigilance will be performed by the PARTNER Trial Office.
Criteria for discontinuation of trial treatment on safety grounds	Severe toxicity or inter-current illness, requiring cessation in the judgement of patient's clinician.<br>Patient within 4 weeks has not recovered from toxicity to an extent that allows further treatment.<br>Patient unable to comply with trial procedures.<br>Disease progression while on trial treatment.<br>Patient becomes pregnant.<br>Patient choice.<br>Patient ineligible.

6 Trial Flow Charts

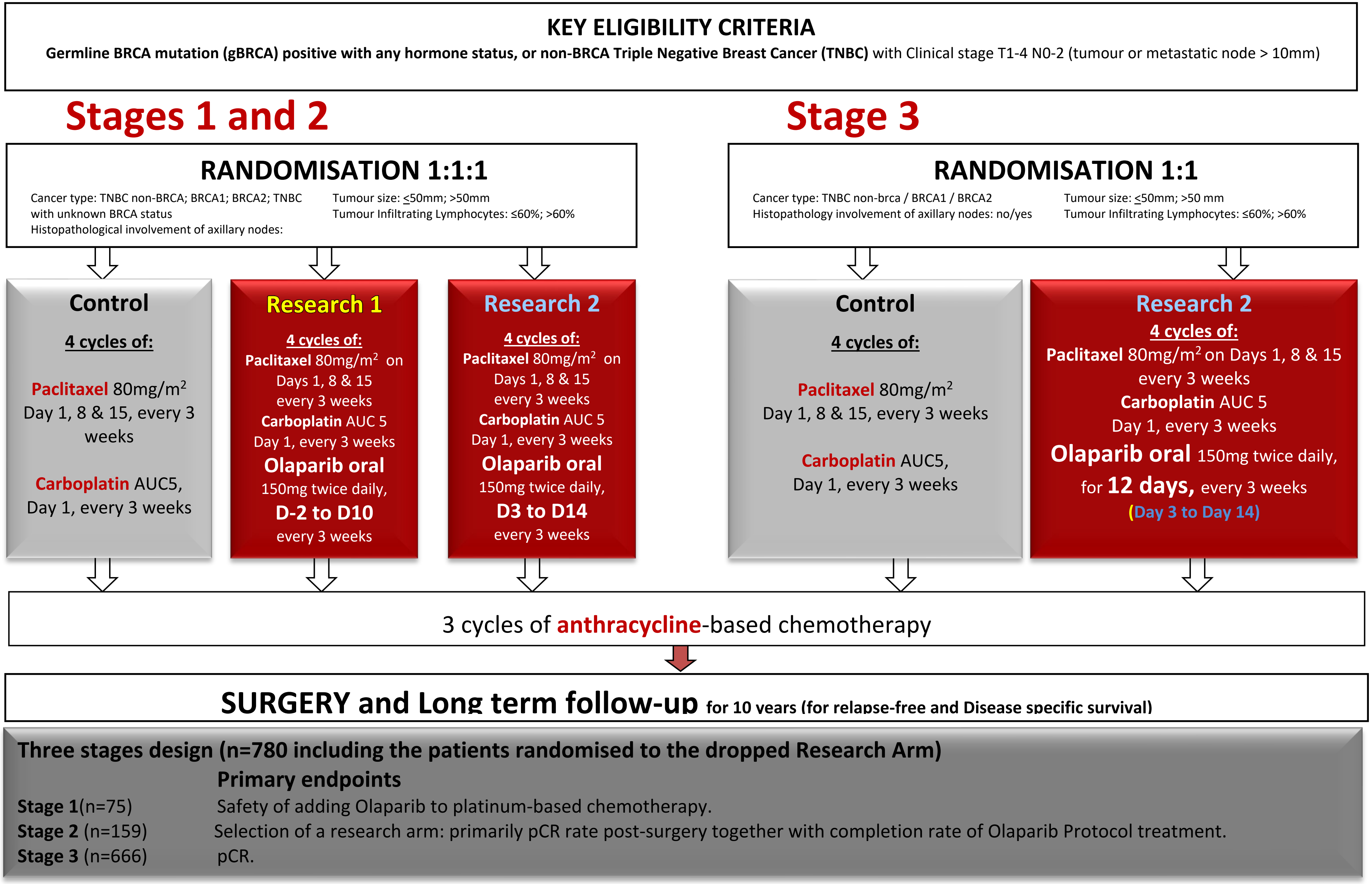

[redacted]

[redacted]

7 Introduction

Breast cancer is the most common female cancer worldwide, and the second most common cancer overall, with more than 1,676,000 newly diagnosed in 2012 (25% of female cancer cases and 12% of the total) [1]. Although advances in screening, surgical intervention, radiotherapy and systemic therapies have improved survival, worldwide approximately 522,000 women died as a result of their breast cancer in 2012 [2].
Breast cancer is a heterogeneous disease and there are many ways to classify it, including using traditional histopathological features, immunohistological and molecular classifications [3].

Triple Negative Breast Cancers (TNBC)

TNBC is defined by absent or reduced immunohistochemical expression of Oestrogen Receptor (ER) and Progesterone Receptor (PR) and HER2 (Human Epidermal Growth Factor Receptor 2). Approximately 12-17% of breast cancers are regarded as TNBC [4]. TNBC occurs more frequently in younger or pre-menopausal women and overall has a worse prognosis than other breast cancer sub-groups [5]. These patients are at highest risk of relapse during the first 3-5 years post diagnosis. However, for patients with TNBC, without recurrence in this timeframe, distant relapse is less common. In neoadjuvant studies, patients with TNBC have increased pathological Complete Response (pCR) rates compared with non-TNBC, and those with pCR have excellent survival. However, patients with residual disease after neoadjuvant chemotherapy have significantly worse survival if they have TNBC compared with non-TNBC, particularly in the first 3 years [5]. Thus, although TNBC is a biologically diverse and aggressive sub-group, early effective treatment can lead to cure [4]. Current standard treatment for early stage TNBC is systemic chemotherapy either pre- or post-definitive surgical management. There are no specific targeted therapies available for this sub-group.
TNBC is a heterogeneous sub-group and molecular classification of TNBC has led to the development of at least seven TNBC sub-types [6]. A validation study of these sub-types indicates that the likelihood of pCR to neoadjuvant therapy is sub-type dependent [7]. Studies have also shown that there are phenotypic and molecular similarities between *BRCA1*-associated breast cancer and sporadic TNBC. In addition, 10% to 20% of TNBC harbour germline *BRCA* (gBRCA) mutations [8,9]. Our understanding of the relationship between specific TNBC sub-types and *BRCA*-associated breast cancer is still evolving and is investigated in the translational component of this trial.

BRCA

BRCA1 and BRCA2 are tumour suppressor genes. Both genes encode proteins which work at different stages in the DNA damage response and DNA repair pathways. Inheritance of a mutation in BRCA1 or BRCA2, is associated with early-onset breast cancer and also an increased risk of ovarian, pancreatic, stomach, laryngeal, fallopian tube and prostate cancer. Approximately 5-7% (BRCA1 ~3-4%: BRCA2 ~2-3%) of all breast cancer cases are associated with BRCA mutations [10]. Carriers of BRCA mutations have a lifetime risk of developing breast cancer of BRCA1 ~60-70% and BRCA2 ~40-60%, respectively [11]. The lifetime risk of ovarian cancer is BRCA1 ~20-45% and BRCA2 ~10-20% respectively. As described earlier TNBC are often BRCA1 and ~70% of BRCA1-related breast cancers are TNBC, whereas BRCA2 carriers may present with hormone receptor positive breast cancers. However, 16% of BRCA2 carriers also have TNBC [12]. Mutations in both BRCA genes within one patient are rare [13]. There is strong evidence to suggest that chemotherapy is beneficial to patients with BRCA mutated breast cancer [14]. The current chemotherapy treatment options for BRCA positive patients presenting with early breast cancer are as standard treatment for any high risk breast cancer and with no specific targeted agents.

Neoadjuvant Chemotherapy

Existing evidence already demonstrates that for breast cancer chemotherapy neoadjuvant and adjuvant therapy appear equivalent in terms of survival and overall disease progression, assuming adequate surgery is completed in both settings [15]. Neoadjuvant chemotherapy may improve surgical outcomes in patients for whom a primary surgical approach is technically difficult and in patients with operable breast cancer who prefer breast conservation. It also allows early evaluation of the effectiveness of systemic therapy and provides an opportunity to access the biological samples required to undertake translational research.
A recent meta-analysis from the US Food and Drug Administration (FDA) [16] confirmed the strong correlation between the achievement of pCR and improvement in disease free survival (DFS) and overall survival (OS) at an individual patient level. The FDA has also proposed [17] that the early primary endpoint of pCR in neoadjuvant trials in highly proliferative breast cancers (ER negative, HER2 positive, high grade, and gBRCA) can now contribute data for accelerated licensing approval for new targeted agents.
There has been considerable recent debate around the issue of correlation between primary endpoint results (pCR) in neoadjuvant trials and secondary, longer term endpoints of progression-free and overall survival. Pusztai and colleagues [18] have drawn attention to the complexity of this relationship and the multiple 'confounders' which are introduced, not only by way of the traditional definitions of DFS and OS, but also by breast cancer intrinsic subgroup and post-surgical adjuvant treatment. What is not in dispute, however, is that achieving a significantly higher percentage rate of pCR is an indication of significant benefit for individual patients in stratified sub-groups.

8 Rationale underlying the PARTNER trial

Use of Platinum Agents in TNBC and BRCA Positive Breast Cancers

Several studies have now provided evidence that supports the use of platinum-based chemotherapy as part of the first line treatment of Homologous Recombination Deficient (HRD) patients, and potentially also with TNBC patients (Table 1). Byrski [19] reported a pCR rate of 83% with neoadjuvant cisplatin chemotherapy in gBRCA1 patients, in a small (n=12) non-randomised retrospective analysis. Von Minckwitz [20] reported the GeparSixto trial, with an increase in pCR from 36.9% to 53.2% (p=0.005) with the addition of platinum to standard neoadjuvant regimens in TNBC patients. In the CALGB 40603 neoadjuvant study, Sikov [21] confirmed that pCR increases to 49% (where pCR is defined as previously - absence of invasive disease in breast and axillary lymph nodes) in stage II-III TNBC with the addition of Carboplatin to weekly Paclitaxel, in a 2 x 2 factorial design with Bevacizumab. gBRCA patients may achieve higher pCR rates of 57.9%, data presented by von Minckwitz, ASCO 2014 [22].

Selection of chemotherapy backbone for this neoadjuvant trial

The selection of the agents and doses has been developed using data from CALGB 40603 [21] neoadjuvant TNBC trial [20] and doses have also been informed by the ICON8 trial [23]. CALGB 40603 used 3 weekly Carboplatin AUC 6 and Paclitaxel 80mg/m^2 weekly (3weeks). However, due to the potential for myelosuppression in combination with Olaparib, the Carboplatin dose has been reduced to AUC 5 for the PARTNER trial. ICON8 is a randomised 3-arm, 3-stage phase III trial to evaluate the safety and efficacy of dose-dense, dose-fractionated Carboplatin-Paclitaxel chemotherapy in the first-line treatment of ovarian cancer. ICON8 uses Paclitaxel 80mg/m^2 weekly with Carboplatin AUC 5 x 3 (3 weeks) and although the early safety data on arm 3 show that the dosing is safe and well tolerated, the ICON8 Independent Data and Safety Monitoring Committee (IDSMC) report has recommended the use of prophylactic Granulocyte Colony Stimulating Factor (G-CSF) with each cycle.

In addition the ISPY2 trial has used a 3 weekly regimen of Carboplatin and weekly Paclitaxel with a PARP inhibitor Veliparib in a neoadjuvant phase II successfully (in the TNBC cohort pCR 51% PARP inhibitor arm vs 26% standard treatment arm) and without excessive toxicity [24].
Combining data from both these studies, in the PARTNER trial we will use Carboplatin AUC5 three weekly and Paclitaxel 80mg/m^2, weekly for 12 weeks. In addition the trial mandates using the G-CSF as primary prophylaxis as per local practice, unless there are any contra-indications or due to cessation for clinical reasons in the judgement of the clinician caring for the patient. G-CSF has previously been used with Olaparib and chemotherapy combinations without problems [25].

Table 1: pCR in Neoadjuvant regimens for TNBC

Chemotherapy Regimen	TNBC	pCR (%)	pCR definition	Reference
Anthracycline/taxane				
a. FEC/FAC/AC	14	20% (p=0.0001)	pCR breast and axilla	Liedtke et al [5]
b. TFEC/TFAC	35	28% (p=0.0071)	pCR breast and axilla	Liedtke et al [5]von
c. Anthracycline/taxane	542	34% (p<0.001)	pCR breast and axilla	Minckwitz et al [26]
Platinum alone				
a. Cisplatin (BRCA1/non-BRCA1)	28 (2 BRCA1 / 26 non-BRCA)	Overall pCR 21% (pCR: 2BRCA 100% & non-BRCA=17%)	pCR breast	Silver et al [27]
b. Cisplatin (and other regimens in BRCA1)	102 (all BRCA1)	Overall pCR 24% (pCR 83% in 12 platinum treated BRCA1)	pCR breast and axilla	Bryski et al [19]
Platinum and taxane				
a. Paclitaxel/Carboplatin	12	67%	pCR breast and axilla	Sikov et al [28]
b. Paclitaxel/Carboplatin	24	33%	pCR breast and axilla	Chen et al [29]
c. Docetaxel/Carboplatin	13	77%	pCR breast	Kern et al [30]
d. Docetaxel/Carboplatin	9	44%	pCR breast and axilla	Roy et al [31]
e. Docetaxel/Carboplatin	11	55%	pCR breast	Chang et al [32]
f. Paclitaxel/Carboplatin	595*	53.2% vs 36.9% (Carbo vs Non-carbo)	pCR breast and axilla	von Minckwitz et al [20]
g. Paclitaxel/Carboplatin	443	54% vs 41% (Carbo vs Non-carbo)	pCR breast and axilla	Sikov et al [21]

*This trial included TNBC and HER2+ve breast cancer. TNBC received: 158 Carboplatin vs 157 Non-Carboplatin. Carboplatin was reduced from AUC 2·0 to 1·5 due to haematological toxicity. †38 BRCA mutation carriers (35 BRCA 1, 3 BRCA 2) pCR increased to 57.9% for patients with gBRCA mutation – ASCO abstract 2014 [22]. *Table adapted from von Minckwitz 2012* [33]

Use of Polyadenosine 5'diphosphoribose (PAR) polymerisation (PARP) Inhibitors (PARPi) in BRCA Positive Breast Cancers

PARP enzymes play an important role in the repair of single strand breaks. PARPi target DNA homologous repair pathways, by preventing repair of single strand breaks leading to problems downstream with double strand repair. PARPi therefore work in synergy with DNA damaging agents like platinums, which also cause strand breaks.
In patients with BRCA mutations, and potentially other patients with HRD, the already compromised homologous repair pathways allow PARPi to work particularly effectively leading to "synthetic lethality" (see Appendix 1).
There is strong evidence of efficacy for single agent PARPi in metastatic gBRCA patients with substantial prior chemotherapy exposure [34]. Both, Phase III -OlympiAD and Phase III- EMBRACA studies support the hypothesis that breast cancers with defects in a specific DNA damage repair pathway are sensitive to a PARPi. Several additional studies are investigating combined platinum and PARPi therapy either given concurrently or sequentially in the metastatic setting.

Use of Polyadenosine 5'diphosphoribose (PAR) polymerisation (PARP) Inhibitors (PARPi) in Triple Negative Breast Cancer (TNBC)

The majority of clinical trial evidence supporting the use of PARP inhibition in breast cancer was initially limited to BRCA mutated breast cancer tumours, however, it is clear that there are similarities in the biological behaviour of certain types of TNBC and BRCA mutated cancers. If assessed within an appropriately powered, prospective clinical trial this would potential broaden the scope of use of PARPi. Hence, a better understanding of molecular features and a more comprehensive and independent analysis of BRCA and non/BRCA (TNBC) population is also required [3,4].

Increasingly the data suggests that HRD is an important element of TNBC biology and it is not only explained by loss of BRCA1 and BRCA2 function. Defects in HR repair can arise through other mechanisms, thus, resulting in a BRCA-like phenotype [5]. The common term "genomic scars", typically characterized in BRCA tumours has also been identified in tumours BRCA wild type [6]. Typical rearrangement signatures with high numbers of tandem duplications have been linked to a subgroup of basal like TNBC with HRD profile [7]. This suggest that TNBC involves a targetable molecular group outside the BRCA population that could also benefit from specific targeted therapies.

PARPi have been tested in **sporadic Triple Negative (non-BRCA) metastatic** patients with promising results. Olaparib has been used in combination with different chemotherapy types and schedules. When combined with Paclitaxel, it demonstrated encouraging response rates of ~37%. [8] In combination with platinum, it showed responses of 22% and 41%. Responses were durable with a median treatment duration of 52 weeks. [9,10] A variety of PARPi and combinations are currently tested in this setting. (e.g. NCT03167619, NCT03367689, NCT0282612, NCT267889, NCT02849496, NCT2595905, NCT2401347, NCT02505048, NCT01482715).

Following the results of the I-SPY2 trial reported by Rugo et al[24], the BrighTNess trial evaluated the additional benefit of a PARPi /Veliparib given in combination with platinum in the early setting. [12] Although the trial failed to show added benefit from Veliparib, several other PARPi with different activity profiles still appear as promising therapies to increase responses in BRCA and non-BRCA triple negative tumours.

Given the potential similarities in TNBC and gBRCA breast cancers, the inclusion of TNBC in this trial would also allow molecular comparisons in terms of both biology and response to treatment. A clear and comprehensive evaluation of the biological and clinical features of each independent group will increase the general understanding of this disease, will lead to better, more effective interventions and potentially a targeted therapy treatment for patients with limited treatment options.

Toxicology, Safety, Pre-clinical and Clinical Experience with Olaparib

Toxicology, Safety and Pre-clinical experience: The non-clinical safety evaluation studies conducted with Olaparib (including single and repeat dose toxicity studies of up to 6 months duration in rats and dogs, genotoxicity studies and reproductive toxicology studies in rats) support the use of Olaparib in the treatment of cancer patients. Further details are available in the Olaparib Investigators Brochure (IB).

Clinical experience: Tolerable regimens of Olaparib with a combination of chemotherapy agents (Paclitaxel/Carboplatin; Paclitaxel; Carboplatin and liposomal doxorubicin) have been established, which supports further combination studies. A summary of all the relevant clinical trials data relating to Olaparib is available in the most recent version of the Olaparib IB.

Justification for the starting dose of the PARP inhibitor and evidence to support scheduling, safety and tolerability of the treatment

Data from a phase Ib/II study by Rivkin et al [35] , presented at ASCO 2014, demonstrates that Olaparib 150mg b.d. days 1-3 weekly for 4 cycles, with Carboplatin AUC 2 and Paclitaxel 60mg/m^2 weekly (x 3 q 4 weeks) in heavily pre-treated metastatic ovarian cancer patients, was safe, tolerable and effective. We have used data from this trial in combination with data from the CALGB 40603 trial to define the proposed investigational arm of the trial. Our proposed trial is in chemotherapy-naïve early breast cancer patients and uses the same dose of Olaparib [150mg b.d. for 12 days (D-2 to D10 or D3 to 14) for 4 cycles (12 weeks)]. The patients therefore receive an equivalent Olaparib dose to that delivered in the Rivkin study. The chemotherapy backbone is Carboplatin AUC 5 q3 weeks x 4 with concurrent Paclitaxel 80mg/m^2 weekly for 12 weeks. The Olaparib dose used in our trial is similar to the well-tolerated dose identified in this phase Ib/II trial, whilst the Carboplatin dose is given as a 3 weekly regimen which was shown to be associated with less overall toxicities when comparing the GeparSixto and CALGB trials. The Paclitaxel dose is higher than in the Rivkin study [35], but is the same as was used in both the GeparSixto and CALGB trials. The IB also contain details of breast cancer studies and other solid tumour studies in the phase I/II setting that have used Olaparib in combination with chemotherapy agents including platinum agents and Paclitaxel [e.g. Study D0810C00011 (Olaparib + Paclitaxel); Study D0810C00041 (Olaparib + Carboplatin + Paclitaxel); Study D0810C00039 (Olaparib + Paclitaxel)].
Data to support the potentiation of platinum agents with Olaparib has shown that when BRCA2 deficient mouse mammary cell lines were assessed in combination studies it showed that for BRCA2 deficient cells a synergistic potentiation of platinum toxicity with Olaparib whereas the interaction was only additive in the BRCA2 proficient cells [36].

The timing of Olaparib with 3 weekly Carboplatin and weekly Paclitaxel has been discussed in considerable detail within the Trial Management Group (TMG) and with the Olaparib Team at AstraZeneca. Although there is extensive data on multiple schedules with Olaparib both alone and in combination with chemotherapy, it is not clear what the optimal schedule to achieve synergism between Carboplatin and Olaparib would be. We have therefore decided to compare two schedules of Olaparib within the trial. In one research arm the Olaparib schedule will start 2 days before the Carboplatin for 12 days i.e. D-2 to D10. This is based on the hypothesis that the use of Olaparib would inhibit PARP even before DNA damage from Carboplatin had occurred, which might be more effective and have greater efficacy. In a second research arm, the Olaparib schedule will start 48 hours after the Carboplatin (for 12 days i.e. D3 to D14) to ensure that the Carboplatin has by then damaged significantly the DNA and the Olaparib will prevent the activity of the DNA repair mechanisms. There is toxicology data from pre-clinical studies from rat research that indicates that allowing a 48 hour break between administration of Carboplatin and then Olaparib might reduce the myelosuppressive effects [37]. However, there is no data on how this may impact on efficacy. The dosing of Olaparib will be 150mg b.d. for 12 days. The total dose of Olaparib will therefore be just above 25% of the full single agent maintenance dose which is licensed (300mg b.d. continuous) and in line with the Rivkin study dosage, which was found to be safe and tolerable in multi-agent treated patients.

Upfront BRCA Mutation Testing

Upfront BRCA Mutation Testing will be encouraged initially and will become mandatory pre-randomisation later in the trial. At the start of the trial patients will be randomised and BRCA tested, they can be randomised before their BRCA status is known. Once all TNBC patients are recruited (see section 16.1) then all patients will need to be tested upfront and results of the BRCA test will be required prior to randomisation.

Considering an order of 5% of additional patients is planned in the trial, there will be very limited impact on the trial should the proportion of gBRCA patients between control and research arms have been imbalanced, prior to this.

We have presented the concept of patients to consent for upfront BRCA1/2 testing and the trial design to patient advocate groups to assess patient acceptability. Their feedback was that testing without formal counselling, followed by appropriate and full counselling for those where a mutation or variant of unknown significance is found, is acceptable.
In addition, there are a number of initiatives to streamline genetic testing: A clinical study in patients with epithelial ovarian cancer called Genetic Testing in Epithelial Ovarian Cancer, (https://medgen.medschl.cam.ac.uk/research/gteoc-study/), in which patients are tested upfront for BRCA1/2 mutations (without formal counselling) soon after diagnosis [38].
Mainstreaming genetics and genetic testing is likely to become more common place as treatments become increasingly directed by genetic test results.

We propose for the first time to treat Triple Negative Early Breast Cancers (TNBC) with neo-adjuvant chemotherapy composed of Carboplatin and Paclitaxel+/- a PARP inhibitor (Olaparib) in order to investigate the safety profile of the combination of these 3 drugs as well as its efficacy.

[redacted]

[redacted]

[redacted]

[redacted]

[redacted]

[redacted]

[redacted]

[redacted]

[redacted]

[redacted]

[redacted]

[redacted]

[redacted]

[redacted]

[redacted]

[redacted]

[redacted]

[redacted]

[redacted]

[redacted]

[redacted]

[redacted]

[redacted]

[redacted]

[redacted]

[redacted]

[redacted]

[redacted]

[redacted]

[redacted]

[redacted]

9 Trial Design

9.1 Statement of design

This is an open label, **2-arm, 3-stage, randomised, phase II/III** trial to evaluate (i) safety and (ii) efficacy, of the addition of Olaparib to platinum-based neoadjuvant chemotherapy in breast cancer patients with TNBC and /or gBRCA.

PartnerING pathway is a phase II open label, 1 cohort, Simon two-stage design. It involves the addition of two novel targeted agents with an adequate safety profile. If the cohort B is deemed futile by trial criteria, it will not proceed to Stage 2 of the pathway.

9.2 Number of Centres

This will be an international multi-centre trial. Approximately 60 sites are expected to take part.

9.3 Number of Subjects

The trial is to recruit a minimum of 780 patients in total including a minimum of 188 patients with gBRCA breast cancer (see Section 16.1 for details).

PartnerING is to recruit a maximum of 15 evaluable patients to cohort B (see Section 16.1 for details). For cohort B, 10 patients will be included in Stage 1 and at least 1 RCB-0 or RCB-I will be required after the completion of Stage 1 to continue to Stage 2. Stage 2 will recruit a maximum of a further 5 patients and at least 1 RCB-0 or RCB-I will be required after the completion of Stage 2 for the trial to be successful.

9.4 Duration

It is intended that each subject on the PARTNER trial will receive 21 weeks of neoadjuvant chemotherapy +/- 12 weeks of Olaparib, followed by breast surgery.

Patients included into PartnerING are due to receive 8 weeks of additional treatment prior to surgery.

Long term follow-up (10 years after surgery) is planned for the secondary outcome measures of survival.

9.5 Trial objective

This neoadjuvant trial for patients with TNBC and/or gBRCA breast cancer, aims to investigate the safety and efficacy (improvement in pCR) of concurrent platinum-based chemotherapy with Olaparib (PARPi).

[redacted]

9.6 Trial Endpoints

Definition

pCR/RCB-0 (Pathological Complete Response) after neoadjuvant chemotherapy defined as no residual invasive carcinoma within the breast (Ductal Carcinoma *in situ* (DCIS) is permitted) AND no evidence of metastatic disease within the lymph nodes. This will be assessed by a central review of the surgery pathology reports (2 readers), see Section 15.1.

MRD (Minimal Residual Disease) is defined as RCB-I. This calculation is based on four parameters of residual tumour (residual invasive tumour size, percentage cellularity of invasive carcinoma, number of axillary lymph nodes containing metastatic carcinoma, and diameter of the largest metastasis in an axillary lymph node).

9.6.1 Primary Endpoint

Stage 1: Primary outcome measure – **safety** of the addition of Olaparib to weekly Paclitaxel and 3 weekly Carboplatin chemotherapy. This will be measured using NCI CTCAE v4.03.

Stage 2: **Selection of a research arm:** primarily pCR rate post-surgery, together with completion rate of Olaparib protocol treatment. At the end of stage 2, one of the research treatment arms will be dropped using the 'pick-the winner' method. Recruitment will continue while waiting for results and analyses.

Stage 3: Primary outcome measure - **pCR** at surgery after neoadjuvant treatment.

PartnerING:

*Primary outcome measure – **RCB-0 and RCB-I** (Residual Cancer Burden class 0 and I) rate at surgery after the administration of 2 cycles of a combination of new agents (AZD6738-Durvalumab).*

9.6.2 Secondary endpoints

- pCR at surgery assessed by central pathology review of the diagnosis and surgery slides and Residual Cancer Burden (RCB) assessed by central review of the surgery slides.
- Relapse Free Survival (RFS), calculated from date of randomisation to date of the first relapse or date of death from all causes, whichever occurs first.
- Breast cancer specific survival (BCSS), calculated from date of randomisation to date of death from breast cancer.
- Distant disease-free survival, calculated from date of randomisation to date of the first distant disease relapse or date of death from all causes, whichever occurs first.
- Local recurrence-free survival, calculated from date of randomisation to date of the first local-recurrence or date of death from all causes, whichever occurs first.
- Overall survival (OS), calculated from date of randomisation to date of death from all causes.
- Time to second cancer (TTSC), calculated from the date of randomisation to the date of diagnosis of second cancer.
- pCR in breast alone.
- Residual Cancer Burden (RCB) I-III will be assessed by central pathology review, see section 15.1.
- Radiological response after 4th and final cycles.
- Treatment related toxicities.
- Quality of Life (sub-study, see section 15.4).

9.6.3 Exploratory endpoints

Discovery and validation of prognostic, predictive, pharmacogenetic and pharmacogenomic markers that can be correlated with outcomes (pCR and RFS) in patients randomised to receive Olaparib compared with those who were not.

[redacted]

- [redacted]
- [redacted]

Please refer to section 18 for further details.

10 Selection and withdrawal of subjects

10.1 Inclusion Criteria

- Aged between 16 and 70 at time of the main Informed Consent.
- Written informed consent, willing and able to comply with the Protocol for the duration of the trial including undergoing treatment and scheduled visits and examinations.
- Histologically confirmed invasive breast cancer.
- **ER-negative*, and HER2-negative**** breast cancer (TNBC, non-BRCA). Patients will be eligible with any PR status but **PR expression must be scored**.

  * ER and PR negative defined as IHC nuclear staining <10% or Allred score 0-3.
  **HER2 negative (not eligible for anti-HER2 therapy) defined as:
  -IHC 0, 1+ without ISH OR
  -IHC 2+ and ISH non-amplified with ratio less than 2.0 and copy number ≤6 OR
  -ISH non-amplified with ratio less than 2.0 and copy number ≤6
  ** Cancers with ratio < 2 but copy number > 6 are regarded as HER2 positive.
  ** The HER2 copy number should always be reported under ASCO/CAP and UK guidelines

  OR

  Germline BRCA (gBRCA) mutation positive, HER2 negative, and PgR / ER of any status.
  Note: mutation in BRCA1 or BRCA2 must be documented and predicted to be detrimental / lead to loss of function.

- T1c, T2 or T3 tumours (>10mm diameter; see Appendix 6).

  OR

  T4 tumour of any size with direct extension to (a) chest wall or (b) skin.

  OR

  Inflammatory carcinoma with tumour of any size.

  OR

  Other Locally Advanced Disease:

  - Involvement of ipsilateral large or fixed axillary lymph nodes, or infra or supraclavicular nodes (>10mm diameter or clinical N2 or N3, see Appendix 6) and primary breast tumour of any diameter.
  - Involvement of ipsilateral large or fixed axillary lymph nodes, or infra or supraclavicular nodes (>10mm diameter, or clinical N2 or N3, see Appendix 6), without a primary breast tumour identified, the presence of breast cancer in a Lymph Node (LN) must be histopathologically confirmed by LN biopsy.

  OR

  Multifocal tumour:

  - with at least one tumour with a size>10mm.

- During phase 1 of the trial, non-BRCA patients with multifocal disease were eligible to enter the trial provided that these foci are TNBC and one of them met the size criteria above. If a patient was thought to have unifocal disease at diagnosis and then was later found to be multifocal they could remain within the trial as long as no new foci were HER2 positive. Patients with bilateral disease were eligible to enter the trial provided they were either BRCA positive, or that both breast disease were HER2 negative and at least one of them met the size criteria above and is TNBC. NOTE: This no longer applies as phase 1 of the trial has now closed.

- Be fit to receive the trial chemotherapy regimen in the opinion of the responsible clinician:

  - Adequate bone marrow•, hepatic••, and renal function•••. Haematological values should meet the criteria below prior to commencing treatment. *Note: patients with GFR <51ml/min are not eligible*

Recommendations for PARTNER:

•Bone marrow function: Hb ≥100g/L; absolute neutrophil count ≥1.5 x 10^9/L; platelets 75 x 10^9/L if no active bleeding (values relate to before commencing treatment).

••Adequate Hepatic function:
- AST/ALT ≤2.5x ULN
- Alkaline phosphatase ≤2 x ULN
- Bilirubin within normal range i.e. ≤ 1.5 x ULN. If AST/ALT and Alkaline phosphatase are within normal limits then isolated elevation of bilirubin to 3≤ ULN and a presumptive diagnosis of Gilbert's syndrome is permitted.

•••Renal function: Creatinine ≤1.5 x ULN (see Appendix 8 for Cockcroft and Gault formula).

  - ECOG performance status of 0, or 1 (see Appendix 2).

- **Treatment should be commenced within 6 weeks** of the diagnostic biopsy. In uncommon circumstances, where medically acceptable and after PARTNER team approval, treatment is permitted to start within a maximum of 9 weeks of the diagnostic biopsy.

- Tumour Infiltrating Lymphocytes score (see section 10.4.2).

- CK5/6 and/or EGFR +/- Androgen Receptor IHC score if patient is non-brca TNBC.

- Availability of slides and paraffin embedded tissue blocks from pre-treatment core biopsy and from primary surgical resection.

- Women of child-bearing potential (WCBP), [defined as not surgically sterilized or not post-menopausal for at least 24 consecutive months if age ≤55 year or 12 months if age >55 years] must have a negative serum pregnancy test within 14 days prior to randomisation. Once a negative pregnancy test is received the patient must be informed that they must use adequate contraception for at least 6 months after the last dose of the trial treatment.

- All WCBP and all sexually active male patients and their partners must be aware that they should not conceive during the treatment period and therefore must use effective forms of contraception, throughout their participation in the trial and for at least 6 months after the last dose of trial treatment. Please follow the contraception guidelines in appendix 9.

10.2 Exclusion criteria

The presence of any of the following will exclude patients:

- T0 tumour in absence of axillary node >10mm.
- **TNBC (non-BRCA) with a non-basal phenotype which strongly expresses Androgen Receptor** (for further details, please refer to section 10.4.3).
- **Triple negative subtypes such as adenoid cystic, apocrine, metaplastic, low grade adenosquamous, secretory carcinoma or salivary type triple negative tumours.**
- Patients diagnosed of ipsilateral synchronous ER positive (Allred Score >3) breast cancer tumours (known at inclusion) in absence of germline BRCA mutation.
- Previous or concomitant chemotherapy or biological agents used for the treatment of cancer in the last 5 years, however, extended endocrine therapy is acceptable, e.g. 5 years of Tamoxifen.
- Malignancy within the last 5 years except: adequately treated non-melanoma skin cancer; curatively treated in situ cancer of the cervix; bilateral ductal carcinoma in situ (DCIS); Stage 1, grade 1 endometrial carcinoma; or other solid tumours including lymphomas (without bone marrow involvement) curatively treated with no evidence of disease for ≥5 years.
- Patients with myelodysplastic syndrome/acute myeloid leukaemia.
- Previous history of allogeneic marrow transplant.
- Evidence of distant metastasis apparent prior to randomisation.
- Patients with uncontrolled seizures. Pre-existing sensory or motor neuropathy of CTCAE v4.03, grade ≥2, Please see appendix 10 for possible exceptions in cases of severe pre-existing disability.
- Concomitant use of known potent CYP3A4 inhibitors and inducers. See section 11.11 and consider wash-out periods.
- Pregnant or breast-feeding women.
- Not suitable for neoadjuvant chemotherapy in the opinion of the responsible clinician.
- Major surgery within 14 days prior to starting trial treatment and patients must have recovered from any effects of any major surgery.
- Any evidence of other disease or any concomitant medical or psychiatric problems which in the opinion of the Investigator would prevent completion of treatment or follow-up. For example:
    - Evidence of severe or uncontrolled cardiac disease
    - Uncontrolled ventricular arrhythmia
    - Recent myocardial infarction (within 12 months)
    - Active infection including Hepatitis B, Hepatitis C and Human Immunodeficiency virus (HIV). Screening for chronic conditions is not required
- Patients unable to swallow orally administered medication and patients with gastrointestinal disorders likely to interfere with absorption of the trial medication. This

includes but is not limited to refractory nausea and vomiting, chronic gastrointestinal diseases or previous significant bowel resection.

- Known hypersensitivity to Olaparib, Carboplatin, Paclitaxelor their excipients (including cremophor).
- Whole blood transfusions in the last 120 days prior to blood sampling for BRCA test as it may interfere with the results (packed red blood cells and platelet transfusions are acceptable).

[redacted]

[redacted]

- [redacted]
- [redacted]
- [redacted]
- [redacted]
- [redacted]
- [redacted]
- [redacted]
- [redacted]

[redacted]

- [redacted]

[redacted]

- [redacted]
- [redacted]
- [redacted]
- [redacted]
- [redacted]
- [redacted]
- [redacted]
- [redacted]
- [redacted]
- [redacted]
- [redacted]
  - [redacted]
  - [redacted]
  - [redacted]
  - [redacted]
  - [redacted]
  - [redacted]
- [redacted]
- [redacted]

- [redacted]
- [redacted]

[redacted]

[redacted]	[redacted]		[redacted]
[redacted]	[redacted]	[redacted]	[redacted]
[redacted]	[redacted]	[redacted]	[redacted]
[redacted]	[redacted]	[redacted]	[redacted]

- [redacted]

10.4 Registration & Central testing prior to Randomisation

Please refer to the Patient Registration and Randomisation Guidelines for further details.

Participating sites are required to register the patient with the PARTNER Office prior to randomisation in order for the PARTNER Office to arrange central testing of gBRCA status and biomarkers scoring.

The recommended time window for the main trial is 6 weeks between diagnosis and treatment, (date of diagnosis is defined as the date of the tumour biopsy). **It is therefore essential, that the screening period starts as soon as possible after diagnosis for the BRCA gene and the biomarkers status to be determined as early as possible.**

Initial phase of the trial
During the initial phase of the trial, all TNBC patients will be tested for TILs, TNBC basal phenotype and BRCA status.
In the initial phase, TNBC patients can be randomised without their BRCA status being known. The result of the biomarkers CK5/6 and/or EGFR and AR if appropriate, is mandatory at randomisation unless the patient is known to be BRCA mutated. In this case, the result can be obtained at a later date. For all patients, the result of the TILs scoring is mandatory at randomisation as it is a stratification factor.

Post-closure of the TNBC non-BRCA recruitment phase
At this stage, the BRCA status is mandatory at randomisation for entry into the PARTNER trial and only patients positive for BRCA mutation are eligible. This means that if the BRCA status of the patient is not known at diagnosis, the patient must be approached immediately to give written informed consent (PARTNER pre-screening consent) for a sample for BRCA genetic testing. The result of the TILs scoring is also mandatory at randomisation in order to stratify patients. However, the results of the other biomarkers: CK5/6 and/or EGFR and AR if applicable, can be obtained at a later stage.

[redacted]

[redacted]

[redacted]

[redacted]

[redacted]

10.4.1 gBRCA test

Patients who meet the NHS Genomic Test Directory Criteria for BRCA1 and BRCA2 genes mutations (see appendix 7) may be tested within the trial.
To be clear, patients who do not meet the NHS Genomic Test Directory Criteria but presenting with the additional criteria listed in appendix 7 (Strong family history of breast, ovarian, pancreatic and/or high grade/metastatic prostate cancercancer/ ER positive breast cancer patients aged under 60 with a strong family history) will be tested within the trial and the cost of the testing will be covered by the trial.
Patients with a known BRCA mutation will not be re-tested but a de-identified copy of the test report will be required at randomisation.

[redacted]

Patients should ideally be tested using a blood sample however, alternative testing (e.g. from saliva) is acceptable if this is a standard & validated local gBRCA testing pathway and the results are produced in an official NHS clinical report. The blood sample for BRCA testing (minimum 5ml of blood in an EDTA tube) is to be sent to the approved PARTNER BRCA laboratories for testing. The blood sample for BRCA testing must only be taken after the patient has consented to give the sample through the pre-screening consent form.

[redacted]

[redacted]

[redacted]

It is the responsibility of the investigator randomising the patient to ensure that any patients who are found to carry a germline BRCA mutation are counselled by a local Geneticist as per local practice.

10.4.2 Tumour Infiltrating Lymphocytes (TILs) score

Pre-clinical models suggest that myeloid lineage leucocytes may be involved in shaping the microenvironment that either promotes or prevents tumour development. In addition there is evidence to support the concept that adaptive immunity mediated by B and T lymphocyte cells provides the foundation for a sustained anti-tumour response. For example in breast cancer extensive tumour infiltration by CD8+ T-cells is strongly associated with survival.

The hypothesis that pCR rates would be greater in patients with increased TILs in the pre-treatment core biopsy, has been investigated in a previous neoadjuvant study [76]. This study found that immunological factors were highly significant predictors of therapy response in trial in patients treated with Carboplatin.

The working group recommendations [77] refer to scoring TILs as a continuous variable (or at least 10% increments) and do not endorse a cut-off as there is insufficient data. Using similar criteria to currently published TILs assessment in neoadjuvant trials [20,76], in PARTNER, a percentage TILs score will be provided by a central pathologist and each registered patient will be assigned as having Lymphocyte Predominant Breast Cancer (LPBC) or not prior to randomisation. LPBC is defined as ≥60% stromal TILs.

TILs score defined as the proportion of lymphocytes as a percentage of stromal area excluding tumour cells. MUST be known at randomisation (<u>initial AND post-closure of the TNBC non-BRCA recruitment phase).</u>

Once the patient has given written informed consent through the pre-screening consent, sites will send one slide (Haematoxylin and Eosin (H&E)) of the patient's tumour taken from their diagnostic biopsy for TILs scoring (see Laboratory manual).

[redacted]

[redacted]

[redacted]

[redacted]

[redacted]

10.4.3 Assessment of the TNBC Basal phenotype

TNBC has been described as a heterogeneous group of cancer. Lehman [6] has classified them as BRCA, Basal Like, Immunomodulatary, Mesenchymal, Mesenchymal-Stem Like and Luminal Androgen Receptor. This study also showed that these different TNBC phenotypes respond differently to treatments. For the purpose of this trial, we have used an IHC classification of the basal TNBC phenotype as defined according to Blows [78] as followed:

- ER, HER2 negative
- CK5/6 or EGFR positive

Please note that patients who are not TNBC but eligible (i.e. gBRCA positive), do not need to be assessed for TNBC Basal phenotype prior to randomisation.

In order to define the TNBC phenotype as Basal, tumours will be centrally tested as follows:
- CK5/6: If the result is positive, the phenotype is considered TNBC-Basal and no additional assessments are required.
- CK5/6 negative tumours will be tested for EGFR expression. If EGFR is positive, the phenotype is considered TNBC-Basal and no additional assessments are required.

- Tumours which are both **CK5/6 negative and EGFR negative, i.e. not a TNBC basal** will be further tested for Androgen Receptor positivity. If the tumour is found to be CK5/6 negative, EGFR negative, **and strongly positive for AR, patients will be excluded from the trial** as there is insufficient evidence to suggest a benefit from Carboplatin in these TNBCs [79].

AR strongly positive is defined as more than 30% of the tumour cells over-expressing AR [79]. Please note that all patients will be tested for all markers (CK5/6, EGFR and AR) but not necessarily prior to randomisation if gBRCA.

Once the patient has given written informed consent through the pre-screening consent or the main PARTNER consent, the site will send **five unstained slides** of the tumour taken from the diagnostic biopsy for assessment of the TNBC basal phenotype (see Laboratory manual). Alternatively, a diagnosis tumour block can be sent which will be cut to obtain the 5 slides and then returned.

For UK participating sites, assessment of the TNBC basal phenotype will be undertaken by the Pathology Department of Cambridge University Hospitals NHS Foundation Trust. Results will be provided after a maximum of 2 weeks of the pathology slides being received.

For Overseas centres, see details in the Laboratory manual.

Queries on the progress of the testing can be made via the PARTNER office.

10.4.4 Registration and pre-screening

ER positive breast cancer patients <60, with a strong family history OR patients with a strong family history of breast, ovarian, pancreatic and/or high grade/metastatic prostate cancer can be registered and access the fast track BRCA testing.
For more information about the fast track BRCA testing please see Appendix 7.

The blood sample for BRCA testing must only be taken after patient has consented to give the sample through the pre-screening consent.

BRCA test, TILs and TNBC basal phenotype assessment tests (CK5/6 +/- EGFR +/- AR) as applicable, will only be carried out after receipt of a Patient Details and Registration Form.

All patients must be registered with the PARTNER office for central testing prior to randomisation.

To register a patient, please complete the CRFs 1 and 2SC (Patient Details and Registration and Screening Consent Forms) and email it to the coordination team at the PARTNER Office:

Email: cuh.partner@nhs.net

For any assistance, please telephone the PARTNER OFFICE on:

Tel number: 01223 348 447

Please refer to the Patient Registration and Randomisation Guidelines for further details.

[redacted]

[redacted]

10.5 Screening

Trial specific assessments will only be carried out after patient has given written informed consent. Screening procedures:

- ☐ BRCA status if required.
- ☐ Tumour Infiltrating Lymphocytes score (tested centrally).
- ☐ TNBC phenotype status (CK5/6 +/- EGFR) and if appropriate Androgen receptor status by IHC for non-brca patients. The TNBC phenotype of confirmed BRCA-positive patients can be done at any time (tested centrally).
- ☐ ER, PR and HER2, status (all 3 tested locally, as per local practice). An Allred score for ER and PR of 3 is considered negative for the PARTNER trial, however if local site practice is to treat patients with an Allred score of 3 with adjuvant hormones, then this is permitted in the trial.
- ☐ Medical history, specifically the presence or absence of diabetes, and other co-morbidities associated with peripheral neuropathy must be documented.
- ☐ Family medical history.
- ☐ Physical examination:
    - Vital signs: Pulse, blood pressure (if they are within normal ranges, please record that they have been checked in source data)
    - Height and weight
    - ECOG Performance Status
- ☐ A **serum** pregnancy test for women of child-bearing age is required after pre-screening consent has been given. This must be documented as negative and the patient must be advised to use contraception thereafter to avoid pregnancy whilst on treatment.
  Note: Patients who have received fertility hormone injection for eggs harvest can present with a positive serum pregnancy test. If there is no clinical doubt for the treating clinician and the patient, that the patient is not pregnant, the patient can be randomised (after contacting the PARTNER office) but the patient will need to show a negative serum pregnancy test before cycle 2. All discussions with patient regarding risks and benefits of proceeding with trial need to be clearly documented.
- ☐ Baseline tumour imaging (US or MRI): it is strongly advised to repeat as close to cycle 1 as possible, if there is significant clinical change in the tumour mass clinically and if previous radiology measurements have been performed more than 6 weeks prior to randomisation.
- ☐ It is advised to assess clinically enlarged / abnormal axillary nodes by FNA (Fine Needle Aspiration) or core biopsy to define involvement. This is mandated for patients with locally advanced disease.
- ☐ Full blood count (recommended timing is within 7 days prior to cycle 1), as per local practice.
- ☐ Biochemical screen (recommended timing is within 7 days prior to cycle 1), as per local practice.
- ☐ Obtain pre-treatment blood sample (see section 13.1).

- ☐ Patients with **tumours ≥ 5 cms** on ultrasound or MRI

  and/or

  locally advanced or inflammatory disease (definition in section 10.1),

  and/or

  clinically involved axillary nodes,

  have an identifiable risk of metastatic disease and therefore **MUST be staged** in accordance with local practices for early breast cancer.

The results of the relevant tests must be made available to local site investigators prior to randomisation. If staging investigations show metastatic disease, the patient is not eligible for the trial, and must proceed with standard management as per local practice.

[redacted]

10.6 Treatment Assignment

PARTNER Randomisation

10.6.1 Method of Randomisation

Eligible patients will be randomly assigned to either the control arm (chemotherapy alone) or one of the two research arms (chemotherapy with Olaparib at different timings) using minimisation method in a 1:1:1 ratio in Stage 1 and Stage 2. In Stage 3, patients will be randomly assigned with a 1:1 ratio to either control or the selected research arm.

Stratification variables are defined as follows:

Cancer type: TNBC (non-BRCA) / BRCA1 / BRCA2 / unknown; the unknown category is only applicable prior to the completion of TNBC non-brca recruitment phase (see Section 10.6.2)

Tumour size: ≤50mm; >50 mm. In cases with multi-focal disease or bilateral disease, the size of the largest target tumour will be used (ER positive tumours could be used as target lesion providing the patient is a BRCA carrier). For the purpose of this trial, inflammatory tumours would be considered as >50 mm at randomisation.

Histopathological involvement of axillary nodes: no; yes

NB: The TMG strongly recommends diagnosing the involvement of axillary nodes by Fine Needle Aspiration (FNA) or core biopsy. They do not recommend a Sentinel Lymph Node (SLN) biopsy pre-treatment.

Tumour Infiltrating Lymphocytes: <60%; ≥60%. See section 10.4.2.

10.6.2 Procedure of Randomisation

Patients must be registered for the trial at the start of their screening and prior to randomisation.

Initial phase of the trial

The result of all the biomarkers (CK5/6 and/or EGFR and AR if applicable) is mandatory at randomisation unless the patient is BRCA mutated.
It is acceptable to randomise patients without their BRCA status available however, the BRCA test must have been requested.
The TIL score is mandatory prior randomisation for all patients.

Post-closure of the TNBC non-BRCA recruitment phase

At this stage, the BRCA status is mandatory at randomisation and only patients positive for a BRCA mutation are eligible.
The result of the TILs scoring is also mandatory at randomisation to stratify patients but the results of CK5/6 and/or EGFR and AR if applicable, can be obtained at a later stage.

Start of treatment is strongly recommended within 6 weeks of the diagnostic biopsy and registration and randomisation must be complete prior to the start of trial treatment. In uncommon circumstances, where medically acceptable, treatment is permitted to start within a maximum of 9 weeks of the diagnostic biopsy.

Data will be entered into a web-based central randomisation system by the relevant participating site staff.

At the site initiation, the trial coordinator will train site staff in how to access and use the randomisation system.
The web-based central randomisation system will allocate patient trial numbers sequentially in the order in which the patients are randomised.

Note: The name of the Investigator directly responsible for the patient's care will be requested at registration and at randomisation. Investigators must be registered with the PARTNER office before they are permitted to register or randomise patients into the trial.

Eligibility Criteria CRF must be completed prior to randomisation, signed by the Investigator, and sent to the PARTNER office. When the PARTNER office authorises randomisation, the patient's details can then be entered on the randomisation system.

The following data will be required in order to randomise a patient:

- Screening ID (given at registration)
- Patient's date of birth
- Patient's initials
- gBRCA status
- Tumour size
- Histopathological confirmation of involvement of axillary nodes mandated for patients with locally advanced disease
- Tumour Infiltrating Lymphocytes score (see section 10.4.2)
- Confirmation that the randomising Investigator has signed the Eligibility Form.
- Confirmation that the PARTNER office has authorised randomisation
- A unique trial number and the treatment arm will be assigned by the randomisation system, which should be transcribed onto the patient's original Randomisation Form.

[redacted]

[redacted]

[redacted]

[redacted]

[redacted]

[redacted]

[redacted]

10.7 Discontinuation of trial treatment and patient withdrawal

Discontinuation of trial treatment or withdrawal of consent by patients for any reason should be communicated to the PARTNER office as soon as possible by telephone or email.

10.7.1 Discontinuation of trial treatment

Patients should **discontinue trial treatment** in the following circumstances and this should be reported on the Treatment discontinuation / Withdrawal CRF:

- If the Investigator decides that the patient should be discontinued from the treatment at any point due to toxicity or due to an inter-current illness.
- If the patient within 4 weeks has not recovered from PARTNER main treatment-related toxicity to an extent that allows further treatment.
- If the patient within 14 days has not recovered from *PartnerING* pathway treatment-related toxicity to an extent that allows further treatment.
- If body weight falls to ≤30kg for *PartnerING* patients.
- If the patient opts to discontinue the treatment or chooses not to comply with trial procedures.
- If the patient's disease is progressing while on trial treatment.
- If the patient becomes pregnant during trial treatment.
- If the patient is unable to comply with trial requirements.
- If the patient is found ineligible.

If MDS and/or AML are confirmed while on treatment with Olaparib, it is recommended that Olaparib should be discontinued and the patient be treated appropriately.

Patients who discontinue trial treatment will be followed up as per schedule for safety reasons until resolution of any IMP-related toxicity, or outcome of pregnancy as appropriate.

If any patient becomes pregnant during the trial, ALL trial treatments must be discontinued and the patient must be followed up until birth or termination of pregnancy.
In the event that the PARTNER patient is a male and his PARTNER becomes pregnant during the course of the trial, the pregnancy must be followed up until birth or termination.

With on-going consent, patients should remain in the trial and in follow-up according to the Protocol visit schedules, for the Intention-To-Treat analysis. Data collection, blood and tissue samples should continue to be collected as per Protocol.

Please note: Patients who proceed to the anthracycline section of treatment earlier than cycle 5 (for example due to disease progression – see section 13.11), should remain on trial with the completion of all Protocol-specific Case Report Forms (CRF) (e.g. Treatment Forms, Radiology Forms, Surgery Form) along with collection of all sub-study samples or questionnaires that the patient has consented to, e.g. fresh tissue and/or blood samples, and/or Quality of Life Questionnaires.

10.7.2 Withdrawal of consent

Patients may withdraw their consent to participate in the trial at any time. If the patient explicitly states that they wish to withdraw from receiving any further protocolised treatment, the Investigator should inform the PARTNER office immediately and complete the Treatment discontinuation / Withdrawal Form. If the patient additionally withdraws consent for any further data or samples to be collected, then no further data can be collected during long term follow-up and no further samples can be collected.
However, data up to the time of consent withdrawal will be included in the data reported for the trial.

11 Trial Treatments

For the purpose of this trial, Olaparib, Paclitaxel, Carboplatin, AZD6738, and Durvalumab will be considered as Investigational Medicinal Products (IMPs) conducted with a Clinical Trial Authorisation (CTA) in the U.K and other appropriate country specific trial authorisations.

11.1 Treatment Summary

G-CSF must be given as primary prophylaxis with the administration of the Paclitaxel& Carboplatin(+/- Olaparib).

Patients will be randomised to receive one of the following treatments:

- **Standard (Control) Arm**:

Paclitaxel I.V, 80mg/m^2 on Day1, 8 & 15 every 3 weeks for 4 cycles and

Carboplatin I.V, AUC 5 on Day1 of a 21 day cycle for 4 cycles.

Supportive medication (mandated): G-CSF as per local practice, see below for recommendation.

All patients will receive anti-emetic and hypersensitivity prophylaxis to cover each cycle of chemotherapy as per local practice.

Followed by 3 cycles of standard local anthracycline-based chemotherapy (mandated).

- **Research Arm 1:**

Olaparib oral, 150mg b.d. on **Day-2 to D10** every cycle for 4 cycles.

Paclitaxel I.V, 80mg/m^2 on Day1, 8 & 15 every cycle for 4 cycles and

Carboplatin I.V, AUC 5 on Day1 every cycle for 4 cycles

Supportive medication (mandated): G-CSF as per local practice, see below.

All patients will receive anti-emetic and hypersensitivity prophylaxis to cover each cycle of chemotherapy as per local practice.

Followed by 3 cycles of standard local anthracycline-based chemotherapy (mandated).

- **Research Arm 2:**

Olaparib oral, 150mg b.d on **Day3 to Day14** every cycle for 4 cycles.

Paclitaxel I.V, 80mg/m^2 on Day1, 8 & 15 every cycle for 4 cycles and

Carboplatin I.V, AUC 5. on Day1 every cycle for 4 cycles

Supportive medication (mandated): G-CSF as per local practice, see below.

All patients will receive anti-emetic and hypersensitivity prophylaxis to cover each cycle of chemotherapy as per local practice.

Followed by 3 cycles of standard local anthracycline-based chemotherapy (mandated).

<u>Note:</u> G-CSF must be given as primary prophylaxis with the administration of the Paclitaxel & Carboplatin +/- Olaparib), <u>unless</u> there are any contra-indications, or due to cessation for clinical reasons in the judgement of the clinician caring for the patient.

G-CSF can be given as per local practice.

The recommended filgastrim schedule/dose based on patients' weight is:

G-CSF ≤ 70kg 300mcg sc ONCE a day on Days 3-5, Days 10-12 and Days 17-19.
G-CSF > 70kg 480mcg sc ONCE a day on Days 3-5, Days 10-12 and Days 17-19.

Table 3: Summary of Treatment Schedule

[redacted]

If the Carboplatin due on D1 is delayed, all three drugs are delayed, see section 12.3. Therefore, blood tests must be done prior starting Olaparib treatment.

For Research arm 1, we recommend performing the pre-cycle blood test on or before D-2. Once the blood is confirmed to be satisfactory, the patient should take the first tablet of that day in the morning, in clinic or at home.

Paclitaxel is not to be delayed on its own but can be omitted (D1, D8 or D15). In such case, the Carboplatin or the Olaparib is prescribed and administered as planned, see section 12.3. NB: Paclitaxel given +/-2days in the exceptional circumstance of planned treatment falling on a Bank Holiday (resulting in closure or reduction of patient services), will be recorded on the CRF but will not be documented as a protocol non-compliance.

Olaparib is not to be delayed on its own but can be dose reduced (see section 12.4) or omitted.

[redacted]

[redacted]

[redacted]

[redacted]

[redacted]

[redacted]

11.2 Maximum duration of treatment of a patient

The trial employs 21 to 24 weeks of neoadjuvant chemotherapy (depending on the local anthracycline regimen), unless there is evidence of disease progression, in which case patients will proceed either to the anthracycline phase of chemotherapy or to surgery (decision to be made by treating clinician). For further details, refer to section 13.11.

[redacted]

11.3 Procedures for monitoring treatment compliance

Administration of all trial drugs will be recorded in the appropriate sections of the Carboplatin and Olaparib, Paclitaxel and Anthracycline-based chemotherapy forms. Details and reasons for dose reductions and delays will also be recorded.

To monitor compliance, the patient will complete a diary on the intake of Olaparib which will be brought to the site and checked at each visit.

Patients should return all unused oral medication and empty containers to the Investigator.

[redacted]

[redacted]

[redacted]

11.4 Supply, accountability and dispensing

11.4.1 Pharmacy responsibilities

All pharmacy aspects of the trial at the participating sites are the responsibility of the Principal Investigator (PI) who will delegate this responsibility to the local pharmacist, or other appropriately qualified personnel. This delegation of duties must be recorded on the Site Staff Delegation Log. The PI or a delegated individual, e.g. the trial pharmacist, must

ensure that the trial medications are stored and dispensed in accordance with local practice, applicable regulatory requirements and trial-specific prescriptions.

Olaparib tablets, [redacted]supplied for PARTNER/[redacted] trial should only be used for patients within the trial and not be used outside the context of this Protocol.

11.4.2 Drug Accountability

The site pharmacy must maintain accountability records for all of the IMPs which may include receipt, dispensing, returned medication, storage conditions and destruction of returned/unused medication. Template accountability forms will be supplied however, sites are permitted to use their own drug accountability records as long as the same information is recorded and is available to the Sponsor. Copies of completed drug accountability logs must be submitted for all trial patients upon request from the PARTNER office for monitoring purposes.

Following the termination of the trial and at the request of the Sponsor, all unused IMPs will be accounted for and destroyed locally at the trial sites in accordance with local practice. See the latest version of the pharmacy manual for details.

11.5 Olaparib

11.5.1 Legal Status

Olaparib capsules and tablets are both currently licensed within the EU for treatment of some cancers. However, only the supplied Olaparib tablets can be used for the PARTNER trial Olaparib tablets cannot be substituted for Olaparib capsules due to differences in dosing and bioavailability of each formulation.
Olaparib supplied should only be used by the named Investigators, for the patients specified in this Protocol, within the PARTNER trial.

11.5.2 Description

Olaparib is a poly-ADP-ribose polymerase (PARP) enzyme inhibitor. PARP inhibitor selectively kills tumour cells with an impaired homologous recombination DNA repair pathway whilst sparing normal cells.

11.5.3 Supply

Olaparib will be supplied free of charge by AstraZeneca Ltd. In the E.U. Olaparib will be labelled, and distributed by Mawdsley-Brooks & Co Ltd in accordance with local regulations and Good Manufacturing Practice. Olaparib will be supplied as 150mg, 100mg and 25mg tablets.
Olaparib will be distributed directly to sites following confirmation that all necessary country specific regulatory and ethical approvals are in place.
Further details on the ordering of supplies are provided in the pharmacy manual.

11.5.4 Labelling

Olaparib will be supplied in the E.U. and labelled according to EudraLex Volume 4: Annex 13 Investigational Medicinal Products of the EU guide to Good Manufacturing Practice (GMP). An example of the label can be found in the pharmacy Manual.

11.5.5 Storage Conditions

Olaparib must be stored in its original bottle with the cap on tightly, in a secure, limited access storage area.
Olaparib must be stored at room temperature below 30°C. Refer to the Olaparib IB and the PARTNER pharmacy manual.
Maintenance of a temperature log (manual or automated) is required and any temperature deviations must be reported to the PARTNER office immediately as detailed in the Pharmacy manual.

11.5.6 Returns and Destruction

All previously dispensed Olaparib tablets that are returned to the Investigator, should be destroyed once they are counted and recorded in the relevant accountability forms. Refer to the pharmacy manual.

11.5.7 Dose

Patients will self-administer Olaparib by mouth.

Olaparib dose is 150mg twice a day orally either on Day -2 to Day 10 or on Day 3 to Day 14, every 3 weeks, for 4 cycles. Olaparib will be given alongside Paclitaxel and Carboplatin during the first 4 cycles of PARTNER treatment. Concomitant administration of Olaparib with anthracycline-based chemotherapy is not included within this trial.

11.5.8 Administration

Olaparib tablets should be taken at the same time each day approximately 12 hours apart. Olaparib tablets should be swallowed whole and not chewed, crushed, dissolved or divided.

If vomiting occurs shortly after the Olaparib tablets are swallowed, the dose should only be replaced if all of the intact tablets can be seen and counted. Should any patient miss a scheduled dose for whatever reason (e.g. as a result of forgetting to take the tablets), the patient will be allowed to take the scheduled dose up to a maximum of two hours after that scheduled dose time. If greater than two hours after the scheduled dose time, the missed dose is not to be taken and the patient should take their next dose at the next scheduled time.

Patients should be advised that the consumption of **grapefruit and Seville oranges** (as well as other products containing these fruits e.g. grapefruit juice or marmalade) are prohibited for all patients (regardless of trial Arm) during trial treatment.

Because the AEs related to Olaparib may include asthenia, fatigue and dizziness, patients should be advised to use caution while driving or using machinery if these symptoms occur.

11.5.9 Known drug reactions

Olaparib is generally well tolerated. Adverse drug reactions (ADRs) are usually mild or moderate without treatment discontinuation.
The most common ADRs are:

- Blood and lymphatic system toxicities: Anaemia, Neutropenia, Leukopenia
- Metabolism and nutrition toxicities: Decreased appetite
- Nervous system toxicities: Dizziness, Headache, Dysgeusia
- Respiratory toxicities: Cough, Dyspnoea

- Gastrointestinal toxicities: Vomiting, Diarrhoea, Nausea, Dyspepsia, Constipation, Abdominal pain
- General toxicities: Fatigue, Arthralgia, Asthenia

The full listing of ADRs can be found in the latest MHRA approved IB present in the Investigator Site File.

More detailed expected toxicities associated with Olaparib which will assist the Investigator in the classification of expectedness and relatedness of serious adverse reactions are given in the latest MHRA approved Reference Safety Information present in the Investigator Site File.

For interactions with other drugs, refer to section 11.11 of the Protocol.

11.6 Paclitaxel

11.6.1 Legal Status

The IMP Paclitaxel is licensed within the UK as an anti-cancer cytotoxic agent.

11.6.2 Supply

Paclitaxel is available commercially and will be supplied from local hospital stock.
There is no requirement to ring-fence off the shelf general hospital supplies of this IMP.

11.6.3 Labelling

Paclitaxel will be labelled according to EudraLex Volume 4: Annex 13 Investigational Medicinal Products of the EU guide to Good Manufacturing Practice (GMP). An example of the label can be found in the pharmacy Manual.

11.6.4 Storage Conditions

Paclitaxel used in this trial should be stored, handled and dispensed as detailed in the current SmPC of the brand being used as per local practice.

11.6.5 Dose

The preparation, storage and administration of diluted Paclitaxel should be carried out using non-PVC-containing equipment as per SmPC.

Paclitaxel I.V. 80mg/m^2 in 0.9% sodium chloride 500ml or according to local practice, over 60 minutes on days 1, 8 & 15, every 3 weeks for 4 cycles.

Dose will be calculated according to Body Surface Area (BSA) using the Dubois Formulae.

BSA and dose should only be recalculated if the patient’s body weight changes by more than 10%.

Dose banding and dose capping according to local practice is acceptable.

11.6.6 Administration

All patients must be pre-medicated with corticosteroids, antihistamines, and H2 antagonists prior to Paclitaxel treatment as per local practice.

Table 6: Recommended pre-medication for Paclitaxel

Drug	Dose	Administration prior to Paclitaxel
Dexamethasone	8mg I.V.	30 minutes prior to Paclitaxel.
Chlorphenamine	10mg I.V	Push diluted with 5-10ml Normal Saline.
Ranitidine	50mg I.V	In 20ml Normal Saline over 2 minutes.

If patients are unable to tolerate weekly dexamethasone at this dose and have not experienced Paclitaxel hypersensitivity, the dose can be gradually reduced at the Investigator's discretion. If hypersensitivity reaction then develops, dexamethasone should be reintroduced at a minimum of 8mg I.V prior to ALL subsequent Paclitaxel infusions.

Also give anti-emetics immediately pre-chemotherapy as per local practice; this may include oral or intravenous 5HT3 antagonists.

Paclitaxel should be reconstituted and administered according to the most recent SmPC for the brand being used.

Ensure that Paclitaxel is given prior to Carboplatin.

Paclitaxel should be administered through an in-line filter with a microporous membrane ≤ 0.22 µm.

Paclitaxel is a vesicant, in case of extravasation, refer to local practice guideline.

Anaphylaxis treatment should be available during infusion for the emergency treatment of hypersensitivity reactions.
Monitor the patient closely for allergic reactions and cardiac arrhythmias as per local practice.

Hypersensitivity reactions with weekly Paclitaxel have a low incidence of 1-1.5% [80]]. They should be managed according to local practice. See section 12.3.
If it becomes necessary to discontinue Paclitaxel because of toxic effects then patients can continue treatment with single agent Carboplatin. **Substitution of Paclitaxel with Docetaxel or nab-Paclitaxel is not allowed within this trial.**

11.6.7 Known drug reactions & interaction with other therapies

Expected toxicities associated with Paclitaxel which will assist Investigators in the classification of expectedness and relatedness of serious adverse events is given in the latest MHRA approved Reference Safety Information (RSI).
Please note that as Paclitaxel contains ethanol the co-administration with medicines containing e.g., propylene glycol or ethanol may lead to accumulation of ethanol and induce adverse effects, in patients with low or immature metabolic capacity. The effects of ethanol may be reduced because this medicinal product is normally administered over a period of 3 hours.

Interactions are also detailed in the Paclitaxel SmPC but please note that the preparation, storage and administration of diluted Paclitaxel should be carried out using non-PVC-containing equipment.

11.7 Carboplatin

11.7.1 Legal Status

The IMP Carboplatin is licensed within the UK as an anti-cancer cytotoxic agent.

11.7.2 Supply

Carboplatin is available commercially and will be supplied from local hospital stock.
There is no requirement to ring-fence off the shelf general hospital supplies of this IMP.

11.7.3 Label

Carboplatin will be labelled according to EudraLex Volume 4: Annex 13 Investigational Medicinal Products of the EU guide to Good Manufacturing Practice (GMP). An example of the label can be found in the pharmacy Manual.

11.7.4 Storage Conditions

Carboplatin used in this trial should be stored, handled and dispensed as detailed in the current SmPC of the brand being used.

11.7.5 Carboplatin dose

Aluminium containing equipment should not be used during preparation and administration of Carboplatin.

Carboplatin I.V. AUC5 in 5% dextrose 500ml or according to local practice, over 30-60 minutes on Day 1 every 3 weeks for 4 cycles.

As you are aware, there are GFR limitations, the formulae used to calculate the creatinine clearance are inaccurate at the extremes of age and weight. The calculated GFR may be falsely high in obese young women and falsely low in thin women.
In order to ensure that patients received the accurate dose of Carboplatin and do not suffer from toxicities due to an overdose of Carboplatin, **it is strongly recommended that GFR (Glomerular Filtration Rate) is measured using an EDTA GFR** (nuclear medicine) in particular for BRCA patients and for patients who are significantly over or under weight or have pre-existing renal problems.
It is mandatory for sites to use EDTA GFR if this test is available on site. For sites which do not perform EDTA GFR as standard, these sites are not required to do so. Sites can use EDTA or equivalent nuclear medicine assessment of renal function as per their local practice.

Please note that the EDTA GFR value should be used in the dose calculations uncorrected for BSA.

If this is not standard practice, please contact the PARTNER office.

Carboplatin dose is calculated using the Calvert formula [81]:

Carboplatin dose = Target AUC x (GFR + 25)

The exact dose of Carboplatin therefore depends on the GFR and the method of calculating GFR will also affect the Carboplatin dose.
For the purpose of this Protocol the GFR is considered equivalent to the creatinine clearance.

If a calculated creatinine clearance is used to calculate the Carboplatin dose, then this should be done using the Cockcroft-Gault formula.

If the calculated **serum creatinine clearance is <60ml/minute,** then a formal measurement of the GFR is **mandatory by an EDTA GFR** (Sites unable to perform EDTA GFR as standard must use equivalent nuclear medicine assessment of renal function as per their local practice)**.**

For sites unable to perform EDTA GFR, please contact the PARTNER office.

EDTA GFR (or equivalent) is unlikely to be available before the first cycle, but is **strongly recommended** for dosing of the second cycle after which the dose will be '**fixed**' for the subsequent Carboplatin cycles [82], unless it is reduced for reasons of toxicity (most commonly myelosuppression). See Appendix 8 for further details.

In terms of the maximum dose of Carboplatin, we recommend following the FDA guidance on Carboplatin dosing based on a GFR estimate that is capped at 125 mL/min for patients with normal renal function. No higher estimated GFR values should be used.

For a target AUC = 5, the maximum dose is 5 x 150 = 750 mg

For a target AUC = 4, the maximum dose is 4 x 150 = 600 mg (where dose banding applied: 630 mg) Requirements for re-assessment of the Glomerular Filtration Rate (GFR) during chemotherapy
The GFR should be recalculated, or re-measured, due to:

- Renal toxicity (serum creatinine >1.5 x ULN, CTCAE v4.03 Grade 2 or above).
- Serum creatinine changes of ≥10% compared to baseline, or last creatinine value used to calculate Carboplatin dose (whichever is most recent).
- Cycle 2, if there has been significant doubt about the true GFR at cycle 1 (according to clinical judgement).

Routine recalculation of the Carboplatin dose at the start of each cycle is not expected unless the above conditions are met. Dose capping of Carboplatin may be carried out according to local practice.

11.7.6 Administration

Carboplatin should be reconstituted and administered according to the most recent SmPC for the brand being used as per local policy.

Give anti-emetics as per local practice; this may include oral or intravenous 5HT3 antagonists.

Ensure that Paclitaxel is given prior to Carboplatin.

Carboplatin is an irritant, in case of extravasation, refer to local practice guidelines.

Monitor the patient closely for allergic reactions and cardiac arrhythmias as per local practice.

Hypersensitivity reactions should be managed according to local practice.
See section 12.3 for advice on hypersensitivity management. In the event of severe and persistent myelosuppression, Carboplatin should be discontinued to avoid infectious complications including fatal outcomes.
If it becomes necessary to discontinue Carboplatin because of toxic effects then patients can continue treatment with single agent Paclitaxel. **Substitution of Carboplatin with Cisplatin is not allowed within this trial.**

11.7.7 Known drug reactions & interaction with other therapies

Expected toxicities associated with Carboplatin which will assist the Investigator in the classification of expectedness and relatedness of serious adverse reactions is given in the latest MHRA approved RSI. Interactions are also detailed in the Carboplatin SmPC but please note that Aluminium containing equipment should not be used during preparation and administration of Carboplatin.

[redacted]

[redacted]

[redacted]

[redacted]

[redacted]

[redacted]

[redacted]

[redacted]

[redacted]

[redacted]

[redacted]

[redacted]

[redacted]

[redacted]

[redacted]

[redacted]

[redacted]

[redacted]

[redacted]

[redacted]

[redacted]

[redacted]

[redacted]

[redacted]

[redacted]

[redacted]

[redacted]

[redacted]

[redacted]

[redacted]

[redacted]

11.10 Non-Investigational Medicinal Products

G-CSF and anthracyclines are classified as non-IMPs for this trial and are to be supplied by hospital stock as per local practice.

11.11 Concomitant therapy and washout Periods

11.11.1 Supportive therapies

Patients should receive full supportive care during and after the administration of chemotherapy in accordance with local practice.

This includes transfusion of blood and blood products. G-CSF is mandatory as prophylactic treatment in this trial. These treatment details should be recorded in the trial CRF.

Anti-emetics and antibiotics routinely administered as part of the chemotherapy regimen do not need to be recorded in the CRF.

Anaphylaxis monitoring should be undertaken during administration of Carboplatin and Paclitaxel as per local practice.

Treatment with concomitant, systemic or investigational anti-cancer agents are not allowed in this trial.

If medically feasible, patients taking regular medication, with the exception of potent inhibitors or inducers of CYP3A, should be maintained on it throughout the study period.

[redacted]

11.11.2 Restricted concomitant medications

Patients should avoid concomitant use of drugs and/or ingestion of foods known to modulate CYP3A4 enzyme activity from the time they enter the screening period until 28 days after the last dose of trial medication.

In vitro data have shown that the principal enzyme responsible for the formation of the three main metabolites of Olaparib is CYP3A4 and consequently, although the contribution of metabolic clearance to total drug clearance in man is currently unknown this restriction is required to ensure patient safety.

<u>Strong or moderate **CYP3A inhibitors or inducers should not be taken** with study treatment</u>.

Please refer to Appendix 12 for a list of drugs most often previously reported to be associated with clinically significant drug interactions. For subjects taking any of these drugs the required wash-out periods before starting Olaparib/AZD6738 is five half-lives; except for St. John's Wort, which is 3 weeks.

The following anti-epileptic medications **are allowed** to be taken throughout the trial:

- Gabapentin, Vigabatrin, Sodium Valproate*, Lamotrigine and Levetiracetam.

** Patients taking Sodium Valproate would require closed monitoring if included within PartnerING pathway.*

After randomisation if the use of any potent inducers or inhibitors of CYP3A4 are considered necessary for the patient's safety and welfare, the Investigator must inform the Chief

Investigator or designee. A decision to allow the patient to continue in the trial will be made on a case-by-case basis.

Herbal preparations are not allowed throughout the study. These herbal medications include but are not limited to: St. John's wort, kava, ephedra (ma hung), gingko biloba, dehydroepiandrosterone (DHEA), yohimbe, saw palmetto and ginseng.

[redacted]

[redacted]

[redacted]

[redacted]

[redacted]

[redacted]

[redacted]

[redacted]

11.11.4 Food & Drink

Patients are advised that consumption of **grapefruit and Seville oranges** (as well as other products containing these fruits e.g. grapefruit juice or marmalade) is prohibited for all patients (regardless of trial Arm) during trial treatment for PARTNER [redacted].

11.11.5 Vaccines

Live virus and bacterial vaccines should not be administered during Olaparib treatment and [redacted] and until 3 months post-treatment. An increased risk of infection by administration of live virus and bacterial vaccines has been observed with conventional chemotherapy drugs and the effects with Olaparib/[redacted] are unknown.
Note that the virus contained in the influenza vaccination is inactivated and therefore patients should be encouraged to receive it.

11.11.6 Anticoagulant Therapy

Patients who are taking Warfarin may participate in this trial. However, it is recommended that prothrombin time (International Normalised Ratio (INR)) and activated partial thromboplastin time be monitored carefully, at least once per week for the first month, then monthly if the INR is stable. Subcutaneous Heparin and novel oral anticoagulants such as Edoxaban are permitted.

11.11.7 Anti-emetics/Anti-diarrhoeals

If a patient develops nausea, vomiting and/or diarrhoea, then these symptoms should be reported as AEs or SAEs and appropriate standard treatment for the event given.

[redacted]

11.11.8 Herceptin® (trastuzumab)

Women with HER-2 positive disease are to be excluded from this trial as there are no data on the safety of this combination of drugs.

11.11.9 Blood components (For PartnerING patients only)

Patients should not donate blood or blood components while participating in this study and through 180 days after receipt of the final dose of Durvalumab combination therapy.

12 Management of toxicity

The decision whether to continue with trial medication should be based on the individual circumstance and the responsible clinician's judgment that continuation is in the patient's best interest.

Toxicities derived from Cycle 1 to 4 of PARTNER treatment are more likely to be related to chemotherapy than Olaparib. We therefore recommend in the first instance to treat the toxicity as a chemotherapy related toxicity and if the symptoms persist, to treat the toxicity as related to the Olaparib.

12.1 Maximum dose delay

The maximum permitted dose-delay for the chemotherapy (+/- Olaparib) is 4 weeks for recovery of severe toxicity or for unscheduled procedures (e.g. appendectomy). If longer delays are required, then the patient will be discontinued from trial treatment, and alternative therapy considered by the treating clinician.

[redacted]

12.2 G-CSF specific toxicity

G-CSF toxicities should be managed as per local practice.

12.3 Chemotherapy specific toxicity

During the safety stage of the trial (first 75 patients), CTCAE **Grade ≥ 3** which are not already reported as an SAE for example thrombocytopenia grade 3 (platelets= 36), must be reported as an AESI within 24 hours of becoming aware of the event.

Recommended dose reductions

Haematological Toxicity
FBC should be repeated at least weekly until haematological recovery has occurred.
If haematological recovery occurs beyond 7 days, it is recommended that doses of Carboplatin and Paclitaxel are modified to AUC 4 and 60mg/m^2 respectively, or AUC 3.5 and 45mg/m^2 respectively as a second step.

To reduce the risk of neutropenic complications, dose delays and to maintain dose-intensity, the trial will use primary prophylactic **G-CSF support**, see section 11.1.

A new cycle of Olaparib, Paclitaxel and Carboplatin can only proceed if all criteria below are fulfilled:

- ANC ≥ 1.0 x 10^9/l
- PLT count ≥ 75 x 10^9/l
- There is no complicating fever, infection or significant bleeding

If the Carboplatin is delayed, then all three drugs are to be delayed.

The administration of **D8 and D15** Paclitaxel can only proceed if all criteria below are fulfilled:

- ANC ≥ 0.5 x 10^9/l
- PLT count ≥ 50 x 10^9/l
- There is no complicating fever, infection or significant bleeding

If all these parameters are not met, it is recommended that the Paclitaxel dose should be omitted and the patient reviewed at the time of their next scheduled dose. No dose delays are allowed and therefore omitted doses of Paclitaxel will not be replaced.
If the Paclitaxel is omitted (D1, D8 or D15), the **Olaparib is prescribed as planned** in table 3 i.e. taken on either on D-2 to D10 (Research Arm 1) or on D3 to D14 (Research Arm 2).

If more than one Paclitaxel dose is omitted in the same cycle or at least one dose is omitted from two consecutive cycles, it is recommended to reduce the dose of both Carboplatin and Paclitaxel by one dose level (to AUC 4 and 60mg/m^2 or AUC 3.5 and 45 mg/m^2 as a second step).

Please note that no dose delay or reduction is recommended for **anaemia**. However, anaemia is likely to be a cumulative problem and we recommend transfusion if symptomatic and/or Hb level ≤80g/L (old scale 8g/dL) in order to maintain dose intensity.

Patients who fail to recover adequate ANC and PLT counts after a delay of 2 weeks or more, or who have consecutive dose limiting toxicities, are unlikely to be able to tolerate standard Carboplatin and Paclitaxel. With the support of the G-CSF, such cases are likely to be rare and should therefore be discussed with the PARTNER Office/Chief Investigator prior to patient withdrawal.

Non-haematological toxicity
For toxicity grade 3 or above, we strongly recommend to do an EDTA GFR measurement or to contact us if an EDTA GFR has not been done already.

Renal toxicity
There are no specific dose modifications for renal toxicity which is not expected. However, the GFR used to calculate the Carboplatin dose should be recalculated in the event of renal toxicity or changes in the serum creatinine outside the accepted criteria given in the trial Protocol.

Neuropathy
Grade 2 sensory or motor neuropathy (CTCAE v4.03) requires a dose reduction of the Paclitaxel treatment to 60mg/m^2. If grade ≥3 sensory or motor neuropathy occurs at the next cycle, the Paclitaxel must be interrupted until neuropathy has resolved to ≤ grade 2. If the recovery requires a delay of less than three weeks, Paclitaxel should be reintroduced but with a further dose reduced (45mg/m^2). If the recovery requires a delay of more than three weeks then the Paclitaxel should be omitted from subsequent cycles and treatment continued with single agent Carboplatin at the same AUC used in combination with Paclitaxel.

Mucositis
For mucositis grade ≥3 chemotherapy should be delayed until the mucositis has resolved to grade ≤1. Paclitaxel can be reduced (60mg/m^2 and 45mg/m^2 as a second step) in subsequent cycles at the discretion of the Investigator. If the mucositis recurs, or persists for more than three weeks, at grade ≥3 chemotherapy dose modifications should be discussed with the PARTNER Office/Chief Investigator.
Mucositis should be treated symptomatically as per local practice.

Hypersensitivity

Paclitaxel

The incidence of hypersensitivity with weekly Paclitaxel is 1-1.5% [83].

It is recommended that Paclitaxel infusion be stopped if mild symptoms of skin rash, flushing, and localised pruritus occur (Grade 1/2 hypersensitivity). Intravenous steroids and antihistamines are recommended with immediate slow re-challenge of Paclitaxel on recovery.
It is recommended that Paclitaxel infusion is stopped if Grade 2 or above symptoms of generalised pruritus or rash, dyspnoea, or hypotension occur and treatment with intravenous steroids and antihistamines are recommended. Patients with Grade 3 hypersensitivity should not be rechallenged with Paclitaxel.

It is recommended that if life-threatening symptoms occur, including bronchospasm, generalised urticaria, angio-oedema, severe hypotension (systolic blood pressure <100 mm Hg), or life-threatening anaphylaxis (Grade 4) Paclitaxel infusion is stopped immediately and treatment is given with intravenous corticosteroids, and intravenous antihistamines: with intramuscular (IM) epinephrine 1 mL 1:1000, if judged clinically indicated when IV corticosteroids and anti-histamines have not produced immediate improvement.

In the case of recurrent hypersensitivity reactions of Grade 3 and 4, despite adequate premedication, treatment should continue with Carboplatin alone.

Substitution with docetaxel is not allowed within this trial.

Table 7: NCI CTCAE grading of hypersensitivity reactions

**Adverse Event Category**	**1**	**2**	**3**	**4**	**5**
	**MILD**	**MODERATE**	**SEVERE**	**Life-threatening**	
**Allergic reaction**	Transient flushing or rash, drug fever <38°C (<100.4°F); intervention not indicated	Intervention or infusion interruption indicated; responds promptly to symptomatic treatment (e.g. antihistamines, NSAIDS, narcotics); prophylactic medications indicated for ≤24 hrs	Prolonged (e.g., not rapidly responsive to symptomatic medication and/or brief interruption of infusion); recurrence of symptoms following initial improvement; hospitalization indicated for clinical sequelae (e.g. renal impairment, pulmonary infiltrates)	Life-threatening consequences; urgent intervention indicated	Death
**Corresponding symptoms (any of)**	As above	Flushing; rash or urticaria; dyspnea; asymptomatic bronchospasm; drug fever >38°C (<100.4°F)	Symptomatic bronchospasm; allergy related oedema/angioedema hypotension or unresolving moderate symptoms	Anaphylaxis	

If it becomes necessary to discontinue Paclitaxel because of toxic effects then patients can continue treatment with single agent Carboplatin.

Carboplatin

Hypersensitivity reactions to Carboplatin should be managed according to local practice. Following mild-moderate hypersensitivity reaction, re-challenge should be possible with

increased prophylaxis and slowing of the infusion rate. Attempted retreatment following severe reactions, using a desensitisation protocol, should also be feasible, although this may result in exposure to moderate-high dose dexamethasone, so the issues related to this treatment need to be considered when instigating this type of de-sensitisation. After grade 3-4 hypersensitivity reactions immediate re-treatment should not be attempted.

If it becomes necessary to discontinue Carboplatin because of toxic effects then patients can continue treatment with single agent Paclitaxel.

Substitution with Cisplatin is not allowed within this trial.

Liver toxicity
Hepatotoxicity is not expected with either chemotherapy drug and other causes of liver enzyme elevation should be actively pursued.
If transaminases become elevated and are <3 x ULN (CTCAE v4.03 grade 1) then treatment can be continued as per Protocol without any dose modifications or delays. If transaminases become elevated to 3-5 x ULN (CTCAE v4.03 grade 2) then treatment can continue but dose reductions of Paclitaxel may be performed according to local practice at the discretion of the treating physician.
If transaminases become elevated to >5 x ULN (CTCAE v4.03 grade 3) then treatment with Paclitaxel should cease until resolution to <3 x ULN (CTCAE v4.03 grade 1).

Others
There are no dose modifications planned for alopecia, nausea, diarrhoea or constipation. These side effects should be treated with supportive medical measures. Non-steroidal anti-inflammatory agents can be used prophylactically, or symptomatically, as per local practice for the treatment of Paclitaxel-induced arthralgia/myalgia.
For any other adverse event of CTCAE v4.03 grade 4 severity considered at least possibly related to trial treatment, the patient should be discontinued from Protocol therapy and the PARTNER Office notified immediately with an SAE/AESI form.
To note, in the event of severe and persistent myelosuppression, Carboplatin should be discontinued to avoid infectious complications including fatal outcomes.
For any other adverse event of CTCAE v4.03 grade 3 severity considered at least possibly related to trial treatment, treatment should be withheld until recovery to grade 2 or less and subsequent treatment should be reduced (AUC 4 and 60mg/m^2 or AUC 3.5 and 45mg as a second step) and the PARTNER Office notified immediately with an SAE/AESI form.
There have been reports of reduced visual acuity due to cystoid macular oedema (CME) during treatment with Paclitaxel as well as with other taxanes. Patients with visual impairment during Paclitaxel treatment should seek a prompt and complete ophthalmologic examination. Discontinue Paclitaxel treatment if a CME diagnosis is confirmed. Clinicians should consider whether the benefits of restarting Paclitaxel treatment after CME resolution are expected to exceed the risks of further therapy.

12.4 Olaparib specific toxicity

In case of toxicities related to Olaparib and not to chemotherapy, please follow the recommendations in the tables 8 and 9 below.

In case of doubt, chemotherapy toxicities are more likely and therefore we recommend considering that the toxicities are related to the chemotherapy and to follow the recommendations of sections 12.3, initially.
During the safety stage of the trial (first 75 patients), CTCAE **Grade ≥ 3** which are not already reported as an SAE for example thrombocytopenia grade 3 (platelets= 36), must be reported as an AESI within 24 hours of becoming aware of the event.

Haematological toxicities

Table 8: Management of Olaparib haematological toxicity

Toxicity	Trial treatment dose adjustment
CTCAE grade 2	Investigator judgement to continue treatment with appropriate supportive treatment or if dose interruption, treatment should resume on D-2 (Research Arm 1) or D3 (Research Arm 2) of the following cycle.
Repeat CTCAE grade 2	Dose interruption until D-2 (Research Arm 1) or D3 (Research Arm 2) of the following cycle and dose reduction to 100mg twice a day on D-2-D10 (Research Arm 1) or D3-D14 (Research Arm 2) or 50mg twice a day on D-2-D10 (Research Arm 1) or D3-D14 (Research Arm 2) as second step. A further CTCAE grade 2 should lead to the discontinuation of Olaparib.
CTCAE grade 3 or 4	Dose interruption until recovery to CTCAE grade 1. Treatment should resume on D-2 (Research Arm 1) or D3 (Research Arm 2) of the following cycle with a dose reduction to 100mg twice a day on D-2-D10 (Research Arm 1) or D3-D14 (Research Arm 2).
Repeat CTCAE grade 3 or 4	Discontinue Olaparib.

Non-haematological toxicities

Table 9: Management of Olaparib non-haematological toxicity

Toxicity	Trial treatment dose adjustment
CTCAE grade 2	Investigator judgement to continue treatment with appropriate supportive treatment or if dose interruption, the treatment should resume on D-2 (Research Arm 1) or D3 (Research Arm 2) of the following cycle.
Repeat CTCAE grade 2	Where toxicity reoccurs following re-challenge with trial treatment, then the patient should be considered for dose reduction: 100mg twice a day on D-2-D10 (Research Arm 1) or D3-D14 (Research Arm 2) or 50mg twice a day on D-2-D10 (Research Arm 1) or D3-D14 (Research Arm 2) as second step or must permanently discontinue trial treatment.
CTCAE grade 3 or 4	Discontinue Olaparib.

Management of nausea and vomiting

Events of nausea and vomiting are known to be associated with Olaparib treatment. They are generally mild to moderate (CTCAE grade 1 or 2) in severity, intermittent and manageable on continued treatment.
Patients should receive appropriate anti-emetic treatment in accordance with local practice. Generally, a single agent antiemetic should be considered.

Pneumonitis:

During treatment, patients should be carefully monitored for signs and symptoms of

pneumonitis (i.e., episodes of transient or repeated dyspnoea with unproductive persistent cough or fever) and, if observed, immediate clinical evaluation and timely institution of appropriate management (emphasizing the need for corticosteroids if an infectious process has been ruled out as well as appropriate ventilation and oxygen support when required).

If new or worsening pulmonary symptoms (eg, dyspnoea) or radiological abnormalities occur in the absence of a clear diagnosis, an interruption in study treatment dosing is recommended and further diagnostic workup (including a high resolution CT scan) should be performed to exclude pneumonitis.

If pneumonitis is suspected, it is recommended to interrupt treatment.

Physical examination; Signs and symptoms (cough, shortness of breath and pyrexia, etc.) including auscultation for lung field will be assessed and SpO2 (saturation of peripheral oxygen) should be recorded. Any additional assessments if required, will be performed to enhance the investigation and diagnosis of potential cases of pneumonitis.

Following investigation, if no evidence of abnormality is observed on CT imaging and symptoms resolve, then study treatment can be restarted, if deemed appropriate by the Investigator. If significant pulmonary abnormalities are identified, these need to be discussed with the Study Physician.

Renal impairment

If subsequent to study entry and while still on study therapy, a patient's estimated GFR falls below the threshold for study inclusion (≥51 ml/min), retesting should be performed promptly.

A dose reduction is recommended for patients who develop moderate renal impairment (calculated creatinine clearance by Cockcroft-Gault equation or based on a 24 hour urine test of between 31 and 50 ml/min) for any reason during the course of the study: the dose of Olaparib should be reduced to 200 mg twice daily.

Because the GFR determination is only an estimate of renal function, in instances where the GFR falls to between 31 and 50 mL/min, the investigator should use his or her discretion in determining whether a dose change or discontinuation of therapy is warranted.

Olaparib has not been studied in patients with severe renal impairment (creatinine clearance ≤ 30 ml/min) or end-stage renal disease; if patients develop severe impairment or end stage disease is it recommended that Olaparib be discontinued.

[redacted]

[redacted]

[redacted]

[redacted]

[redacted]

[redacted]

[redacted]

[redacted]

13 Procedures and assessments

See Appendix 3 & 4 for a Timetable of events.

13.1 Prior to each cycle of Partner treatment

- Updated medical history, ECOG and vital signs.
- Adverse event assessment.
- Full Blood Count, Liver and Renal Function Tests (recommended timing is within the last 7 days), as per local practice.

  Blood tests should be carried out **prior the start of the Olaparib (Research arm 1) or prior to the chemotherapy (control Arm, Research Arm 2)** (recommended timing is within 7 days), as per local practice.
- Completion of the Chemotherapy Induced Neuropathy questionnaire prior cycle 1, prior cycle 2-5, +/- full two point discrimination assessment and a neuropathy scoring as applicable (dependent on questionnaire score).
- Obtain fresh tissue biopsy prior cycle 1, if applicable (see Fresh Tissue sub-study in section 18.3).
- Completion of QoL questionnaire prior to randomisation, if applicable (see QoL sub-study, in section 15.4).
- Obtain sequential blood sample prior to cycles 1, 2, and 5, if applicable (see Sequential Blood sub-study in section 18.4).
- Obtain blood sample for anti-Mullerian hormones study prior cycle 1 , if applicable (see Sequential Blood sub-study in section 18.4).

All criteria below must be met for prescription of chemotherapy +/- Olaparib on day 1 of each cycle:

  - Absolute neutrophil count ≥ 1.5 x 10^9/L for cycle 1 and 1.0 x 10^9/L for following cycles.
  - Platelet count ≥ 75 x 10^9/L
  - Total bilirubin ≤ 1.5 x ULN

- ALT or AST ≤ 2.5 x ULN
- Serum creatinine ≤1.5 ULN

13.2 Prior to D8 and D15 of Paclitaxel

- Full Blood Count is required before each administration of Paclitaxel.

All criteria below must be met for prescription of Paclitaxel on day 8 and 15:

- Absolute neutrophil count ≥ 0.5 x 10^9/L.
- Platelet count ≥ 50 x 10^9/L if no active bleeding.

13.3 Mid-chemotherapy (After cycle 4, prior to cycle 5)

- Radiology scan after cycle 4 (prior to cycle 5) +/- positional marking (see section 13.7).
- Completion of the Chemotherapy Induced Neuropathy questionnaire, +/- full two point discrimination assessment and a neuropathy scoring if applicable (dependent on questionnaire score) (see section 15.3).
- Obtain fresh tissue biopsy, if applicable (see Fresh Tissue sub-study in section 18.3).
- Obtain sequential blood sample prior to cycle 5, if applicable (see Sequential Blood sub-study in section 18.4).
- Completion of QoL questionnaire prior to cycle 5, if applicable (see QoL sub-study, in section 15.4).

13.4 After end of chemotherapy and prior to surgery

- Radiology scan after cycle 7 +/- positional marking (see section 13.7).
- Obtain research blood sample post-chemotherapy.
- Full Blood Count, Liver and Renal Function Tests.
- Updated medical history, ECOG and vital signs.
- Adverse event assessment.
- Completion of Chemotherapy Induced Neuropathy questionnaire, +/- full two point discrimination assessment and a neuropathy scoring as applicable (dependent on questionnaire score) (see section 15.3).
- Obtain fresh diagnostic biopsy tissue (if not possible at surgery) if applicable (see Fresh Tissue sub-study in section 18.3).
- Obtain sequential blood sample and blood for anti-Mullerian hormones sub-study after cycle 7 (prior to surgery) if applicable (see Sequential Blood sub-study in section 18.4).
- Completion of QoL questionnaire if applicable (see QoL sub-study, in section 15.4).

[redacted]

- [redacted]
- [redacted]
- [redacted]

[redacted]

[redacted]

13.5 After surgery

- Obtain the pathology samples and reports (slides and block) as per section 18.2.
- If applicable, obtain sequential blood sample immediately after surgery when possible and a few weeks after surgery (see Sequential Blood sub-study in section 18.4).

13.6 Long Term Follow-up

Follow-up visits should follow standard practice but preferably made annually based on the surgery date anniversary. Following discharge, site staff should attempt to contact patients at least annually until the 10 year follow-up period is complete for that patient. Phone or email contact is allowed. See schedule of follow-up visits in appendix 5.
During follow-up, attempts should be made to obtain:

- Updated medical history.
- Completion of Chemotherapy Induced Neuropathy questionnaire, +/- full two point discrimination assessment and a neuropathy scoring if applicable (dependent on questionnaire score) (see section 15.3).
- Obtain sequential blood sample if applicable at 6 and 12 months (at 12 months, with blood for anti-Mullerian hormones sub-study) and then yearly for 5 years (see Sequential Blood sub-study in section 18.4).
- Completion of QoL questionnaire if applicable (see QoL sub-study, in section 15.4).
- Obtain tumour block and pathology report of relapse as per section 18.2. Obtain fresh tissue and sequential blood sample at relapse as applicable (see section 18.3 and 18.4).

13.7 Clinical and radiological monitoring during neo-adjuvant chemotherapy

Radiology scans (US or MRI) should be performed before treatment, after cycle 4, and after cycle 7 (before surgery) using comparable methods throughout the trial. *An extra post cycle 6 scan will be performed for those patients included into PartnerING pathway. In these cases, the end of treatment scan will be performed after Cycle 9 of PartnerING treatment.*

Positional marking of the tumour is recommended to be performed on all patients. Best practice, where possible, is to place a clip in the centre of the tumour at the time of an ultrasound / biopsy (see section 13.8).

Variation in timing of radiology scans across tumours or patients can compromise the analysis. Therefore, we ask that the time points below are followed as closely as possible:

Before the first cycle of chemotherapy: Radiology scan as close as possible to the first cycle of treatment.

After completion of the first 4 cycles of neoadjuvant chemotherapy: scan will be performed to assess radiological response. The date of measurement should be as close to day 21 of cycle 4 as possible, and before administration of cycle 5.

After completion of 6 cycles (only for PartnerING participating centres) *of neoadjuvant chemotherapy: scan will be performed to assess evidence of residual disease. This additional scan will only be performed in those centres participating in PartnerING*.

After completion of treatment: scan will be performed to assess radiological response. The date of measurement must be as close to surgery as possible. This last scan can be used for positional marking of the tumour, although it is preferable to place clips either before treatment or at the midway point.

Radiological response
At each radiological measurement, the sum of the longest single diameter of all measurable tumours (including axillary Lymph Nodes if enlarged) must be recorded on first section of each of the Radiology Forms in the PARTNER CRF.
In the second section of the Radiology Forms a response must be categorised, this must be calculated from the 'total tumour size' defined as the **Sum of the Longest single Diameter (SLD) of each measurable tumour located in the breast only.**
Radiology measurements taken at both the midway and end of treatment scans must be compared to smallest SLD on-trial in order to assess the radiological response.

Radiological response criteria are as follows (RECIST v1.1):

Complete Response: Disappearance of all extra-nodal target lesions. All pathological lymph nodes must have decreased to <10 mm in short axis.

Partial Response: At least a 30% decrease in the SLD of target lesions, taking as reference the baseline sum diameters.

Stable Disease: Neither sufficient shrinkage to qualify for a partial response nor sufficient increase to qualify for progressive disease, taking as reference the smallest SLD on-trial.

Progressive Disease: SLD increased by at least 20% from the smallest value on trial (including baseline, if that is the smallest). The SLD must also demonstrate an absolute increase of at least 5mm. (Two lesions increasing from 2 mm to 3 mm, for example, does not qualify).
Additionally, any appearance of one or more new lesions is classed as progressive disease.

The Radiology Forms in the CRF must be signed by the responsible radiologist or PI at the individual sites.

The PARTNER team may request de-identified images from participating patients as part of the trial.

13.8 Surgery

Sentinel node biopsy and axillary surgery

Axillary Lymph Node response is a very strong indicator of outcome. Recent trials have shown that early removal of axillary nodes might alter valuable prognostic and response information in the axilla. For example, approximately 4% of patients with pCR in the breast do NOT have pCR in the axillary LN. It would not be beneficial for such patients to have SLN removed prior neoadjuvant chemotherapy as they need axillary clearance.
Therefore, **we do not advise SLN biopsy pre-treatment**. We recommend pathological axillary LN assessment at surgery and axillary clearance as per local practice.

Positional Marking

Positional marking of the tumour is recommended to be performed on all patients. In previous neoadjuvant studies it has sometimes been difficult to locate tumours at the time of definitive surgery, particularly if there has been complete radiological response. Best practice, where possible, is to place a clip in the centre of the tumour, at the time of an extra biopsy (either pre-treatment or at the time of a second ultrasound/biopsy where this is being performed), so that definitive surgery (if this is not mastectomy) will remove the site of the original tumour. If the definitive surgical intention is mastectomy because of multi-focal disease, disease beneath the nipple, inflammatory or locally advanced breast cancer, then marking clips are still necessary to assist the pathologist in identifying the tumour site in case of significant pathological response [84]).

Timing of surgery

The high rates of local recurrence which have been reported in previous neoadjuvant studies when surgery is not part of primary treatment, leads us to mandate all Investigators to perform complete excision with clear margins after chemotherapy. Surgery must be planned to occur at completion of the Protocol stated seven cycles of chemotherapy, at any time following the last day of the final cycle of chemotherapy (Cycle 7 Day 21). A surgery date within 3 weeks of that day is strongly recommended.

For patients who have followed the PartnerING pathway, surgery must be planned to occur following completion of the protocol. A surgery date within 3 weeks of the last day of the final cycle of the combination therapy (Cycle 9 Day 28) is strongly recommended. Please note that PartnerING treatment should be stopped at least 3 days prior to planned surgery. No stoppage of study treatment is required for any needle biopsy procedure.

Breast Surgery type

At the time of diagnosis, the surgeon will decide on the intended surgery to be carried out after chemotherapy. For larger but peripheral lesions breast conserving surgery may be possible pending a good clinical and radiological response to neoadjuvant treatment.

Patients with clinically inflammatory or large locally advanced cancers at diagnosis, or where the primary tumour is under or close to the nipple, are not generally offered breast-conserving surgery but mastectomy, with or without immediate reconstruction. Patients with smaller, peripheral, locally advanced tumours who respond well to chemotherapy may be offered breast-conserving surgery. Patients with multiple lesions are generally offered mastectomy, with or without immediate reconstruction.

Prophylactic (preventative) surgery to remove both breasts (called bilateral risk reducing mastectomy (RMM)) can reduce the risk of breast cancer in women who have a strong family history of breast and/or ovarian cancer, who have a deleterious (disease-causing) mutation in the BRCA1 gene or the BRCA2 gene. Decisions regarding risk reducing surgery are to be made by the responsible surgeon and a genetic counsellor in conjunction with the patient. There is no contra-indication for BRCA mutation carriers to have radiotherapy and therefore it is clinically acceptable for BRCA patients to have initial breast conserving surgery followed by radiotherapy as immediate management strategy and opt for bilateral RRM in future.

13.9 Adjuvant Hormonal Treatment after Surgery

Following completion of neoadjuvant chemotherapy and definitive surgery, women with ER-positive disease would expect to be offered adjuvant hormonal therapy for a minimum of at least five years. Choice and duration of endocrine therapy is at the discretion of the responsible clinician in accordance with local practice.

13.10 Radiotherapy

Radiotherapy will be given after definitive surgery according to local practice. It is recommended within 4 weeks of surgery and no later than 8 weeks.

13.11 Disease Progression

Signs of disease progression at any stage during the neoadjuvant treatment should be investigated radiologically. If progression is confirmed the patient should discontinue trial treatment but remain on trial for follow-up and sampling purposes – see section 10.6.1.
It is recommended that if progression occurs during the first phase of neoadjuvant chemotherapy (Paclitaxel/Carboplatin +/- Olaparib); patients should proceed to the second phase of treatment (anthracycline-based chemotherapy) or to definitive surgery. This decision is at the discretion of the responsible clinician.
If progression occurs during the second phase of treatment (anthracyclines) then immediate surgery followed by radiotherapy should be considered.

If progression occurs during the administration of PartnerING therapies, patients will be directed to immediate surgery.

Disease progression of any patient should be communicated to the PARTNER Office. Progression must be recorded on the Relapse/Progression and Second Primary Form, and a Treatment Discontinuation/Withdrawal Form must also be completed and sent to the PARTNER Office within 7 days.

13.12 Follow-Up and Relapse

Follow-up data will be requested annually based on the surgery date anniversary. Site staff should attempt to contact patients at least annually until the 10 year follow-up period is complete for that patient. Phone or email contact is allowed.
Where necessary, follow-up data will be obtained from patients' GPs or, in UK, through the Office for National Statistics (ONS) using the patients' NHS numbers.

Long-term follow-up will include assessment of: date and site of first relapse second primary, and date and cause of death, as applicable. This is necessary for the trial's secondary endpoints of DFS, OS and the other long term survival analyses. As soon as

definite confirmation has been obtained, a Relapse/Progression and Second Primary form should be completed and returned to the PARTNER office. Patients who relapse should remain in follow-up.

13.13 Concurrent studies

Participation of PARTNER patients into other clinical trials will be considered on an individual basis, and should ideally be discussed with the PARTNER office prior to randomisation.

It is acceptable for patients to enter into the POSNOC trial http://www.posnoc.co.uk/ whilst participating in PARTNER.

14 Assessment of Safety and Safety Reporting

14.1 Definitions (table 10)

Term	Definition
Adverse event (AE)	Any untoward medical occurrence in a patient or clinical trial subject administered a medicinal product and which does not necessarily have a causal relationship with this treatment.<br><br>An adverse event can therefore be any unfavourable and unintended sign (including a clinically significant abnormal laboratory finding), symptom, or disease temporally associated with the use of an investigational medicinal product, whether or not considered related to the investigational medicinal product.
Adverse reaction to an investigational medicinal product	All untoward and unintended responses to an investigational medicinal product related to any dose administered.<br><br>All adverse events judged by either the reporting Investigator or the Sponsor as having a reasonable causal relationship to the medicinal product quality as adverse reactions. The expression reasonable causal relationship means to convey in general that there is evidence or argument to suggest a causal relationship.
Unexpected adverse reaction	An adverse reaction, the nature or severity of which is not consistent with the Reference Safety Information (RSI).<br><br>When the outcome of the adverse reaction is not consistent with the applicable RSI it should be considered unexpected.
Serious Adverse Event (SAE) or Serious Adverse Reaction (SAR)	Any untoward medical occurrence or effect that at any dose:<br>• results in death<br>• is life-threatening* i.e. the subject was at risk of death at the time of event<br>• requires hospitalisation or prolongation of existing inpatient hospitalisation**<br>• results in persistent or significant disability or incapacity<br>• is a congenital anomaly or birth defect<br>• is an important medical event***
Reference Safety Information (RSI)	The information used for assessing whether an adverse reaction is expected. It contains a list of all expected Adverse Reactions to the drugs and it is used when making a determination as to the expectedness of the adverse reaction. **For this trial, the latest MHRA approved RSI are:**<br><br>**Carboplatin: Section 4.8 of the Hospira UK Limited SmPC**<br><br>**Paclitaxel: Section 4.8 of the Hospira UK Limited SmPC**<br><br>**Olaparib: Section 5.6 (Reference Safety Information) of the IB**<br><br>**AZD6738: Section 5.6 table 33 (Reference Safety Information) of the IB**<br><br>**Durvalumab: Section 5.6 table 34 (Reference Safety Information) of the IB**
Suspected Unexpected Serious Adverse Reaction (SUSAR)	A serious adverse reaction, the nature and severity of which is not consistent with the information set out in the latest MHRA approved version of the RSI.

* Life-threatening in the definition of a serious adverse event or serious adverse reaction refers to an event in which the subject was at risk of death at the time of event; it does not refer to an event which hypothetically might have caused death if it were more severe.

**Hospitalisation is defined as an inpatient admission, regardless of length of stay, even if the hospitalisation is a precautionary measure for continued observation. Hospitalisation for a pre-existing condition, including elective procedures, which has not worsened, does not constitute a serious adverse event.

*** Some medical events may jeopardise the subject or may require an intervention to prevent one of the above characteristics/ consequences. Such events (hereinafter referred to as 'important medical events') should also be considered as 'serious'.

Investigators and Sponsors MUST follow the following procedures to report adverse events or reactions.

14.2 Evaluation and recording of adverse events (non-serious)

The Sponsor expects that adverse events related to protocol procedure are recorded from the point of Informed Consent regardless of whether a patient has yet received a medicinal product.

Adverse events will be recorded throughout the entire chemotherapy treatment on the Post-cycle Toxicities and Checklist Forms.

However, the following should NOT be recorded:

- A pre-existing condition, unless the condition worsens or episodes increase in frequency during the reporting period and the Investigator deems this related to use of the trial drugs.
- Symptoms relating to disease progression unless the Investigator deems them related to the use of trial drugs.

Recording of adverse events is to be as follows:

- Individual AEs must be assessed for seriousness.
- Individual **AEs and ARs** should be documented **in the medical notes and** on the Post-cycle Toxicities and Checklist forms **in the toxicity section (check box list) to be completed for each cycle of treatment**.
- All AEs experienced will be graded for severity according to the NCI CTCAE Toxicity Criteria (Version 4.03). CTCAE v4.03 can be downloaded from the following URL: http://ctep.cancer.gov/reporting/ctc.htmL

14.3 Adverse Events of Special Interest (AESI)

During the safety stage of the trial (first 75 patients), AESI associated with the IMPs are defined as **Grade ≥ 3** which are not already reported as an SAE for example thrombocytopenia grade 3 (platelets=36). AESIs will be recorded on the separate AESI form but be reported to the PARTNER office the same way as for SAEs (section 14.4.6) i.e. any AESI must be notified within 24 hours of becoming aware of the event.

14.4 Reporting serious adverse events

14.4.1 General principles and responsibilities

Site / PI responsibilities: Each Principal Investigator needs to report serious adverse events to the Chief Investigator via the PARTNER Office using the trial specific SAE form **within 24 hours of their awareness** of the event. The completed SAE form can be emailed. Details for reporting SAEs can be found in section 14.4.6.

CI/Sponsor responsibilities: The Chief Investigator is responsible for ensuring the assessment of all SAEs for expectedness and relatedness is completed and the onward notification of all SAEs to the Sponsor and AstraZeneca immediately but not more than 24 hours of first notification. The Sponsor must keep detailed records of all SAEs reported to them.

The Chief Investigator is also responsible for prompt reporting of all serious adverse event findings to the Competent Authority or Regulatory Agency of each concerned Member State if they could:

- adversely affect the health of subjects
- impact on the conduct of the trial
- alter the risk to benefit ratio of the trial
- alter the Competent Authority's authorisation to continue the trial in accordance with Directive 2001/20/EC.

14.4.2 Reporting period for SAEs

If a patient experiences an SAE after the informed consent document is signed, but before receiving the first dose of trial drug, the event will be reported only if the Investigator believes that the event may have been caused by a Protocol procedure.

Details of all SAEs will be documented and reported until 21 days post-chemotherapy (i.e. 21 days from last administration of a trial drug: Carboplatin, Paclitaxel, Olaparib or anthracyclines).

SAEs occurring 21-days after the end of the chemotherapy should be reported only if the Investigator believes that a trial drug or a Protocol procedure may have caused the event.

Progression alone should not be reported as an SAE, except in circumstances where a patient progresses and experiences any event which constitutes an SAE as defined above.

[redacted]

14.4.3 Evaluation of SAEs by the Principal Investigator

Individual serious adverse events must be evaluated by a registered Investigator at each trial site. This includes the evaluation of its seriousness, causality and any relationship between the investigational medicinal product(s) and/or concomitant therapy and the serious adverse event.

14.4.4 Table 11: Assessment of causality

Term	Definition
**Definitely**	A causal relationship is clinically/biologically certain. **This is therefore an Adverse Reaction.**
**Probably**	A causal relationship is clinically / biologically highly plausible and there is a plausible time sequence between onset of the AE and administration of the

	investigational medicinal product and there is a reasonable response on withdrawal. **This is therefore an Adverse Reaction.**
**Possibly**	A causal relationship is clinically / biologically plausible and there is a plausible time sequence between onset of the AE and administration of the investigational medicinal product. **This is therefore an Adverse Reaction.**
**Unlikely**	A causal relation is improbable and another documented cause of the AE is most plausible. **This is therefore an Adverse Event.**
**Unrelated**	A causal relationship can be definitely excluded and another documented cause of the AE is most plausible. **This is therefore an Adverse Event.**

Unlikely and Unrelated causalities are considered NOT to be trial drug related.
Definitely, Probably and Possibly causalities are considered to be trial drug related.

14.4.5 Assessment of expectedness

The PARTNER office will use the latest MHRA approved version of the RSI, for reference this can be found in the safety reporting section of the Investigator Site File, to determine the expectedness of all serious adverse events and reactions. An unexpected adverse reaction is one not previously reported in the latest MHRA approved RSI or one that is more frequent or more severe than previously reported.
If a SAR is assessed as being unexpected, it becomes a SUSAR.

14.4.6 Notification of SAEs

In the case of an SAE, the Investigator or delegated deputy must within 24 hours of the event occurring:

Complete a 'Serious Adverse Event Form' and email it to the PARTNER Office (Cambridge Clinical Trials Unit – Cancer Theme) on

Email: cuh.partner@nhs.net

In the case of death, life-threatening events or SUSARs, in addition to emailing an SAE form to the PARTNER Office, please TELEPHONE the Trial Coordinator at the PARTNER Office (on day of awareness), on

Tel number: 01223 348 447

The SAE form should be completed and signed by an appropriate member of the site trial team and emailed as above. A trial investigator must counter sign as soon as possible. Even if only limited information is initially available, this should be provided and emailed on an SAE form. Further details should be submitted as soon as they become available.

Where a reported SAE is judged to be both related to the IMP and unexpected (i.e. is a SUSAR), the Investigator must assist the PARTNER Office by supplying all requested information to the Sponsor to allow the appropriate regulatory reporting timeline to be met.

In the case of an SAE, the subject must be followed-up until clinical recovery is complete and laboratory results have returned to normal, or until the disease has stabilised. Follow-up may continue after completion of Protocol treatment if necessary. Follow-up

information will be noted on the SAE Form by ticking the box marked 'follow-up' and sending to the PARTNER Office as information becomes available. Extra annotated information and/or copies of anonymised test results should also be provided where available.

14.5 Reporting of Suspected Unexpected Serious Adverse Reactions (SUSARs)

All suspected serious adverse reactions related to one of the investigational medicinal products (Olaparib, Carboplatin, Paclitaxel, AZD6738, Durvalumab) that are **unexpected** are subject to expedited reporting.

14.5.1 Who should report and whom to report to?

The Sponsor directly notifies the MHRA of any SUSARs within the legally required timeframes.
The Sponsor delegates the responsibility of notification of SUSARs to the Chief Investigator.
The Chief Investigator must report SUSARs to the:

- the Research Ethics Committee (REC),
- AstraZeneca,
- the Sponsor within the legally required time frames.

The Chief Investigator shall inform all Investigators concerned of relevant information about SUSARs that could adversely affect the safety of subjects.

14.5.2 When to report?

Fatal or life-threatening SUSARs

All parties listed in 14.5.1 must be notified as soon as possible but no later than **7 calendar days** after the trial team and Sponsor has first knowledge of the minimum criteria for expedited reporting. In each case relevant follow-up information should be sought and a report completed as soon as possible. It should be communicated to all parties within an additional **8 calendar days**.

Non fatal and non life-threatening SUSARs

All other SUSARs and safety issues must be reported to all parties listed in 9.4.1 as soon as possible but no later than **15 calendar days** after first knowledge of the minimum criteria for expedited reporting.

Follow-up reports of SUSARs

In case of incomplete information at the time of initial reporting, all the appropriate information for an adequate analysis of causality should be actively sought from the reporter or other available sources. Further available relevant information should be reported as follow-up reports within an additional **8 calendar days for a fatal of life-threatening SUSARs or as soon as possible otherwise.**
In certain cases, it may be appropriate to conduct follow-up of the long-term outcome of a particular reaction.

14.5.3 How to report?

Minimum criteria for initial expedited reporting of SUSARs

Information on the final description and evaluation of an adverse reaction report may not be available within the required timeframes for reporting. For regulatory purposes, initial expedited reports should be submitted within the time limits as soon as the minimum

following criteria are met:

a) A suspected investigational medicinal product,
b) An identifiable subject (e.g. trial subject ID number),
c) An adverse event assessed as serious and unexpected, and for which there is a reasonable suspected causal relationship,
d) An identifiable reporting source.

And when available and applicable:

e) A unique clinical trial identification (e.g. EudraCT number),
f) A unique case identification (e.g. sponsors case number).

Format of the SUSAR reports

Electronic reporting is the expected method for expedited reporting of SUSARs to the Competent Authority. The format and content as defined by the Competent Authority should be adhered to.

14.6 Pregnancy Reporting

Pregnant trial patients must immediately cease ALL trial treatments.

All pregnancies within the trial (either a trial patient or a patient's partner) should be reported to the PARTNER Office using the relevant Pregnancy Reporting Form within 24 hours of first knowledge of the event. The PARTNER Office will immediately inform the Sponsor of the pregnancy. In PARTNER, the requirement to report pregnancy stops after the patient's surgery.

Patient becoming pregnant during the course of the trial treatment must be followed up until birth or termination of pregnancy.
In the event that the trial patient is a male and his partner becomes pregnant during the course of the trial, the pregnancy must be followed up until birth or termination.

Pregnancy is not considered an AE unless a negative or consequential outcome is recorded for the mother and/or child/foetus. If the outcome meets the serious criteria, this would be considered an SAE.

Figure 1. Safety Reporting Flowchart for Participating Sites

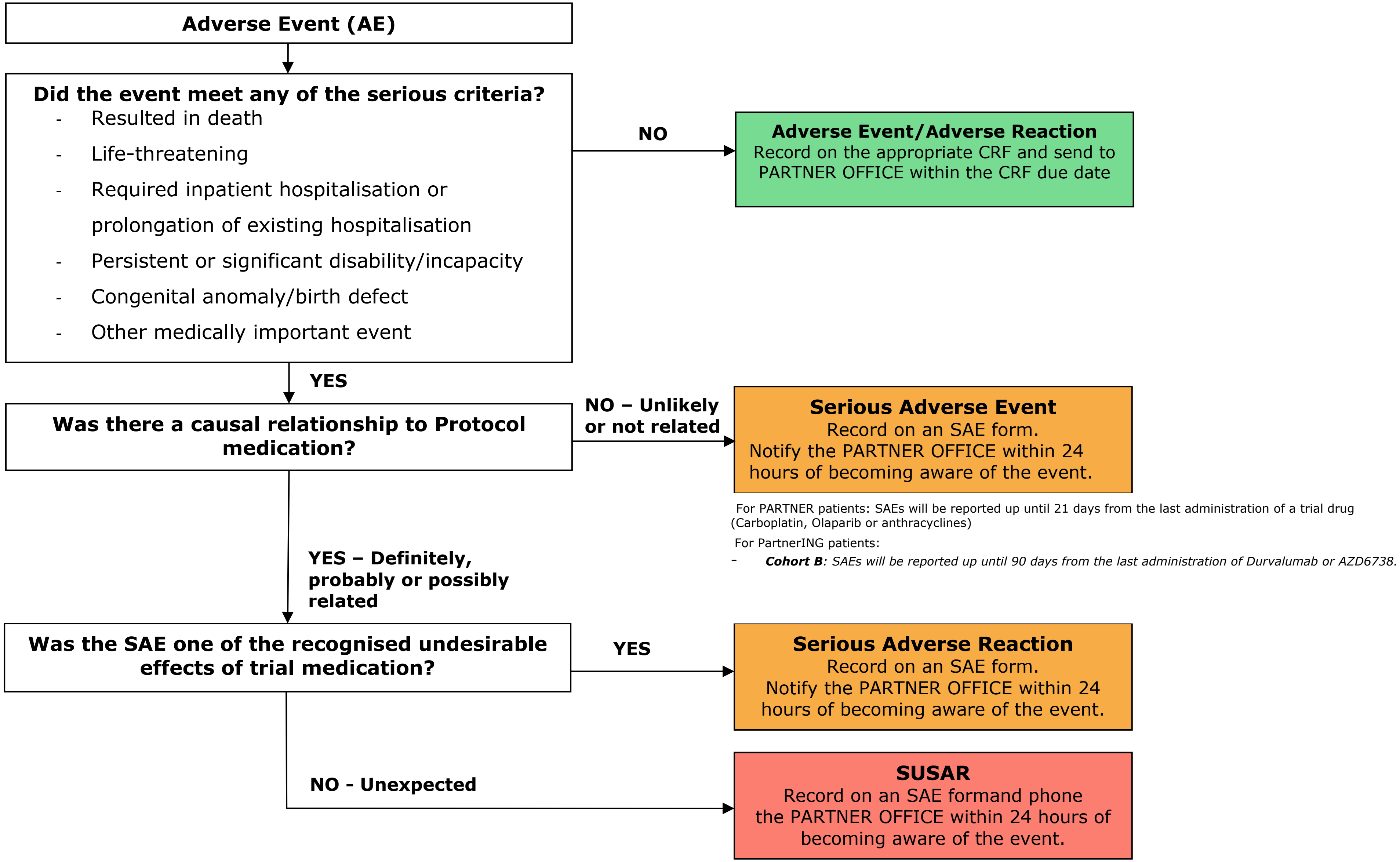

15 Evaluation of Results : Definitions and response / evaluation of outcomes measures

15.1 Pathological Complete Response

The primary endpoint for the trial is pathological complete response (pCR) following neoadjuvant treatment, and is defined below:

Pathological Complete Response rates (tumour and lymph nodes) after neoadjuvant chemotherapy are defined as no residual invasive carcinoma within the breast (DCIS permitted) AND no evidence of metastatic disease within the lymph nodes [85]**.**

This will be assessed by central review (2 readers) of the surgery pathology reports.

Local assessment of pathological complete response

Histopathological examination

Histopathological assessment will be carried out at diagnosis and repeated on the definitive surgical specimens; either wide local excision or mastectomy, and axillary clearance. These specimens will be handled and reported according to national guidelines for non-operative procedures and for pathology specimen handling (NHS BSP Publication No 50. 2001 and NHS BSP Publication No 58, 2005 updated by The Royal College of Pathologists in June 2016). It should be noted that the macroscopic search for tumours which have undergone significant pathological response will be aided by clear communication of the original sites of the tumour, on the Radiology CRF, and histology request form or by marking the sites with clip or suture. Pathologists should sample this area thoroughly.

Grading of pathological response

Ideally, a comment as to the degree of chemotherapy effect should be included in the pathology report. However histopathological response will also be assessed centrally using the Residual Cancer Burden (RCB) scoring system by the trial pathological advisors using H&E slides from blocks taken at diagnosis and surgery (tumour +/- lymph nodes). The RCB is an online tool for the quantification of residual disease that uses tumour size, residual tumour cellularity, number of positive lymph nodes and the size of the largest metastasis to generate a continuous numerical score [42]. It is simple to apply, reproducible, and has been clinically validated with long-term follow-up data [86]

RCB is calculated as a continuous index combining pathological measurements of primary tumour size and cellularity; nodal metastases (number and size).

Table 12: RCB scoring

RCB	Amount of residual tumour remaining after neoadjuvant chemotherapy
RCB 0	pCR
RCB I	Minimal residual disease
RCB II	Moderate residual disease
RCB III	Extensive residual disease

Central assessment of the pathological complete response

pCR will be assessed first by central review (2 readers) of the surgery pathology reports (primary endpoint).

Also, all cases that have not achieved a pCR will be centrally reviewed by a trial pathologist based on the diagnostic and surgery slides (secondary endpoint).

15.2 Overall Survival

Survival endpoints will be measured from the date of randomisation and will be reported for all deaths due to all causes. The cause of death is to be recorded in all instances.

15.3 Neurotoxicity

At the specific time points mentioned below, participating patients will be provided with a chemotherapy induced neuropathy questionnaire based on a list of questions to score from 0 to 4. The responsible clinician will review the questionnaire during the visit.
If the patient scored:

- 2 or more in at least one question
- or 1 or more in at least two questions

a clinical assessment consisting of a full two points discrimination test is required, as well as a symptoms scoring. Data will be collected within the CRF.

Questionnaire +/- clinical assessment and scoring are expected at the following time points:

- Prior to commencement of chemotherapy (baseline),
- After each cycle of Paclitaxel/Carboplatin (cycle 1-4),
- After cycle 7,
- At 3 months; 6 months; 12 months and 24 months. It is permitted to ask patients to complete the questionnaire at home and return it to the Research Team by post or email.

15.4 Quality of life – Optional sub-study

Participation in the Quality of Life sub-study is optional. The quality of life sub-study will be offered to all randomised patients. All patients who consent to undertake the QoL assessments will receive EQ-5D questionnaires. In addition female patients will also receive the FACT-B questionnaire for completion. Participating patients will be provided with questionnaires by trial site staff at the relevant time points as follows:

- Prior to randomisation,
- Following completion of first four cycles of chemotherapy (approx. 12 weeks post-randomisation),
- Following completion of seven cycles of chemotherapy (approx. 4 months post-randomisation),
- Following completion of surgery and radiotherapy (approx. 6 months post-randomisation),
- Then annually for 2 years from completion of surgery (at follow-up visits 12 and 24 months post-surgery) to document long-term effects on quality of life.

Details of treatment related toxicities will be collected at each cycle of treatment using the NCI CTCAE Version 4.03 scoring system. Data will also be collected on hospital admissions and use of G-CSF.

15.5 Data related to Covid-19

Due to the Covid-19 pandemic, relevant data from randomised patients will be collected and will include but not be limited to the following:

- Effects on the management of trial patients (e.g. trial treatment, surgery, assessments and procedures)
- Test details for Covid-19 (e.g. type of test, test results)
- Symptoms and treatments for Covid-19

16 Statistics

16.1 Sample size

This is an open label randomised 3-stage phase II/III trial. In Stage 1 and 2, patients will be randomised with a ratio 1:1:1 to either control or one of the two research arms. At the end of Stage 2, one of the two research arms will be dropped. The recruitment will continue into all three arms while waiting for the results of analysis of Stage 1 and Stage 2. Patients will then be randomised with a ratio 1:1 to either control arm or the selected research arm. It is planned to recruit a minimum of **780** patients in total. The recruitment of TNBC non-gBRCA and gBRCA patients will be completed independently. It is planned to recruit a minimum of 478 TNBC non-gBRCA and 188 gBRCA patients.

Stage 1 (n=75, 25 patients in each of the two research arms) **– Safety.**
The primary outcome measure in stage 1 is safety within the research arms. Although all toxicities will be considered, particular consideration will be given to CTCAE grade 3 and 4 toxicities that appear to be associated with Olaparib especially grade 3 and 4 Febrile neutropenia and thrombocytopenia, or events that require dose reduction or discontinuation of Olaparib. If there is no evidence that the grade 3 and 4 adverse events as specified above exceed 35% (this would have been if more than 14 out of 25 patients for each of the research arm, or more than 24 out of 50 patients combining both research arms), the trial will continue to Stage 2. Otherwise, the research regimens will be amended. Safety will also be compared between the control and experimental arms in an exploratory manner.
This stage was completed in February 2018 and the trial continued to Stage 2.

Stage 2 (n=159, 53 in each of the two research arms) – **Selection of a research arm: pCR rate post-surgery primarily together with completion rate of Olaparib Protocol treatment.** It is a "pick-the winner" design with 53 patients in each of research arms. This is to have a 90% power, 5% one-sided significance level to test null hypothesis of pCR ≤ 35% versus an alternative hypothesis of pCR ≥ 55% in each of the research arms.
This stage was completed on 20th May 2019 and the trial continued to Stage 3. Research Arm 1 (Olaparib Day -2 to 10) was dropped and Research Arm 2 (Olaparib Day 3 to 14) was selected for stage 3.

Recruitment will continue while waiting for the results of analysis based on the 106 patients randomised in both research arms. Assuming that it will take 9 months (6 month to surgery + 3 months data cleaning and analysis) from the date when 106 patients are randomised in both research arms to the date when decision on Stage 2 is made, there will be approximately 90 patients randomised in the trial (60 between the two research arms). All 166 patients randomised to the two research arms until end of Stage 2 will be followed-up, which will provide information between the two research arms, a difference of pCR around 18% between the two research arms could be detected with a 10% significance level (two-sided) and 80% power.

Stage 3 (n=666 between control and the research arm selected in Stage 2**) - Efficacy**
The primary outcome measure is pCR. Based on the evidence from GeparSixto and CALGB 40603 [20–22] the anticipated pCR is **~45-55%** for TNBC non-gBRCA patients and **~50-60%** for gBRCA patients, receiving platinum-based chemotherapy. The trial is powered to detect an absolute improvement of **15%** for TNBC non-gBRCA and **20%** for gBRCA patients by adding Olaparib to chemotherapy. A pre-planned HRD subgroup analysis will be included into the outcome for the non-BRCA cohort.

Outcomes for each cohort (TNBC non-gBRCA and gBRCA) will be independently analysed and published. A joined data analysis will take place, once recruitment for the two cohorts and independent analysis are complete.

TNBC non-gBRCA patients

Sample size for non-gBRCA patients with significance level of 0.05 and 90% power to detect 15% difference: a total of 434 to 464 patients are required depending on the pCR rate in the standard arm.

Standard	Research	Total no. of patients
45%	60%	464
**50%**	**65%**	**454**
55%	70%	434

gBRCA Patients

Sample size for gBRCA patients with significance level of 0.05 and 80% power to detect 20% difference: a total of 164 to 186 gBRCA patients are required depending on the pCR rate in the standard arm.

Standard	Research	Total no. of patients
50%	70%	186
**55%**	**75%**	**178**
60%	80%	164

Based on the middle of the pCR value, a total of 632 patients: 178 gBRCA and 454 non-BRCA patients are required. Considering ~5% rate of non-evaluable patients, it is planned to recruit a total of **666** patients including **188** gBRCA patients between the control and the selected research arm in the phase II/III trial.

With an approximate 114 patients randomised to the dropped research arm until the end of Stage 2 (53 in the arm to be dropped and a further 61 recruited into the arm to be dropped while waiting for the results), the total sample size for the 3 stage phase II/III trial is therefore expected to be 666+114=**780.**

As of 12 October 2022, a total of 107 BRCA (14.12%) participants have been recruited into the trial. 115 patients were dropped in Arm 1. 22 of these have been gBRCA patients, and 93 were non-gBRCA patients. The recruitment figure minus Arm 1 is 643, of which 85 participants were gBRCA patients.

[redacted]

[redacted]

[redacted]

[redacted]

16.2 Statistical Analysis methods

16.2.1 Analysis population

Intent-to-treat population

The Intent-to-treat population is defined as all patients randomised in the trial, regardless of whether they actually received treatment. The treatment group will be analysed as randomised.

Safety population

The safety population compares all patients randomised and having received at least one dose of trial treatment. The treatment group will be analysed as treated.

16.2.2 Statistical Analysis

A detailed statistical analysis plan will be drafted before any analyses are performed. Briefly,

Stage 1: Safety

The primary outcome measure is safety in the research arms. The safety analysis for each of the two research arms will be performed when the first 25 patients in each arm who had received at least one dose of Olaparib Protocol treatment have completed their Protocol treatment. It is summarised as the percentage, together with 95% confidence interval of the grade ≥ 3 toxicities that appear to be associated with Olaparib. Should the modification of research regimen be required, the initial patients recruited will not be included in the final phase III primary efficacy analysis.

Stage 2: Selection of the research arm

The primary outcome measure is the pCR rate. The analysis will be performed when pCR is available for 53 patients in each of two research arms. First, the null hypothesis that the true pCR rate is 35% or less will be tested using a one-sided test at 0.05 level and then the arm for the phase III trial will be selected by comparing their observed pCR rates. While the decision of a research arm selected is based on efficacy (pCR) mainly, treatment compliance, safety and practical issues would also be considered.

Criteria of completion rate of Olaparib treatment as per Protocol

The completion rate of Olaparib treatment is a potential concern. If there are 25 or more out of the 53 patients completing the Olaparib treatment, the upper limit of the 95% confidence interval of completion rate is over 60%, this will be deemed acceptable. However, if there are 24 or less out of the 53 patients completing the Olaparib treatment, the research regimen might be dropped.

The results of analyses for the pCR rate, the completion rate of Olaparib Protocol treatment and safety for all three arms will be presented to the IDSMC. Following their review, a research regimen may be picked up as a winner based on the guidelines provided in the IDSMC charter.

Stage 3: Efficacy analysis

Stage 3: efficacy - Primary outcome measure is pCR, defined as absence of invasive disease in the breast and axillary nodes. The primary analysis for the primary outcome measure will be the standard chi-squared test to compare the pCR between the two arms.
The comparison between the two arms will be performed independently for TNBC non-gBRCA patients and for gBRCA patients. Secondary outcome measures: RFS, BCSS, Distant disease-free survival, Local recurrence-free survival, OS and TTSC will be analysed using Kaplan-Meier approaches and compared between the two arms in an exploratory manner using the standard log-rank test together with the hazard ratio (+95%CI) for TNBC non-gBRCA and for patients with gBRCA.
Results from exploratory objectives will not be included in the final trial report.

A joined data analysis will take place, after recruitment for the two cohorts' (TNBC, non-gBRCA and gBRCA patients) and the two independent analyses are complete.

[redacted]

16.2.3 Interim Analyses

An Independent Data and Safety Monitoring Committee (IDSMC) will review data, by treatment group, on the safety of patients in the trial at the end of stage I. The IDSMC will meet for the Safety Analysis and also annually until data are mature for the pCR outcome measure (primary outcome measure). A safety and data report will be created for each IDSMC meeting. Also, the proportion of patients with gBRCA will be reviewed to ensure that the required number of gBRCA patients will be recruited in the trial.

16.3 Futility Analysis

A futility analysis based on gBRCA1 and 2 and non-gBRCA patients will be considered at the corresponding iDSMC meeting as follows:
1) The trial is powered for these subgroups;
2) Unnecessary toxicity to patients may be avoided;
3) Significant costs could be saved and the trial maybe stopped early if there were no benefit.
The conditional power based on the target effect size method will be applied when there are over 76 gBRCA1/2 patients (40-50% of primary outcome data) and approx. 190 non-gBRCA patients with the primary outcome reported. Estimates of uncertainty (bootstrapping conditional powers) will be considered. As a guide to the IDSMC, the trial might stop early for futility if there were a low conditional power, for an example, ≤15%.

16.4 Criteria for the premature termination of the trial

Stopping guidelines will be discussed with the IDSMC and incorporated into their charter. They will monitor the progress of the trial regularly and if a difference emerges early, the trial will be discontinued in accordance with stopping rules. In order to reduce the chance of false positive or negative interim results signifying early stopping of the trial, small levels of significance need to be considered at any interim analysis time points. These will be detailed in the Statistical Analysis Plan which will be written in collaboration with the IDSMC.

As a guideline, early stopping for efficacy would only be recommended if the results were likely to convince the majority of clinicians that the addition of Olaparib should be introduced.

16.5 Definition of the end of the trial

For Regulatory purposes the end of the intervention period of the trial will be 30 days after the last patient completes surgery.

The non-interventional observation period of the trial will continue for at least 10 years following the completion of surgery of the last patient to have surgery (per the follow-up Protocol mentioned above).

All essential trial documents (including patient notes) must be retained for at least 5 years following the end of the trial. The trial Sponsor will notify the centres when trial documents may be destroyed.

17 Data handling and record keeping

17.1 Case Report Forms

All data will be transferred into a Case Report Form (CRF) which will be de-identified. All trial data in the CRF must be extracted from and be consistent with the relevant source documents. The CRFs must be completed, dated and signed by the Investigator or designee in a timely manner. It remains the responsibility of the Principal Investigator for the timing, completeness, legibility and accuracy of the CRF pages. The CRF will be accessible to trial coordinators, data manager, the Investigators, clinical trial monitors, auditors and inspectors as required.

Completed originals of the CRFs should be posted to the PARTNER office within a timely manner. The Investigators must ensure that the CRFs and other supporting trial related documentation are sent to the PARTNER office containing no patient identifiable data.

The Investigator will retain a copy of each completed CRF page at site. The investigator will also supply the PARTNER office with any required, de-identified background information from the medical records as required.

Case Report Forms will be designed by PARTNER Office.

CRF completion advice: All CRF pages should be clear, legible and completed in black ink. Errors should be crossed through with a single stroke so that the original entry can still be seen. Corrections should be inserted and the change dated and initialled by the Investigator or designee. If it is not clear why the change has been made, an explanation should be written next to the change. Typing fluid (Tipex) should not be used.

17.2 Data quality assurance & monitoring

Case Report Forms must be submitted to the PARTNER Office in a timely manner according to the recommended schedule. On receipt, all forms will be checked for completeness and congruity. Forms containing empty data fields or data anomalies will be queried and returned to site for resolution.

17.3 Source Data

To enable peer review, monitoring, audit and/or inspection the Investigator must agree to keep records of all participating patients (sufficient information to link records, e.g. CRFs, hospital records and samples), all original signed informed consent forms and copies of the CRF pages.

Source data for this trial includes: Informed Consent Forms, patient medical records, patient questionnaires, online test results, histopathology slides, print outs from medical assessments, e.g., ECG charts, CT scans histopathology reports.

17.4 Data Protection & Patient Confidentiality

All Investigators and trial site staff involved in this trial must comply with the requirements of the EU General Data Protection regulation, Data Protection Act 2018 and Trust Policy with regards to the collection, storage, processing and disclosure of personal information and will uphold the Act's core principles.

The personal data recorded on all documents will be regarded as strictly confidential. To preserve the patient's anonymity, only their initials and date of birth, along with their unique

trial number will be recorded on the CRFs. Patients should be assured that their confidentiality will be respected at all times.

18 Translational Studies and Sample collections

The primary hypothesis being tested within PARTNER translational sub-studies is that there are pharmacogenomic and proteomic markers that can be correlated with outcomes (pCR and RFS) in patients randomised to receive Olaparib versus those that do not. We will also investigate the differences in response between gBRCA patients and those in the various sub-types of basal TNBC and identify any potential related biomarkers. We will also investigate the potential role of the immune system in early stage TNBC.

The overall aim is to identify molecular markers that predict benefit (or lack thereof) of addition of Olaparib to conventional chemotherapy (in other words to discover the equivalent of the trastuzumab/HER2 paradigm). To address this aim we propose to do both pharmacogenetic and pharmacogenomic studies in all patients accrued into the trial.

The term pharmacogenomics is used more generally here to cover both the study of germline genetic variation and its influence in drug disposition, toxicity and survival and the study of genomic/transcriptomic variation in tumours and how this somatic cancer genomic/transcriptomic composition influences drug activity and the natural history of the tumour.

The samples collected will be profiled with the following specific aims:
Primary aims:

- To derive molecular signatures (from combined DNA/RNA/miRNA profiling) predictive of Olaparib response in fresh frozen biopsies and to validate these signatures using the nucleic acids and Tissue Micro Arrays from paraffin-embedded samples.
- To determine if circulating tumour DNA burden in gBRCA and TNBC can be used to track treatment response and disease relapse.
- To further elucidate the similarities between gBRCA and TNBC and additionally to investigate the molecular subtypes associated with TNBC.
- To identify and/or validate candidate pharmacogenomic markers that indicate an increased susceptibility to specific treatment related toxicities such as sensory peripheral neuropathy.
- To investigate the presence/absence of HRD-Loss of Heterozygosity type scores and genomic scars and their relationship to treatment response and BRCA mutation status. During this work we will consider various HRD type signatures.
- To investigate the distribution of the trial cohort within the 111 integrative cluster prognostic classification [87] or other appropriate prognostic / predictive classifications.
- PARTNER and [redacted] patients will be offered as part of the translational sub-studies the opportunity to have whole genome sequencing of their DNA (tumour and blood sample) and RNA sequencing (tumour only).
- To assess tumour mutation burden (TMB), molecular signatures and actionable mutations in pre-treatment tissue biopsy samples (using whole genome/exome sequencing or a tissue TMB assay).

Secondary aims:

- To validate anthracycline and taxane 'resistance' signatures derived by profiling tumours from NEAT, Neo-tAnGo, tAnGo, and ARTemis and to determine if tumours with such chemotherapy resistance signatures derive benefit from Olaparib.
- To determine whether molecular subtypes previously identified and validated have predictive value for Olaparib response (in other words, is there a subtype of breast cancer particularly 'sensitive' to Olaparib).
- To assess Olaparib homologous recombination repair mutation panel in pre-treatment tissue biopsies, assuming adequate availability of tissue.
- To identify and/or validate candidate pharmacogenetic markers that predict benefit (or lack thereof) from addition of Olaparib.

- To investigate the relationship between tumour infiltrating lymphocytes and other relevant immune biomarkers and treatment response or prognosis.
- To investigate pathway genes associated with DNA repair and determine their relationship to prognosis.
- To determine if circulating proteomic markers could be used to predict Olaparib benefit.
- To test the hypothesis that miRNAs are robust predictive markers of Olaparib response.
- To investigate epigenetics factors influencing treatment response and outcome.
- To investigate the potential for using organoids (as an in vitro model) and animal models such as patient derived tumour xenografts to mirror patient treatment and predict drug response/resistance.
- To investigate the potential for circulating tumour DNA and circulating tumour cells to predict for early relapse or be markers of response.
- To understand the role of the immune system in the response and outcome of gBRCA and TNBC patients. This will include investigating the tumour microbiome (e.g. stool samples) and peripheral blood mononuclear cells (PBMCs).
- To combine the results of PARTNER/*PartnerING* patient analysis of genomic, digital pathology, radiomic, metabolomic, epigenetic and clinical data to develop predictive and prognostic tools.
- To allow a greater understanding of the biology of these sub-groups of breast cancer de-identified summary results will be used in meta-analyses and shared with co-investigators in academia and industry, after discussion and agreement with the Chief Investigator and TSC.
- To understand, if we can predict for response to treatment earlier using multiple modalities of data generated from the PARTNER patients.

18.1 Mandatory blood samples for pharmacogenomic markers

This collection is mandatory and part of the clinical trial.

Two blood samples from all randomised patients are expected for DNA and protein isolation to validate candidate chemotherapy pharmacogenetic identified in Cambridge by PG-SNPs (CRUK TRICC-funded) and its associated studies.

- One mandatory blood sample, consisting of 2x9ml EDTA bottles, should be obtained from each patient pre-treatment.
- Another sample (2x9ml EDTA bottles) should also be obtained from each patient post-treatment.

It is recommended that the blood sample is obtained at the same time as routine blood samples are taken, to minimise the impact on the patient. Labelled bottles with the patient's unique PARTNER trial number, patient's date of birth and initials must be sent, in the appropriate packaging to the PARTNER Office upon request. Please refer to the laboratory manual for details and instructions.

18.2 Mandatory histopathology material and reports

This collection is mandatory and part of the clinical trial.*

Please be aware that it will be the responsibility of the local research team to obtain their patient's pathology material if the material is stored at a separate site to either the randomising hospital or the hospital of the named pathology contact.
Formalin-Fixed Paraffin-Embedded diagnostic tumour tissue and primary surgical tumour tissue, and also relapse tumour tissue block (as applicable) will be requested at appropriate

times along with corresponding diagnostic and primary surgical slides, from all randomised patients, for DNA/RNA/miRNA isolation and for Tissue Micro Array construction.

Diagnostic tumour block
Core biopsies will be performed on all patients to make the diagnosis of breast cancer. The patient's diagnostic paraffin embedded tumour tissue block will be requested from the relevant Pathology department.
If diagnostic material is urgently needed, please contact the PARTNER Office who will immediately retrieve the material and send it back to the Pathology department from whom it was requested.
It is important to highlight that cores will only be taken if there is enough material available, and that a sufficient amount of diagnostic material will remain for a representative sample of the disease (at least 3mm total invasive disease), because we understand that diagnostic material is the priority over research material.

Post cycle 6 biopsy (For PartnerING patients only).
Those patients that are included into PartnerING are required to have a post cycle 6 biopsy (at least two samples). Cores from the biopsy will be requested to be centrally assessed in order to confirm eligibility criteria for this pathway therapy (see section 10.5) and for collection of fresh tissue (see section 18.3).

Primary surgical tissue blocks
At primary surgery, pathologists should take several tumour samples for embedded paraffin tissue blocks. One tumour tissue block will be requested for the PARTNER trial.
Where a complete clinical or pathological response is observed, it is still requested that one slide from the original site of the tumour is taken and supplied to the PARTNER office on request.

Relapse tumour tissue blocks (when applicable)
When possible, relapse tumour tissue blocks and H&E slides should also be sent to the PARTNER office.

All diagnostic, surgical and relapse tissue blocks will be returned to the original pathology department after cores have been removed for research purposes.

Diagnostic biopsy, primary surgical and relapse histopathology reports
A copy of the associated **diagnostic biopsy, primary surgical and relapse reports** are requested for each patient as applicable, these should contain only the patient's unique PARTNER trial number with patient date of birth and initials. There will be central review (2 readers) of all primary surgery histopathology reports for the primary endpoint of pathological complete response (pCR).

Diagnostic and primary surgery H&E (Haematoxylin and Eosin) slides
H&E slides from all blocks taken at diagnosis and primary surgery (tumour and lymph nodes where applicable) will be requested retrospectively from the nominated pathologist for all patients participating in the trial. Where slides are unavailable, or cannot be made by the site, then blocks may be sent to the PARTNER Office, and slides will be made. There will be central pathological review of all patients who have not had a pathological Complete Response for the secondary endpoint of Residual Burden Disease. Our central pathologists will compare the diagnostic slides with the surgical slides provided. All grades of response (see section 15.1 for score) will be defined for each patient by this central review. Cellularity will be compared with the original diagnostic slides taken prior to trial treatment.
As per diagnostic slide, see section 10.4.2, TILs will be centrally scored.

* The collection of mandatory histopathology material and reports is also applicable for those patients who are participating in the PartnerING trial.

18.3 Fresh Tissue samples collection

PARTNER:
The collection of Fresh tissue in the PARTNER main trial is optional. Patients will give written permission for extra biopsies to be taken via the **PARTNER Fresh tissue study consent form, consent 3, and on-going consent will be confirmed verbally prior to each procedure**.
It is our strong preference to obtain fresh tissue whenever possible for DNA/RNA/miRNA isolation. Ideally:

- One sample of fresh tumour tissue at diagnosis or prior to start treatment.
- If possible, one fresh tumour tissue biopsy at mid-treatment / after 4 cycles of chemotherapy.
- One sample of fresh tumour tissue after cycle 7 / post-chemotherapy or during surgery.
- If possible an additional tissue sample collected from the metastatic or relapsed site(s) at the time of relapse as applicable.

[redacted]

It is recommended that the tissue is obtained by ultrasound guided biopsy and/or during surgery to minimise the impact on the patient. The sample must be immediately immersed in a solution of 'RNAlater' (or equivalent) or in liquid nitrogen.
The samples must be labelled with patient trial number, date of birth and initials and subsequently couriered to the PARTNER Office.
Further information can be found in the Laboratory Manual.

18.4 Sequential blood samples

PARTNER:
The collection of sequential blood samples in the PARTNER main trial is **optional.** Patients will give permission for extra blood samples to be taken via the PARTNER Sequential Blood study consent form and on-going consent will be confirmed verbally prior to each procedure.

Patients will be asked for 1 extra blood sample (up to 22.5ml) to be taken for research purposes at each of the following time points:
- prior to treatment cycle 1,
- before cycle 2,
- before cycle 5,
- after cycle 7 but prior to surgery,
- whenever possible, a blood sample is to be collected immediately after surgery,
- approximately 2-4 weeks after surgery. Ideally to coincide with local surgical review,
- approximately 6 months after surgery (6 month f/u visit),
- approximately 12 months after the surgery (year 1 f/u visit),
- at each follow-up visit for 5 years (year 2, 3, 4 and 5),
- at relapse if applicable.

Along with these samples, a pre-treatment (9ml), a post-treatment (9ml) and 12 months follow-up (9ml) samples must be collected for level of **Anti-Mullerian Hormones** and other proteins. Therefore, on these occasions a total of up to 31.5ml will be collected.

[redacted]

It is recommended that the blood sample is obtained at the same time as routine bloods samples are taken, to minimise the impact on the patient.
Sites will require the availability of a centrifuge, and preferably a -70 °C /-80 °C freezer, however a -20°C freezer can be used for sample storage. Please refer to the laboratory manual for freezer specifications. The samples must be labelled with patient trial number, date of birth and initials before being frozen and subsequently couriered to the PARTNER Office.

Further information can be found in the laboratory manual.

18.5 Optional sequential microbiome samples sub-study

This observational prospective sub-study aims to collect commensal and tumour microbiome samples from patients enrolled to the PARTNER trial (before, during, and after completion of treatment). In view of the current literature, our hypothesis is that the microbiome of excellent responders to PARTNER treatment is different from the microbiome of non-responders. Understanding the similarities and differences of the microbiome in responders and poor or non-responders will allow the development of future interventions such as microbiome manipulation or microbiome-based personalized treatment selection.
Commensal microbiome samples will be taken non-invasively from various locations: mouth, urine and stool. Please refer to the laboratory manual for collection methods. Patients will give permission for extra microbiome samples to be taken via the PARTNER Microbiome sub-study consent form.
Patients will be asked for oral swab, urine and stool collection to be taken for research purposes at each of the following time points:

- prior to treatment cycle 1,
- before cycle 5,
- after cycle 7 but prior to surgery,
- approximately 6 months after surgery,
- at relapse if applicable.

Sites will require the availability of a centrifuge, and preferably a -70 °C /-80 °C freezer. Please refer to the laboratory manual for freezer specifications.
The samples must be labelled with patient trial number, date of birth and initials before being frozen and subsequently couriered to the PARTNER Office or to associated laboratories.

Further information can be found in the Laboratory Manual.

19 Trial conduct

19.1 Conduct of trial

The Principal Investigator at each participating site is required to supply the PARTNER Office with a current curriculum vitae and evidence of up-to-date GCP training before the trial is opened at the applicable site. They will also be required to complete a signature page for each new version of the PARTNER Protocol issued by the PARTNER office.
All site personnel involved in the conduct of the trial (at a minimum the Principal Investigator, a pharmacist, a pathologist or tissue bank staff, and a lead nurse or coordinator or data manager for the trial) will be asked to complete:

- Registration Forms,
- staff Signature and Delegation Logs,
- attend an initiation meeting which will cover trial rationale, Protocol procedures, and collection and reporting of data.

All staff involved in the trial should be listed on the delegation log and should have up to date training records (including GCP) available on request. Following this, all sites will be provided with an Investigator Site File and Pharmacy Site File containing instructional materials and documentation required for the conduct of the trial. The PARTNER office will offer continued support and training as necessary for all sites via telephone, email, and mail. New site staff who did not complete initiation training will be offered initiation training by the PARTNER office, otherwise site staff present at the initiation training are able to train their new staff, and the trial initiation slides can be provided to sites for this purpose.

19.2 Trial Committees

19.2.1 Trial Management Group (TMG)

The Trial Management Group (TMG) are responsible for the day-to-day running of the trial. They will meet regularly to oversee the running of the trial and review serious adverse events (SAEs) which have occurred in the trial. If there are specific safety concerns these will be raised with the TSC and/or the IDSMC.
They will also prepare reports for the IDSMC and TSC, including interim analysis.

19.2.2 Independent Data and Safety Monitoring Committee (IDSMC)

The Independent Data and Safety Monitoring Committee (IDSMC) is independent of Investigators and the TMG. The group will meet after stage 1 for safety interim analysis and after stage 2 for selection of the research arm. They will also meet approx. annually until data are mature for the pCR outcome measure (primary endpoint). The IDSMC will review reports from the TMG and give advice on continuing recruitment. There are no formal stopping rules for efficacy (see section 16.3). A recommendation to discontinue recruitment (in all patients or in selected subgroups) will be made only if the emerging safety data indicate that the safety of the patients is not maintained. If a decision is made to continue, the IDSMC will advise on the frequency of future reviews of the data on the basis of accrual and event rates. The IDSMC will make recommendations to the TSC as to the continuation of the trial.

19.2.3 Trial Steering Committee (TSC)

The Trial Steering Committee (TSC) includes members who are independent of the trial Investigators and TMG. It will provide overall supervision of the trial. It will meet at approx. annual intervals and will receive reports from the TMG and IDSMC.

19.3 Relationship between Trial Committees

The relationships between the various trial committees are displayed in the diagram below (Figure 2). Charters will be developed for all committees.

Figure 2: Diagram of Relationships between Trial Committees

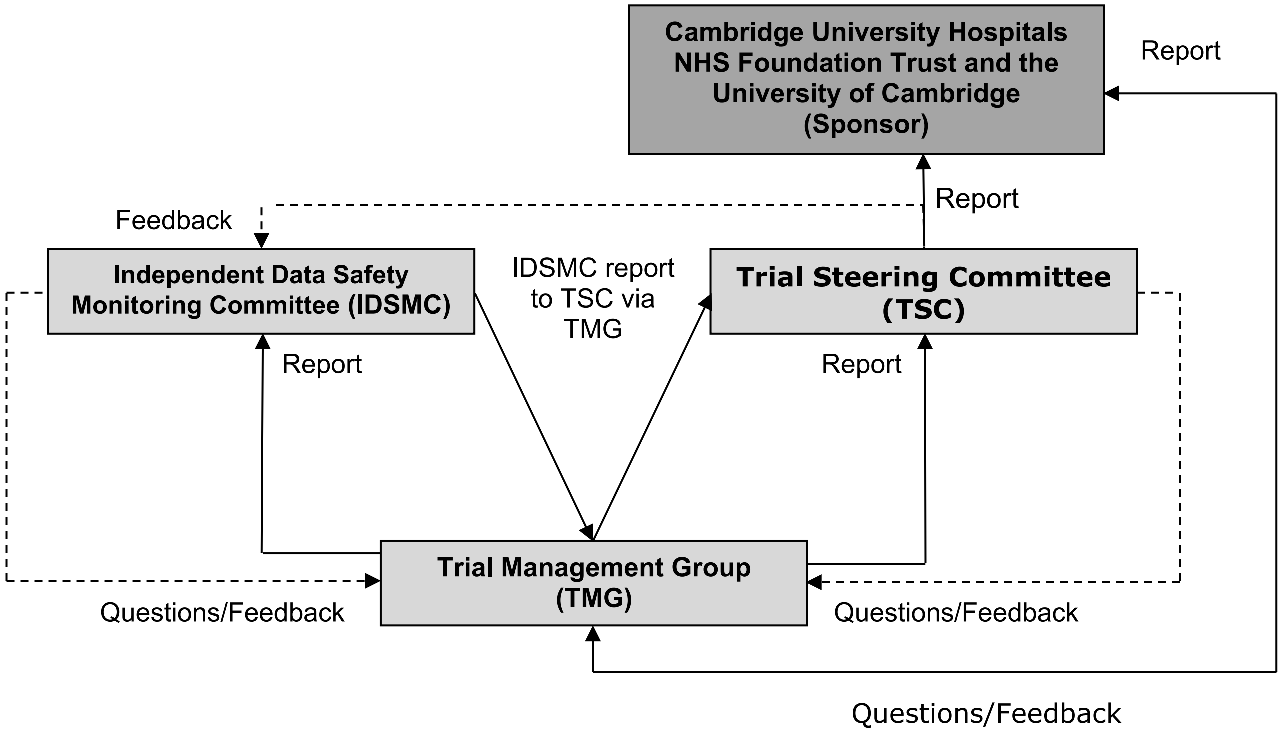

20 Ethical & Regulatory considerations

20.1 Consent

The Informed Consent Form must be approved by the REC and must be in compliance with the principles of GCP, regulatory requirements and legal requirements. The Investigator or designee must ensure that each trial participant is fully informed about the nature and objectives of the trial and possible risks associated with their participation.

The Investigator or designee will obtain written informed consent from each patient before any trial-specific activity is performed. The informed consent form used for this trial and any change made during the course of this trial, must be prospectively approved by the REC. The Investigator will retain the original of each patients signed informed consent form.

Should a patient require a translation of the trial documentation by a locally approved interpreter/translator, it is the responsibility of the individual Investigator to use locally approved translators.

Any new information which becomes available, which might affect the patient's willingness to continue participating in the trial will be communicated to the patient as soon as possible.

20.2 Ethical committee review

Before the start of the trial or implementation of any amendment, approval of the trial Protocol, Protocol amendments, Informed Consent Forms and other relevant documents, e.g. GP information letters, will be obtained from the REC. All correspondence with the REC will be retained in the Trial Master File/Investigator Site File.

Annual reports will be submitted to the REC in accordance with national requirements. It is the Chief Investigators' responsibility to produce the annual reports as required.

20.3 Regulatory Compliance

The trial will not commence until a Clinical Trial Authorisation is obtained from the Competent Authority or Regulatory Agency. The Protocol and trial conduct will comply with the Medicines for Human Use (Clinical Trials) Regulations 2004 and any relevant amendments.

Annual Reports will be submitted to the Competent Authority or Regulatory Agency in accordance with national requirements. It is the Chief Investigator's responsibility to produce the annual reports as required.

20.4 Protocol Amendments

Protocol Amendments must be reviewed and agreement received from the Sponsor for all proposed Amendments prior to submission to the Health Research Agency (for the UK only), REC and/or Competent Authority, or implementation (in the case of non-substantial amendments).

The only circumstance in which an Amendment may be initiated prior to the HRA, REC and/or Competent Authority or Regulatory Agency approval is where the change is necessary to eliminate apparent, immediate risks to the patients (Urgent Safety Measures). In the case, accrual of new patients will be halted until the HRA, REC and/or Competent Authority or Regulatory Agency approval has been obtained.

In the event of an urgent safety measure the Chief Investigator (or suitably qualified and delegated co-Investigator) will cascade the information verbally and by e-mail to staff directly involved in patient care (e.g. research nurse and co-Investigators).

20.5 Peer Review

Peer reviews were undertaken by UK National Cancer Research Institute, Breast Cancer Clinical Studies Group and international experts on behalf of CRUK.

20.6 Declaration of Helsinki and Good Clinical Practise

The trial will be performed in accordance with the spirit and the letter of the declaration of Helsinki, the conditions and principles of GCP, the Protocol and applicable regulatory requirements and laws.

20.7 GCP Training

All trial staff must hold evidence of appropriate GCP training or undergo GCP training prior to undertaking any responsibilities on this trial. This training should be updated every 2 years or in accordance with the participating sites local practice. It is the responsibility of the PI to ensure all staff participating in the trial, have an updated GCP certificate.

20.8 Sponsorship, Financial and Insurance

The trial will be sponsored by Cambridge University Hospitals NHS Foundation Trust and the University of Cambridge. The trial will be funded in part by a project grant from AstraZeneca who will also supply Olaparib.

Cambridge University Hospitals NHS Foundation Trust, as a member of the NHS Clinical Negligence Scheme for Trusts, will accept full financial liability for harm caused to participants in the clinical trial caused through the negligence of its employees and honorary contract holders. There are no specific arrangements for compensation should a participant be harmed through participation in the trial, but no-one has acted negligently.

The University of Cambridge will arrange insurance for negligent harm caused as a result of Protocol design and for non-negligent harm arising through participation in the clinical trial.

20.9 Monitoring, Audit & Inspection

The Investigator must make all trial documentation and related records available should a Competent Authority or Regulatory Agency Inspection occur. Should a monitoring visit or audit be requested, the Investigator must make the trial documentation and source data available to the Sponsor’s representative. All patient data must be handled and treated confidentially.

The Sponsor’s monitoring frequency will be determined by an initial risk assessment performed prior to the start of the trial. A detailed monitoring plan will be generated detailing the frequency and scope of the monitoring for the trial. The monitoring plan will include aspects of remote monitoring of sites. Sites will be responsible for completing and returning the remote monitoring form to the PARTNER Office, and acting upon any required actions. Throughout the course of the trial, the risk assessment will be reviewed and the monitoring frequency adjusted as necessary.

20.10 Protocol Compliance and Breaches of GCP

Prospective, planned deviations or waivers to the Protocol are not allowed under the UK regulations on Clinical Trials and must not be used.

Protocol deviations, non-compliances, or breaches are departures from the approved Protocol. They can happen at any time, but are not planned. They must be reported to the PARTNER office and adequately documented on the relevant forms.

Deviations from the Protocol which are found to occur constantly again and again will not be accepted and will require immediate action and could potentially be classified as a serious breach.

Any potential/suspected serious breaches of GCP must be reported immediately to the Sponsor without any delay.

20.11 Publications policy

Ownership of the data arising from this trial resides with the TMG. On completion of the trial the data will be analysed and tabulated and a Final Trial Report prepared.
The main trial results will be presented at national and international conferences and published in a peer-reviewed journal, on behalf of all collaborators. All presentations and publications relating to the trial must be authorised by the TMG.

The manuscript will be prepared by a writing group, appointed from amongst the TMG and high-accruing or key Investigators. The PARTNER Office and all participating centres and Investigators, as well as the CCTU and AstraZeneca will be acknowledged in this publication. The first and last authorship of the main trial publication will be given to the trial Chief Investigator and staff at the PARTNER Office. Other authorship will be given to Investigators ordered by recruitment or dependent on their contribution toward the development and conduct of the trial.

21 References

1. Collaborative Group on Hormonal Factors in Breast Cancer. Breast cancer and breastfeeding: collaborative reanalysis of individual data from 47 epidemiological studies in 30 countries, including 50302 women with breast cancer and 96973 women without the disease. *Lancet (London, England)* 2002; **360**:187–95.
2. Cancer Research UK. Breast Cancer Statistics 2012. *Cancer Res UK (2012), Breast Cancer Stat Cancer Res UK* 2012.
3. Dawson S-J, Rueda OM, Aparicio S, Caldas C. A new genome-driven integrated classification of breast cancer and its implications. *EMBO J* 2013; **32**:617–628.
4. Foulkes WD, Smith IE, Reis-Filho JS. Triple-Negative Breast Cancer. *N Engl J Med* 2010; **363**:1938–1948.
5. Liedtke C, Mazouni C, Hess KR, *et al.* Response to Neoadjuvant Therapy and Long-Term Survival in Patients With Triple-Negative Breast Cancer. *J Clin Oncol* 2008; **26**:1275–1281.
6. Lehmann BD, Bauer JA, Chen X, *et al.* Identification of human triple-negative breast cancer subtypes and preclinical models for selection of targeted therapies. *J Clin Invest* 2011; **121**:2750–2767.
7. Masuda H, Baggerly KA, Wang Y, *et al.* Differential Response to Neoadjuvant Chemotherapy Among 7 Triple-Negative Breast Cancer Molecular Subtypes. *Clin Cancer Res* 2013; **19**:5533–5540.
8. Robertson L, Hanson H, Seal S, *et al.* BRCA1 testing should be offered to individuals with triple-negative breast cancer diagnosed below 50 years. *Br J Cancer* 2012; **106**:1234–8.
9. Evans DG, Howell A, Ward D, Lalloo F, Jones JL, Eccles DM. Prevalence of BRCA1 and BRCA2 mutations in triple negative breast cancer. *J Med Genet* 2011; **48**:520–522.
10. Roy R, Chun J, Powell SN. BRCA1 and BRCA2: important differences with common interests. *Nat Rev Cancer* 2012; **12**:372–372.
11. Antoniou A, Pharoah PDP, Narod S, *et al.* Average Risks of Breast and Ovarian Cancer Associated with BRCA1 or BRCA2 Mutations Detected in Case Series Unselected for Family History: A Combined Analysis of 22 Studies. *Am J Hum Genet* 2003; **72**:1117–1130.
12. Mavaddat N, Barrowdale D, Andrulis IL, *et al.* Pathology of Breast and Ovarian Cancers among BRCA1 and BRCA2 Mutation Carriers: Results from the Consortium of Investigators of Modifiers of BRCA1/2 (CIMBA). *Cancer Epidemiol Biomarkers Prev* 2012; **21**:134–147.
13. Augustyn AM, Agostino NM, Namey TL, Nair S, Martino MA. Two patients with germline mutations in both BRCA1 and BRCA2 discovered unintentionally: a case series and discussion of BRCA testing modalities. *Breast Cancer Res Treat* 2011; **129**:629–634.
14. Huzarski T, Byrski T, Gronwald J, *et al.* Ten-year survival in patients with BRCA1-negative and BRCA1-positive breast cancer. *J Clin Oncol* 2013; **31**:3191–6.
15. Mauri D, Pavlidis N, Ioannidis JPA. Neoadjuvant Versus Adjuvant Systemic Treatment in Breast Cancer: A Meta-Analysis. *JNCI J Natl Cancer Inst* 2005; **97**:188–194.
16. Cortazar P, Zhang L, Untch M, *et al.* Pathological complete response and long-term clinical benefit in breast cancer: The CTNeoBC pooled analysis. *Lancet* 2014; **384**:164–172.
17. Prowell TM, Pazdur R. Pathological Complete Response and Accelerated Drug Approval in Early Breast Cancer. *N Engl J Med* 2012; **366**:2438–2441.
18. Hatzis C, Gould R, Zhang Y, *et al.* Abstract P6-06-37: Predicting improvements in survival based on improvements in pathologic response rate to neoadjuvant chemotherapy in different breast cancer subtypes. *Cancer Res* 2013; **73**:P6-6-37-P6-06–37.
19. Byrski T, Gronwald J, Huzarski T, *et al.* Pathologic complete response rates in young women with BRCA1-positive breast cancers after neoadjuvant chemotherapy. *J Clin Oncol* 2010; **28**:375–379.
20. von Minckwitz G, Schneeweiss A, Loibl S, *et al.* Neoadjuvant Carboplatinin patients with triple-negative and HER2-positive early breast cancer (GeparSixto; GBG 66): a randomised phase 2 trial. *Lancet Oncol* 2014; **15**:747–756.
21. Sikov WM, Berry DA, Perou CM, *et al.* Impact of the addition of Carboplatinand/or bevacizumab to neoadjuvant once-per-week Paclitaxelfollowed by dose-dense doxorubicin and cyclophosphamide on pathologic complete response rates in stage II to III triple-negative breast cancer: CALGB 40603 (A. *J Clin Oncol* 2015; **33**:13–21.
22. Gunter Von Minckwitz, Eric Hahnen, Peter A. Fasching, Jan Hauke, Andreas Schneeweiss, Christoph Salat, Mahdi Rezai, Jens U. Blohmer, Dirk Michael Zahm, Christian Jackisch, Bernd Gerber, Peter Klare, Sherko Kummel, Holger Eidtmann, Stefan Paepke, Valentina and G and A-BSG. Pathological complete response (pCR) rates after Carboplatin-containing neoadjuvant chemotherapy in patients with germline BRCA (gBRCA) mutation and triple-negative breast cancer (TNBC): Results from GeparSixto. *J Clin Oncol* 2014; **32:15_supp**:1005–1005.
23. Hook J, Freeman S, P. Stenning S, *et al. GCIG ICON8 STAGE IA ANALYSIS: SAFETY AND FEASIBILITY OF TWO DOSE-FRACTIONATED CARBOPLATIN-PACLITAXELREGIMENS FOR THE FIRST-LINE TREATMENT OF OVARIAN CANCER*.; 2013.
24. Rugo H, Olopade O, DeMichele A, *et al.* Abstract S5-02: Veliparib/Carboplatinplus standard neoadjuvant therapy for high-risk breast cancer: First efficacy results from the I-SPY 2 TRIAL: *Cancer Res* 2013; **73**:S5-2-S5-2.
25. Dent RA, Lindeman GJ, Clemons M, *et al.* Phase I trial of the oral PARP inhibitor Olaparib in combination with Paclitaxelfor first- or second-line treatment of patients with metastatic triple-negative breast cancer. *Breast Cancer Res* 2013; **15**:R88.
26. Von Minckwitz G, Untch M, N??esch E, *et al.* Impact of treatment characteristics on response of different breast cancer phenotypes: Pooled analysis of the German neo-adjuvant chemotherapy trials. *Breast Cancer Res Treat* 2011; **125**:145–156.
27. Silver DP, Richardson AL, Eklund AC, *et al.* Efficacy of Neoadjuvant Cisplatin in Triple-Negative Breast Cancer. *J Clin Oncol* 2010; **28**:1145–1153.
28. Sikov WM, Dizon DS, Strenger R, *et al.* Frequent Pathologic Complete Responses in Aggressive Stages II to III Breast Cancers With Every-4-Week Carboplatinand Weekly PaclitaxelWith or Without Trastuzumab: A Brown University Oncology Group Study. *J Clin Oncol* 2009; **27**:4693–4700.
29. Chen XS, Nie XQ, Chen CM, *et al.* Weekly Paclitaxelplus Carboplatinis an effective nonanthracycline-containing regimen as neoadjuvant chemotherapy for breast cancer. *Ann Oncol* 2010; **21**:961–967.
30. Peintinger F, Sinn B, Hatzis C, *et al.* Reproducibility of residual cancer burden for prognostic assessment of breast cancer after neoadjuvant chemotherapy. *Mod Pathol* 2015; **28**:913–920.

31. Roy V, Pockaj BA, Northfelt DW, *et al.* N0338 phase II trial of docetaxel and Carboplatinadministered every two weeks as induction therapy for stage II or III breast cancer. *J Clin Oncol* 2008; **26**:563–563.
32. Chang HR, Glaspy J, Allison MA, *et al.* Differential response of triple-negative breast cancer to a docetaxel and Carboplatin-based neoadjuvant treatment. *Cancer* 2010; **116**:4227–4237.
33. von Minckwitz G, Martin M. Neoadjuvant treatments for triple-negative breast cancer (TNBC). *Ann Oncol* 2012; **23**:vi35-vi39.
34. Tutt A, Robson M, Garber JE, *et al.* Oral poly(ADP-ribose) polymerase inhibitor Olaparib in patients with BRCA1 or BRCA2 mutations and advanced breast cancer: a proof-of-concept trial. *Lancet* 2010; **376**:235–244.
35. Rivkin SE, Iriarte D, Sloan H, Wiseman C, Moon J. Phase Ib/II with expansion of patients at the MTD study of Olaparib plus weekly (metronomic) Carboplatinand Paclitaxelin relapsed ovarian cancer patients. *J Clin Oncol* 2014; **32**:5527.
36. Evers B, Drost R, Schut E, *et al.* Selective Inhibition of BRCA2-Deficient Mammary Tumor Cell Growth by AZD2281 and Cisplatin. *Clin Cancer Res* 2008; **14**:3916–3925.
37. Oplustilova L, Wolanin K, Mistrik M, *et al.* Evaluation of candidate biomarkers to predict cancer cell sensitivity or resistance to PARP-1 inhibitor treatment. *Cell Cycle* 2012; **11**:3837–3850.
38. Anon. The Genetic Testing in Epithelial Ovarian Cancer (GTEOC) Study: Direct access to BRCA1/2 genetic testing in oncology.
39. Kuroi K, Toi M, Ohno S, *et al.* Prognostic significance of subtype and pathologic response in operable breast cancer; a pooled analysis of prospective neoadjuvant studies of JBCRG. *Breast Cancer* 2015; **22**:486–495.
40. Von Minckwitz G, Untch M, Blohmer JU, *et al.* Definition and impact of pathologic complete response on prognosis after neoadjuvant chemotherapy in various intrinsic breast cancer subtypes. *J Clin Oncol* 2012; **30**:1796–1804.
41. Bonnefoi H, Litire S, Piccart M, *et al.* Pathological complete response after neoadjuvant chemotherapy is an independent predictive factor irrespective of simplified breast cancer intrinsic subtypes: A landmark and two-step approach analyses from the EORTC 10994/BIG 1-00 phase III trial. *Ann Oncol* 2014; **25**:1128–1136.
42. Symmans WF, Peintinger F, Hatzis C, *et al.* Measurement of residual breast cancer burden to predict survival after neoadjuvant chemotherapy. *J Clin Oncol* 2007; **25**:4414–4422.
43. Promberger R, Dubsky P, Mittlb??ck M, *et al.* Postoperative CMF does not ameliorate poor outcomes in women with residual invasive breast cancer after neoadjuvant epirubicin/docetaxel chemotherapy. *Clin Breast Cancer* 2015; **15**:505–511.
44. Masuda N, Lee S-J, Ohtani S, *et al.* Adjuvant Capecitabine for Breast Cancer after Preoperative Chemotherapy. *N Engl J Med* 2017; **376**:2147–2159.
45. Sundar R, Brown J, Ingles Russo A, Yap TA. Targeting ATR in cancer medicine. *Curr Probl Cancer* 2017; **41**:302–315.
46. Carrassa L, Damia G. Unleashing Chk1 in cancer therapy. *Cell Cycle* 2011; **10**:2121–2128.
47. Sørensen CS, Syljuåsen RG. Safeguarding genome integrity: The checkpoint kinases ATR, CHK1 and WEE1 restrain CDK activity during normal DNA replication. *Nucleic Acids Res* 2012; **40**:477–486.
48. Reaper PM, Griffiths MR, Long JM, *et al.* Selective killing of ATM- or p53-deficient cancer cells through inhibition of ATR. *Nat Chem Biol* 2011; **7**:428–430.
49. Karnitz LM, Zou L. Molecular pathways: Targeting ATR in cancer therapy. *Clin Cancer Res* 2015; **21**:4780–4785.
50. Carrassa L, Damia G. DNA damage response inhibitors: Mechanisms and potential applications in cancer therapy. *Cancer Treat Rev* 2017; **60**:139–151.
51. Ruzankina Y, Schoppy DW, Asare A, Clark CE, Vonderheide RH, Brown EJ. Tissue regenerative delays and synthetic lethality in adult mice after combined deletion of Atr and Trp53. *Nat Genet* 2009; **41**:1144–1149.
52. Ali R, Rakha EA, Madhusudan S, Bryant HE. DNA damage repair in breast cancer and its therapeutic implications. *Pathology* 2017; **49**:156–165.
53. Sultana R, Abdel-Fatah T, Abbotts R, *et al.* Targeting XRCC1 deficiency in breast cancer for personalized therapy. *Cancer Res* 2013; **73**:1621–1634.
54. Mohni KN, Kavanaugh GM, Cortez D. ATR pathway inhibition is synthetically lethal in cancer cells with ercc1 deficiency. *Cancer Res* 2014; **74**:2835–2845.
55. Keir ME, Butte MJ, Freeman GJ, Sharpe AH. PD-1 and Its Ligands in Tolerance and Immunity. *Annu Rev Immunol* 2008; **26**:677–704.
56. Vinay DS, Ryan EP, Pawelec G, *et al.* Immune evasion in cancer: Mechanistic basis and therapeutic strategies. *Semin Cancer Biol* 2015; **35**:S185–S198.
57. Pardoll DM. The blockade of immune checkpoints in cancer immunotherapy. *Nat Rev Cancer* 2012; **12**:252–64.
58. Bertucci F, Finetti P, Birnbaum D, Mamessier E. The PD1/PDL1 axis, a promising therapeutic target in aggressive breast cancers. *Oncoimmunology* 2016; **5**:e1085148.
59. Gaillard SL, Secord AA, Monk B. The role of immune checkpoint inhibition in the treatment of ovarian cancer. *Gynecol Oncol Res Pract* 2016; **3**:11.
60. Dréan A, Lord CJ, Ashworth A. PARP inhibitor combination therapy. *Crit Rev Oncol Hematol* 2016; **108**:73–85.
61. Denkert C, von Minckwitz G, Darb-Esfahani S, *et al.* Evaluation of tumor-infiltrating lymphocytes (TILs) as predictive and prognostic biomarker in different subtypes of breast cancer treated with neoadjuvant therapy - A metaanalysis of 3771 patients. *Cancer Res* 2017; **77**.
62. Mittendorf EA, Philips A V., Meric-Bernstam F, *et al.* PD-L1 Expression in Triple-Negative Breast Cancer. *Cancer Immunol Res* 2014; **2**:361–370.
63. Wimberly H, Brown JR, Schalper K, *et al.* PD-L1 Expression Correlates with Tumor-Infiltrating Lymphocytes and Response to Neoadjuvant Chemotherapy in Breast Cancer. *Cancer Immunol Res* 2015; **3**:326–332.
64. Nanda R, Chow LQM, Dees EC, *et al.* Pembrolizumab in patients with advanced triple-negative breast cancer: Phase Ib keynote-012 study. *J Clin Oncol* 2016; **34**:2460–2467.
65. Schmid P, Park YH, Muñoz-Couselo E, *et al.* Pembrolizumab (pembro) + chemotherapy (chemo) as neoadjuvant treatment for triple negative breast cancer (TNBC): Preliminary results from KEYNOTE-173. *J Clin Oncol* 2017; **35**:556.
66. Loibl S. 221TiP - A randomized phase II study to investigate the addition of PD-L1 antibody MEDI4673 (Durvalumab) to a taxane-anthracycline containing chemotherapy in triple negative breast cancer (GeparNuevo). *ESMO Annu Meet* 2016.
67. Pusztai L, Silber A, Hofstatter EW, *et al.* Safety of MEDI4736 (anti-PD-L1 antibody) administered concomitant with weekly nab-Paclitaxeland dose dense doxorubicin/cyclophosphamide (ddAC) as neoadjuvant chemotherapy for stage I-III triple negative breast cancer (TNBC): A Phase I/II trial. *J Clin Oncol* 2017; **35**:572.

68. Turner NC, Lord CJ, Iorns E, *et al.* A synthetic lethal siRNA screen identifying genes mediating sensitivity to a PARP inhibitor. *EMBO J* 2008; **27**:1368–1377.
69. Yazinski SA, Comaills V, Buisson R, *et al.* ATR inhibition disrupts rewired homologous recombination and fork protection pathways in PARP inhibitor-resistant BRCA-deficient cancer cells. *Genes Dev* 2017; **31**:318–332.
70. Yap TA, Sandhu SK, Carden CP, de Bono JS. Poly(ADP-Ribose) polymerase (PARP) inhibitors: Exploiting a synthetic lethal strategy in the clinic. *CA Cancer J Clin* 2011; **61**:31–49.
71. Yap TA, Krebs MG, Postel-Vinay S, *et al.* Phase I modular study of AZD6738, a novel oral, potent and selective ataxia telangiectasia Rad3-related (ATR) inhibitor in combination (combo) with Carboplatin, Olaparib or Durvalumab in patients (pts) with advanced cancers. *Eur J Cancer* 2016; **69**:S2.
72. Jiao S, Xia W, Yamaguchi H, *et al.* PARP inhibitor upregulates PD-L1 expression and enhances cancer-associated immunosuppression. *Clin Cancer Res* 2017; **23**:3711–3720.
73. Parkes EE, Walker SM, Taggart LE, *et al.* Activation of STING-dependent innate immune signaling by s-phase-specific DNA damage in breast cancer. *J Natl Cancer Inst* 2017; **109**:1–10.
74. Symmans WF, Wei C, Gould R, *et al.* Long-term prognostic risk after neoadjuvant chemotherapy associated with residual cancer burden and breast cancer subtype. *J Clin Oncol* 2017; **35**:1049–1060.
75. The Criteria Committee of the New York Heart, Association. *Nomenclature and Criteria for Diagnosis of Diseases of the Heart and Great Vessels*.; 1994.
76. Denkert C, Von Minckwitz G, Brase JC, *et al.* Tumor-infiltrating lymphocytes and response to neoadjuvant chemotherapy with or without Carboplatinin human epidermal growth factor receptor 2-positive and triple-negative primary breast cancers. *J Clin Oncol* 2015; **33**:983–991.
77. Salgado R, Denkert C, Demaria S, *et al.* The evaluation of tumor-infiltrating lymphocytes (TILs) in breast cancer: recommendations by an International TILs Working Group 2014. *Ann Oncol* 2015; **26**:259–271.
78. Blows FM, Driver KE, Schmidt MK, *et al.* Subtyping of Breast Cancer by Immunohistochemistry to Investigate a Relationship between Subtype and Short and Long Term Survival: A Collaborative Analysis of Data for 10,159 Cases from 12 Studies. Marincola FM, ed. *PLoS Med* 2010; **7**:e1000279.
79. Lehmann BD, Bauer JA, Schafer JM, *et al.* PIK3CA mutations in androgen receptor-positive triple negative breast cancer confer sensitivity to the combination of PI3K and androgen receptor inhibitors. *Breast Cancer Res* 2014; **16**:406.
80. Lal LS, Gerber DL, Lau J, Dana W. Retrospective evaluation of weekly Paclitaxelhypersensitivity reactions reported utilizing an electronic medical record system at a tertiary cancer center. *Support Care Cancer* 2009; **17**:1311–1315.
81. Calvert AH, Newell DR, Gumbrell LA, *et al.* Carboplatindosage: prospective evaluation of a simple formula based on renal function. *J Clin Oncol* 1989; **7**:1748–1756.
82. Ainsworth NL, Marshall A, Hatcher H, Whitehead L, Whitfield GA, Earl HM. Evaluation of glomerular filtration rate estimation by Cockcroft-Gault, Jelliffe, Wright and Modification of Diet in Renal Disease (MDRD) formulae in oncology patients. *Ann Oncol* 2012; **23**:1845–1853.
83. Sehouli J, Stengel D, Mustea A, *et al.* Weekly Paclitaxeland Carboplatin(PC-W) for patients with primary advanced ovarian cancer: results of a multicenter phase-II study of the NOGGO. *Cancer Chemother Pharmacol* 2007; **61**:243–250.
84. Dash N, Chafin SH, Johnson RR, Contractor FM. Usefulness of tissue marker clips in patients undergoing neoadjuvant chemotherapy for breast cancer. *Am J Roentgenol* 1999; **173**:911–917.
85. Pinder SE, Provenzano E, Earl H, Ellis IO. Laboratory handling and histology reporting of breast specimens from patients who have received neoadjuvant chemotherapy. *Histopathology* 2007; **50**:409–417.
86. Kern P, Kalisch A, Kolberg HC, *et al.* Neoadjuvant, anthracycline-free chemotherapy with Carboplatinand docetaxel in triple-negative, early-stage breast cancer: A multicentric analysis of feasibility and rates of pathologic complete response. *Chemotherapy* 2014; **59**:387–394.
87. Curtis C, Shah SP, Chin S-F, *et al.* The genomic and transcriptomic architecture of 2,000 breast tumours reveals novel subgroups. *Nature* 2012; **486**:346–352.
88. Comen EA, Robson M. Poly(ADP-Ribose) Polymerase Inhibitors in Triple-Negative Breast Cancer. *Cancer J* 2010; **16**:48–52.
89. Antoniou AC, Hardy R, Walker L, *et al.* Predicting the likelihood of carrying a BRCA1 or BRCA2 mutation: validation of BOADICEA, BRCAPRO, IBIS, Myriad and the Manchester scoring system using data from UK genetics clinics. *J Med Genet* 2008; **45**:425–431.
90. Couch FJ, Hart SN, Sharma P, *et al.* Inherited Mutations in 17 Breast Cancer Susceptibility Genes Among a Large Triple-Negative Breast Cancer Cohort Unselected for Family History of Breast Cancer. *J Clin Oncol* 2015; **33**:304–311.
91. Bauer M, Goldstein M, Christmann M, Becker H, Heylmann D, Kaina B. Human monocytes are severely impaired in base and DNA double-strand break repair that renders them vulnerable to oxidative stress. *Proc Natl Acad Sci U S A*. 2011; **108**:21105-10.
92. Krebs , Matthew G, et al. Phase I study of AZD6738, an inhibitor of ataxia telangiectasia Rad3 related (ATR), in combination with Olaparib or Durvalumab in patients with advanced solid cancer. AACR Annual Meeting 2018. Abstract No: CT026
93. Krebs , Matthew G, et al. Phase I clinical and translational evaluation of AZD6738 in combination with Durvalumab in patients (pts) with lung or head and neck carcinoma. *ESMO Annu Meet* 2018. Poster No. 413PD.

22 Appendices

Appendix 1: Synthetic Lethality

DNA Damage

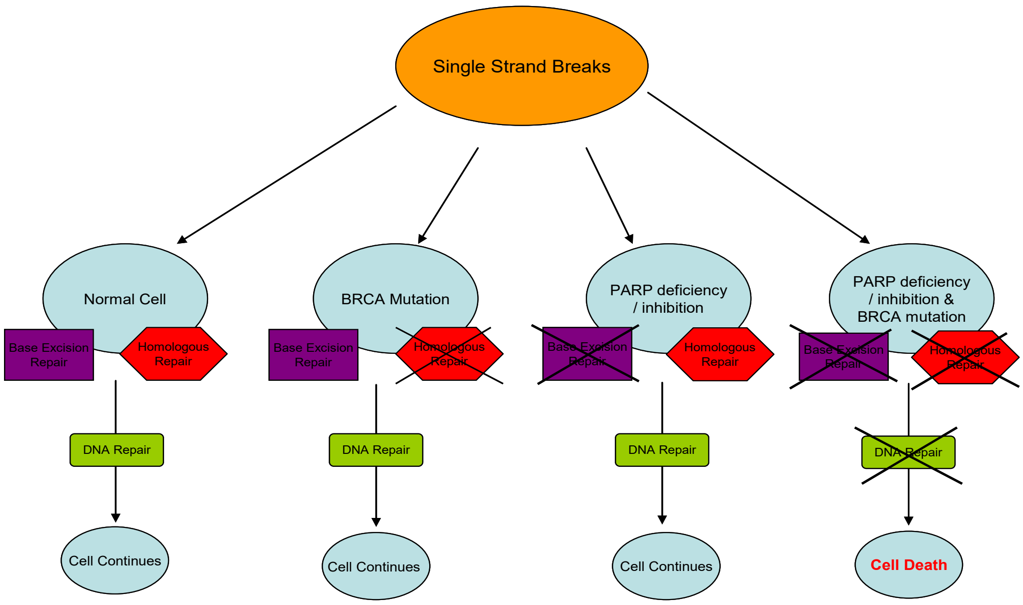

Fıgure adapted from Cancer Network Elızabeth A. Comen & Mark Robson 2010 [88].

Appendix 2: ECOG performance status

Grade	Description
0	Asymptomatic: normal activity
1	Symptomatic: fully ambulatory
2	Symptomatic: in bed < 50% of time
3	Symptomatic: in bed > 50% of time - not bedridden
4	100% bedridden
5	Death

Appendix 3: PARTNER -Timetable of events and investigations

Event	Prior to Registra-tion and Central testing	Prior to randomi-sation	After randomisation and Prior to start of treatment	Prior to each cycle of trial treatment i.e. Prior D1 for control arm and Research Arm 2 On Day-2 for Research Arm 1	Prior Paclitaxel D8 and D15 of cycle 1-4	Prior to cycle 2 or ASAP after	End of first regimen of chemo[a]/ prior second regimen	End of second regimen of chemo[b]/ surgery
Informed consent for central testing through Pre-Screening consent or Main consent	X							
BRCA test Results mandatory when only BRCA patients remain eligible		X						
Diagnosis slides available		X						
TIL score results mandatory		X						
TNBC phenotype Results mandatory prior randomisation for TNBC patients only		X						
Informed consent for trial (main consent)		X						
Serum Pregnancy test (if applicable) [g]		X				X		
Full blood count, Biochemical screen		X		X				X
GFR[c]			X			X		
ER, PgR,HER2 status		X						
Pre treatment assessment, ECOG status and vital signs.		X		X				X
Weight		X		X				
Height		X						
Staging as per local practice[d]		X						
Tumour radiological measurement		X					X	X
Medical History Questionnaire		X						
Optional QOL Questionnaire		X					X	X[h]
Neuropathy Questionnaire[e] +/- clinical assessment and scoring			X	X			X	X
Full blood count					X			
Mandatory Blood samples			X					X
Toxicity review of previous cycle including neuropathy				X				X
Surgery[f]								X
Diagnosis and Surgery tissue blocks with set of slides available								X
Optional Fresh tissue			X				X	X

a ***Cycles 1-4 = Paclitaxel/ Carboplatin+/ - Olaparib*** . Investigations to be carried out after the Paclitaxel/ Carboplatin+/- Olaparib regimen whether or not all 4 cycles of chemo given.

b ***Cycles 5-7 = Anthracyclines.*** Investigations to be carried out whether or not all 3 cycles of chemotherapy given.

c GFR to be calculated by serum creatinine prior chemotherapy and by EDTA GFR prior cycle 2. This is strongly recommended for BRCA patients, over and underweight patients and patients with previous renal diseases. Please ring the office for further details.

d Where applicable, see section 10.5.
e Neuropathy questionnaire before Cycle 1, after cycle 1-4, after cycle 7 and during follow-up
f Within 3 weeks of last day of final cycle of neoadjuvant chemotherapy.
g Repeat serum pregnancy test before cycle 2 for participants who have received fertility hormone injections and had false positive result at randomisation.
h Optional QOL questionnaire to be completed (i) after cycle 7, and (ii) after surgery and radiotherapy

Table 13: PARTNER Timetable for samples collection during first 12 months

	Prior to randomi -sation	Prior to cycle 1	Prior to cycle 2	Prior to cycle 5	After cycle 7 but prior to surgery	Immediately after surgery	2-4 weeks after surgery	6 months after surgery
BRCA test (5ml)	✓							
Diagnosis slides available	✓							
Mandatory Blood samples (18ml)		✓			✓ [b]			
Sequential Blood samples (up to 22.5ml) [a]		✓	✓	✓	✓ [c]	✓ [c]	✓ [c]	✓
Optional Fresh Tissue [d]		✓		✓	✓ [e]			
Blood for Anti-Mullerian Hormones [a] (9ml)		✓			✓ [c]			
Diagnosis and Surgery tissue blocks with set of slides available								✓
Saliva samples [f]		✓		✓	✓			✓
Urine Samples [f]		✓		✓	✓			✓
Stool samples [f]		✓		✓	✓			✓

[a] Only patients enrolled in the PARTNER Optional **Sequential Blood sub-study,** see section 18.4. Please refer to the current Lab Manual for collection time-points.

[b] Post-chemotherapy mandatory blood sample to be collected preferably prior to surgery, but in exceptional circumstances could be collected after surgery

[c] For patients enrolled in the optional **PartnerING pathway** these samples only need to be collected once.

[d] Only patients enrolled in the PARTNER Optional **Fresh Tissue samples collection,** see section 18.3. Please refer to the current Lab Manual for collection time-points.

[e] Fresh tumour tissue to be collected post-chemotherapy or during surgery.

[f] Only patients enrolled in the PARTNER Optional **Microbiome sub-study,** see section 18.5. Please refer to the current Lab Manual for collection time-points.

[redacted]

[redacted]

Appendix 5: Schedule for follow-up visits

It is allowed to contact patients by phone or email.

**FU visit #**	**Time from surgery**	**Approx. time from randomisation***	**Annual Follow-up Form to be completed**	**If Applicable** Relapse / Progression and Second Primary Form or **Details of Death Form to be completed**
1	6 months	13 months	X	✔
2	12 months (1 year)	19 months	✔	✔
3	18 months	25 months	X	✔
4	24 months (2 years)	31 months	✔	✔
5	36 months (3 years)	43 months	✔	✔
6	48 months (4 years)	55 months	✔	✔
7	60 months (5 years)	67 months	✔	✔
8	72 months (6 years)	79 months	✔	✔
9	84 months (7 years)	91 months	✔	✔
10	96 months (8 years)	103 months	✔	✔
11	108 months (9 years)	115 months	✔	✔
12	120 months (10 years)	127 months	✔	✔

***** Based on 28 weeks of neoadjuvant chemotherapy and allowance of up to 3 weeks for surgery following completion of chemotherapy.

**Event**	**Year 1**	**Year 2**	**Year 3**	**Year 4**	**Year 5**	**Year 6**	**Year 7**	**Year 8**	**Year 9**	**Year 10**	**Relapse**
Cancer assessment	✔	✔	✔	✔	✔	✔	✔	✔	✔	✔	
Neuropathy Questionnaire +/- clinical assessment and scoring[a]	✔	✔									
QOL Questionnaire	✔	✔									
Sequential Blood samples[b]	✔	✔	✔	✔	✔						✔
Blood for Anti-Mullerian Hormones[b]	✔										
Tumour blocks and slides											✔
Saliva sample[c]											✔
Urine sample[c]											✔
Stool sample[c]											✔

[a] At 3, 6, 12and 24 months of follow-up, see section 15.3.

[b] Only patients enrolled in the PARTNER **Optional Sequential Blood sub-study,** see section 18.4. Please refer to the current Lab Manual for collection time-points.

[c] Only patients enrolled in the PARTNER **Optional Microbiome sub-study,** see section 18.5. Please refer to the current Lab Manual for collection time-points.

Appendix 6: TNM Staging System for Breast Cancer

Used with the permission of the American Joint Committee on Cancer (AJCC), Chicago, Illinois. The original and primary source for this information is the AJCC Cancer Staging Manual, Sixth Edition (2002) published by Springer-Verlag New York. (For more information, visit www.cancerstaging.net.) Any citation or quotation of this material must be credited to the AJCC as its primary source. The inclusion of this information herein does not authorize any reuse or further distribution without the expressed, written permission of Springer-Verlag New York, Inc., on behalf of the AJCC.

Definitions of TNM

Primary Tumour (T)

TX Primary tumour cannot be assessed
T0 No evidence of primary tumour
Tis Carcinoma *in situ*: intraductal carcinoma, lobular carcinoma *in situ*, or Paget's disease of the nipple with no tumour
T1 Tumour ≤2 cm in greatest dimension
- T1mic Micro-invasion ≤0.1cm in greatest dimension
- T1a Tumour >0.1 but ≤0.5 cm or less in greatest dimension
- T1b Tumour >0.5 cm but ≤1 cm in greatest dimension
- T1c Tumour >1 cm but ≤2 cm in greatest dimension

T2 Tumour >2 cm but ≤5 cm in greatest dimension

T3 Tumour >5 cm in greatest dimension
T4 Tumour of any size with direct extension to (a) chest wall or (b) skin, only as described below.
- T4a Extension to chest wall, not including pectoralis muscle
- T4b Edema (including peau d'orange) or ulceration of the skin of the breast or satellite skin nodules confined to the same breast
- T4c Both (T4a and T4b)
- T4d Inflammatory carcinoma

Note: Paget's disease associated with a tumour is classified according to the size of the tumour.

Regional Lymph Nodes (N)

NX Regional lymph nodes cannot be assessed (for example, previously removed)
N0 No regional lymph node metastasis
N1 Metastasis to movable ipsilateral axillary lymph node(s)
N2 Metastasis to ipsilateral axillary lymph node(s) fixed or matted, or in clinically apparent ipsilateral internal mammary nodes in the absence of clinically evident* axillary lymph node metastasis
- N2a Metastasis in ipsilateral axillary lymph nodes fixed (or matted) to one another or to other structures
- N2b Metastasis only in clinically apparent* ipsilateral internal mammary nodes *and* in the absence of clinically evident axillary lymph node metastasis

N3 Metastasis to ipsilateral infraclavicular lymph node(s) with or without axillary lymph node involvement, or in clinically apparent* ipsilateral internal mammary lymph node(s) and in the presence of clinically evident axillary or internal mammary lymph node involvement; or metastases in ipsilateral supraclavicular lymph node(s) with or without axillary or internal mammary lymph node involvement
- N3a Metastasis in ipsilateral infraclavicular lymph node(s)
- N3b Metastasis in ipsilateral internal mammary lymph node(s) and axillary lymph node(s)
- N3c Metastasis in ipsilateral supraclavicular lymph node(s)

**** Clinically apparent is defined as detected by imaging studies (excluding lymphoscintigraphy) or by clinical examination or grossly visible pathologically.***

Distant Metastasis (M)

MX Presence of distant metastasis cannot be assessed
M0 No distant metástasis
M1 Distant metastasis

Stage grouping and eligibility for PARTNER

STAGE GROUPING	T	N	M
Stage 0	*Tis*	*N0*	*M0*
Stage I	T1	N0	M0
Stage IIA	T0	N1*	M0
	T1	N1**	M0
	T2	N0	M0
Stage IIB	T2	N1	M0
	T3	N0	M0
Stage IIIA	T0	N2*	M0
	T1	N2**	M0
	T2	N2	M0
	T3	N1	M0
	T3	N2	M0
Stage IIIB	T4	N0	M0
	T4	N1	M0
	T4	N2	M0
Stage IIIC	Any T	N3	M0
Stage IV	Any T	Any N	M1

White italics indicate ineligible for PARTNER trial.

* / ** TO, T1mic, T1a and T1b tumours are only eligible in presence of axillary node >10mm

***TO N1,N2 tumours must be histopathologically confirmed by LN biopsy (trucut or whole LN)**

Appendix 7: Criteria for BRCA1 and BRCA2 genes mutation testing within PARTNER (UK patients only)

NHS Genomic Test Directory Criteria for BRCA1 and BRCA2 genes mutation

Referrals are only accepted from one of the following:
Consultant Clinical Geneticist/Registered Genetic Counsellor
Consultant Oncologist
Consultant Breast Surgeon

Patients affected with breast cancer can be tested within the NHS if they meet ONE of the following criteria:

- Breast cancer (age <30 years)
- Bilateral breast cancer (age <550 years)
- Triple negative breast cancer (age <60 years)
- Male breast cancer (any age)
- Breast cancer (age <45 years) and a first degree relative with breast cancer (age <45 years)
- High grade non-mucinous epithelial ovarian cancer at any age
- Pathology adjusted Manchester score greater than or equal to 15 [89] or BOADICEA score greater than or equal to 10%
- Ashkenazi Jewish ancestry and breast cancer at any age

To ensure that BRCA testing results are available for randomisation, we offer to test the patients listed above in the fast track BRCA testing platform (sample testing may be outsourced if capacity issues arise). For these patients, the cost of the test will be covered by the referral centre as part of the standard NHS testing as stated in the clinical trial agreement.

Additional PARTNER Criteria for BRCA1 and BRCA2 genes mutation
Patients not complying with the criteria above but presenting with one of the criteria below can be tested within the trial by the fast track BRCA testing platform (sample testing may be outsourced if capacity issues arise). The cost of the test will be covered by the trial as stated in the clinical trial agreement.

- ER positive breast cancer patients <60 with a strong family history.
- Strong family history of breast, ovarian, pancreatic and/or high grade/metastatic prostate cancer.

Appendix 8: Formulae, Calculations, and Abbreviations

Cockcroft-Gault formula for calculating Creatinine Clearance:

The estimated GFR is given by:

Males: $\frac{1.25 \times (140 - \text{age}) \times \text{weight (kg)}}{\text{serum creatinine (umol/l)}}$

Females: $\frac{1.05 \times (140 - \text{age}) \times \text{weight (kg)}}{\text{serum creatinine (umol/l)}}$

Dubois & Dubois formula for calculation Body Surface Area:

Body Surface Area (m) = 0.007184 × (patient height (cm))$^{0.725}$ × (patient weight (kg))$^{0.425}$

Appendix 9: Contraception Guidelines

Patients of child bearing potential and their partners, who are sexually active, must agree to the use of two highly effective forms of contraception throughout their participation in the trial and for at least 6 months after last dose of trial drug(s).

- Condom with spermicide

and one of the following

- Oral contraceptive or hormonal therapy (e.g. hormone implants)
- Placement of an intra-uterine device

Acceptable non-hormonal birth control methods include:

- True abstinence: Where this is in line with the preferred and usual lifestyle of the patient.
- Vasectomised sexual partner plus male condom. With patient assurance that partner received post-vasectomy confirmation of azoospermia
- Tubal occlusion plus male condom with spermicide
- Intrauterine Device (IUD) plus male condom+spermicide. Provided coils are copper-banded

Acceptable hormonal methods:

- Etonogestrel implants (eg, Implanon, Norplan)+male condom with spermicide
- Normal and low dose combined oral pills+male condom with spermicide
- Norelgestromin/ethinyl estradiol (EE) transdermal system+male condom with spermicide
- Intravaginal device+male condom with spermicide (eg, EE and etonogestrel)
- Cerazette (desogestrel)+male condom with spermicide. Cerazette is currently the only highly efficacious progesterone based pill.

[redacted]

[redacted]

[redacted]

[redacted]

Appendix 10: Possible exclusion exceptions in cases of severe pre-existing sensory or motor disability

Patients presenting with significant pre-existing disability would require a baseline clinical assessment and documentation of type and extent of disability. If after assessment it is found that the grade of sensory or motor neuropathy is so significant that no further damage could be caused by the use of the trial medication then the patient should be discussed with the PARTNER office and a decision will be made regarding eligibility.

Patients presenting with confirmed and definitive paralysis of a limb but no neuropathy symptoms are eligible. It is strongly recommended that these patients have an EDTA GFR test done, as the Cockcroft-Gault calculation may overestimate their GFR due to atrophied muscle giving an inappropriately low serum creatinine.

[redacted]

- [redacted]
- [redacted]
- [redacted]
- [redacted]
- [redacted]
- [redacted]
- [redacted]
- [redacted]
- [redacted]
- [redacted]
- [redacted]
- [redacted]
- [redacted]
- [redacted]
- [redacted]
- [redacted]
- [redacted]
- [redacted]
- [redacted]
- [redacted]
- [redacted]
- [redacted]
- [redacted]
- [redacted]

[redacted]

Appendix 12 Guidelines regarding Olaparib [redacted] potential interactions with concomitant medications

Restrictions regarding drugs affection CYP3A metabolism

There are currently no data confirming that there is a pharmacokinetic (PK) interaction between these agents and Olaparib or [redacted]; a potential interaction is considered on the basis of preclinical and in vitro data only. [redacted] is predominantly eliminated via CYP3A metabolism, therefore CYP3A inhibitors or inducers may increase or decrease exposure to [redacted], respectively. Potent or moderate inhibitors or inducers of CYP3A should not be combined with [redacted] or Olaparib. Weak inhibitors or inducers of CYP3A4 are permitted but caution should be exercised and patients monitored closely for possible drug interactions. Appropriate medical judgment is required. Please contact PARTNER Office with any queries you have on this issue. Please refer to full prescribing information for all drugs prior to co-administration with [redacted]

Table 15. Drugs Known to be Inhibitors and inducers of CYP3A

Potent CYP3A inhibitors	Moderate CYP3A Inhibitors	Weak CYP3A Inhibitors	Potent CYP3A inducers
boceprevir	amprenavir	alprazolam	avasimibe
clarithromycin	**aprepitant**	amlodipine	carbamazepine
conivaptan	atazanavir	atorvastatin	enzalutamide
elvitegravir/ RIT	casopitant	azithromycin	mitotane
fluconazole	cimetidine	berberineb	phenobarbital
grapefruit juice[a]	ciprofloxacin	bicalutamide	phenytoin
indinavir	crizotinib	chlorzoxazone	rifabutin
itraconazole	cyclosporine	cilostazol	rifampin
ketoconazole	darunavir	clotrimazole	St John's Wort
lopinavir/ RIT	diltiazem	cranberry juice	
mibefradil	dronedarone	delavirdine	
nefazodone	erythromycin	fluvoxamine	
nelfinavir	grapefruit juicec	fosaprepitant (IV)	
posaconazole	imatinib	ginkgob	
ritonavir	schisandra	goldensealb	
saquinavir	sphenanthera	isoniazid	
telaprevir	tofisopam	ivacaftor	
telithromycin	verapamil	lacidipine	
tipranavir/RIT		linagliptin	
troleandomycin		nilotinib	
voriconazole		oral contraceptives	
		pazopanib	
		peppermint oil	
		propiverine	
		ranitidine	
		ranolazine	
		resveratrol	
		roxithromycin	
		Seville orange juicec	
		sitaxentan	
		tabimorelin	
		tacrolimus	
		ticagrelor	
		tolvaptan	

List created using the University of Washington Drug-Drug Interaction Database January 2013.
RIT = Ritonivir. Ritonavir has dual effects of simultaneous CYP3A inhibition and induction, and the net pharmacokinetic outcome during chronic ritonavir therapy is inhibition of CYP3A activity
a. Double-strength grapefruit juice. Consumption of grapefruit and Seville oranges (and other products containing these fruits eg, grapefruit juice or marmalade) are prohibited for all patients during trial treatment.

Drugs known to be inhibitors or inducers of Pgp and/or BCRP, undertake appropriate monitoring if coadministration is necessary

[redacted] is also a substrate of Pgp and BCRP. Co-administration of Pgp inhibitors/inducers or BCRP inhibitors/inducers may affect exposure to [redacted] therefore it is recommended that these are not co-administered with [redacted].

These lists are not intended to be exhaustive, and similar restrictions will apply to other agents that are known to modulate Pgp activity or BCRP activity. Please contact PARTNER Office with any queries you have on this issue. Please refer to full prescribing information for all drugs prior to co-administration with [redacted].

Table 16. Drugs known to be inhibitors or inducers of Pgp

Drugs Known to be Inhibitors of Pgpa			Drugs Known to be Inducers of Pgpb
Amiodarone<br>azithromycin<br>captopril<br>carvedilol<br>clarithromycin<br>conivaptan<br>cremophor<br>curcumin<br>diltiazem<br>dronedarone<br>elacridar<br>erythromycin<br>felodipine<br>fluvoxamine<br>ginkgo<br>indinavir<br>itraconazole<br>ketoconazole	lapatinib<br>lopinavir and ritonivir<br>mibefradil<br>milk thistle<br>mirabegron<br>nelfinavir<br>nifedipine<br>nitrendipine<br>paroxetine<br>quercetin<br>quinidine<br>ranolazine<br>rifampin<br>ritonavir<br>saquinavir/ritonavir	schisandra chinensis extract<br>St Johns Wort<br>talinolol<br>telaprevir<br>telmisartan<br>ticagrelor<br>tipranavir/ritonavir<br>tolvaptan<br>valspodar (PSC 833)<br>verapamil	avasimibe<br>carbamazepine<br>efavirenz<br>genistein<br>phenytoin<br>rifampin<br>St Johns Wort

a. Inhibitors listed for P-gp are those that showed >25% increase in exposure to a P-gp substrate (e.g. digoxin).
b. Inducers listed for Pgp are those that showed >20 % decrease in exposure to a P-gp substrate (e.g. digoxin)

Table 17.Drugs known to be inhibitors or inducers of BCRP

Drugs Known to be Inhibitors of BCRP		Drugs Known to be inducers of BCRP
Afatinib<br>Aripiprazole<br>Curcumin<br>Cyclosporine<br>Elacridar<br>Erlotinib<br>Fluvastatin<br>Fumitremorgin<br>Gefitinib<br>Ivermectin<br>Lapatinib<br>Nilotinib<br>Novobiocin<br>Pantoprazole<br>Pitavastatin<br>Ponatinib<br>Quercetin<br>Quizartinib<br>Rabeprazole	Regorafenib<br>Rilpivirine<br>Sulfasalazine<br>Sunitinib<br>Tacrolimus<br>Teriflunomide<br>Trametinib<br>Trifluoperazine<br>Vismodegib<br>eltrombopag<br>Atazanavir<br>Lopinavir<br>Ritonavir<br>Tipranavir<br>Omeprazole<br>Estrone<br>17b-estradiol<br>Imatinib mesylate	Please check individual drugs on a case by case basis

List created using http://dmd.aspetjournals.org/content/dmd/43/4/490.full.pdf
Note: Although BCRP is involved in a number of clinically relevant DDIs, none of the cited inhibitors above is truly specific for this transporter

Drugs known to be substrates of CYP3A4 and/or CYP2B6, undertake appropriate monitoring if coadministration is necessary

[redacted] is a potential inducer of CYP3A4 and CYP2B6. Therefore caution should be applied with co-administration of drugs that are either completely metabolised by CYP3A4 and/or CYP2B6, or that are substrates of CYP3A4 and/or CYP2B6 and also have a narrow therapeutic index. Investigators should be aware that the exposure of other drugs metabolised by CYP3A4 and/or CYP2B6 may be reduced.

Table 18. Drugs known to be substrates of CYP3A4 and/or CYP2B6

Drugs known to be metabolised by CYP3A4 and have a narrow therapeutic index	Drugs known to be metabolised by CYP2B6 and have a narrow therapeutic index
Alfentanil	Cyclophosphoamide
Cyclosporine	Ifosfamide
Dihydroergotamine	Efavirenz
Ergotamine	Bupropion
Fentanyl	Propofol
Pimozide	Thiotepa
Quinidine	Sorafenib
Sirolimus	alfentanil
Tacrolimus	ketamine
Astemizole	methadone
Cisapride	methoxetamine
Terfenadine	nevirapine
	selegiline
	sertraline
	sorafenib
	tamoxifen
	valproic acid

From Flockhart DA (2007). "Drug Interactions: Cytochrome P450 Drug Interaction Table". Indiana University School of Medicine

Drugs known to be substrates of OATP1B1, BCRP, OTAP1B3, MATE1K and MATE2K. undertake appropriate monitoring if coadministration is necessary.

[redacted] is also an inhibitor of OATP1B1, BCRP, OTAP1B3, MATE1K and MATE2K. Caution should be applied with coadministration of substrates of OATP1B1 and/or BCRP, OTAP1B3, MATE1K and MATE2K as AZD6738 may increase their exposure.

These lists are not intended to be exhaustive and appropriate medical judgment is required. Please contact PARTNER Office with any queries you have on this issue. Please refer to full prescribing information for all drugs prior to co-administration with AZD6738.

Table 19. Drugs known to be substrates of OATP1B1, BCRP, OTAP1B3, MATE1K and MATE2K.

Drugs known to be substrates of OATP1B1		Drugs known to be substrates of BCRP	
Atorvastatin	Methotrexate	Anthracyclines	Mitoxantrone
Fluvastatin	Rifampin	Daunorubicin	nucleoside analogs
Lovastatin	Bosentan	Doxorubicin	prazosin
Pitavastatin	Glyburide	Topotecan	pantoprazole
Pravastatin	Repaglinide	SN-38	topotecan
Rosuvastatin	Valsartan	Irinotecan	rosuvastatin and other statins
Simvastatin	Olmesartan	Methotrexate	teriflunomide
Ezemibe	Atrasentan	Imatinib	chlorothiazide
Simvastatin		Irinotecan	

Drugs known to be substrates of OTAP1B3		Drugs known to be substrates of MATE1K	
Statins<br>Fexofenadine<br>Valsartan<br>Telmisartan<br>Enalapril	Erythromycin<br>Mycophenolic acid<br>Tacrolimus,<br>(phalloidin, microcystin-LR)	Cimetidine<br>Metformin<br>Cephalexin<br>Acyclovir<br>Ganciclovir<br>Cephalexin<br>Cephradine	Fexofenadine<br>Oxaliplatin<br>Procainamide<br>Topotecan<br>Pramipexole<br>Atenolol (paraquat)

Drugs known to be substrates of MATE2K	
Metformin<br>Cimetidine<br>Procainamide<br>Guanidine<br>Topotecan<br>Acyclovir	Ganciclovir<br>Quinidine<br>lamivudine<br>Oxaliplatin<br>Varenicline

List created using https://www.solvobiotech.com/transporters/

[redacted]

[redacted]

[redacted]

[redacted]

[redacted]

[redacted]

[redacted]

[redacted]

[redacted]

[redacted]

[redacted]

[redacted]

[redacted]

[redacted]

[redacted]	[redacted]
[redacted]	[redacted]
[redacted]	[redacted]
[redacted]	[redacted]
[redacted]	[redacted]

[redacted]

[redacted]

[redacted]

[redacted]

[redacted]

[redacted]	[redacted]
[redacted]	[redacted]

[redacted]	[redacted]
[redacted]	[redacted]
[redacted]	[redacted]
[redacted]	[redacted]
[redacted]	[redacted]

[redacted]

[redacted]

[redacted]

[redacted]

[redacted]

[redacted]	[redacted]
[redacted]	[redacted]
[redacted]	[redacted]<br>[redacted]

[redacted]

[redacted]

- [redacted]
- [redacted]

[redacted]

- [redacted]
- [redacted]

[redacted]

Appendix 14: Prolonged haematological toxicities while on study treatment.

If a patient develops prolonged haematological toxicities (due to either AZD6738 or Olaparib) such as:

- ≥2 week interruption/delay in study treatment due to CTC grade 3 or worse anaemia and/or development of blood transfusion dependence.
- ≥2 week interruption/delay in study treatment due to CTC grade 3 or worse neutropenia (ANC < 1 x 109/L).
- ≥2 week interruption/delay in study treatment due to CTC grade 3 or worse thrombocytopenia and/or development of platelet transfusion dependence (Platelets < 50 x 109/L).

Check weekly differential blood counts including reticulocytes and peripheral blood smear. If any blood parameters remain clinically abnormal after 4 weeks of dose interruption, the patient should be referred to haematologist for further investigations.

Bone marrow analysis and/or blood cytogenetic analysis should be considered at this stage according to standard haematological practice.

Development of a confirmed MDS or other clonal blood disorder should be reported as an SAE and full reports must be provided by the Investigator to the PARTNER office.

The effect, if any of [redacted] *on the bone marrow in terms of prolonged suppression is unknown.*

Appendix 15: Actions Required in Cases of Increases in Liver Biochemistry and Evaluation of Hy's Law

This Appendix describes the process to be followed in order to identify and appropriately report cases of Hy's Law. It is not intended to be a comprehensive guide to the management of elevated liver biochemistries.

During the course of the study the Investigator will remain vigilant for increases in liver biochemistry. The Investigator is responsible for determining whether a patient meets potential Hy's Law (PHL) criteria at any point during the study.

HL criteria are met if there is no alternative explanation for the elevations in liver biochemistry other than Drug Induced Liver Injury (DILI) caused by the Investigational Medicinal Product (IMP). The Investigator is responsible for recording data pertaining to PHL/HL cases and for reporting Adverse Events (AE) and Serious Adverse Events (SAE) according to the outcome of the review and assessment in line with standard safety reporting processes.

Table 22. Evaluation of Hy's Law- Definitions:

Potential Hy's Law (PHL):	*Aspartate Aminotransferase (AST) or Alanine Aminotransferase (ALT) ≥ 3x Upper Limit of Normal (ULN) together with Total Bilirubin (TBL) ≥ 2xULN at any point during the study following the start of study medication irrespective of an increase in Alkaline Phosphatase (ALP).*
Hy's Law (HL):	*AST or ALT ≥ 3x ULN together with TBL ≥ 2xULN, where no other reason, other than the IMP, can be found to explain the combination of increases, eg, elevated ALP indicating cholestasis, viral hepatitis, another drug.*

For PHL and HL the elevation in transaminases must precede or be coincident with (i.e. on the same day) the elevation in TBL, but there is no specified timeframe within which the elevations in transaminases and TBL must occur.

If the patient does meet PHL criteria the Investigator will:

- Notify PARTNER Office.
- The PARTNER team will contact the investigator to provide additional guidance required, discuss and agree an approach for the study patients' follow-up and the continuous review of data.

Subsequent to this contact the Investigator will:

- Monitor the patient until liver biochemistry parameters and appropriate clinical symptoms and signs return to normal or baseline levels, or as long as medically indicated
- Investigate the aetiology of the event and perform diagnostic investigations as discussed with the Study Physician
- Complete the three Liver CRF Modules as information becomes available
- If at any time (in consultation with the PARTNER office) the PHL case meets serious criteria, report it as an SAE using standard reporting procedures

No later than 3 weeks after the biochemistry abnormality was initially detected, the PARTNER Office will contact the Investigator in order to review available

data and agree on whether there is an alternative explanation for meeting PHL criteria other than DILI caused by the IMP.

If there is an agreed alternative explanation for the ALT or AST and TBL elevations, a determination of whether the alternative explanation is an AE will be made and subsequently whether the AE meets the criteria for a SAE.

- If the alternative explanation is not an AE, record the alternative explanation on the appropriate CRF.
- If the alternative explanation is an AE/SAE, record the AE /SAE in the CRF accordingly.

If it is agreed that there is no explanation that would explain the ALT or AST and TBL elevations other than the IMP:

- Report an SAE (report term 'Hy's Law') according to standard processes.
  - The 'Medically Important' serious criterion should be used if no other serious criteria apply.
  - As there is no alternative explanation for the HL case, a causality assessment of 'related' should be assigned.

If, there is an unavoidable delay, of over 3 weeks, in obtaining the information necessary to assess whether or not the case meets the criteria for HL, then it is assumed that there is no alternative explanation until such time as an informed decision can be made:

- Report an SAE (report term 'Potential Hy's Law') applying serious criteria and causality assessment as per above.
- Continue follow-up and review according to agreed plan. Once the necessary supplementary information is obtained, repeat the review and assessment to determine whether HL criteria are met. Update the SAE report according to the outcome of the review.

FDA Guidance for Industry (issued July 2009) 'Drug-induced liver injury: Premarketing clinical evaluation:
http://www.fda.gov/downloads/Drugs/GuidanceComplianceRegulatoryInformation/Guidances/UCM174090.pdf

[redacted]

[redacted]

[redacted]

[redacted]
[redacted]

[redacted]

[redacted]

[redacted]

[redacted]

[redacted]

[redacted]

[redacted]

[redacted]

[redacted]

[redacted]

[redacted]

[redacted]

[redacted]

[redacted]

[redacted]

[redacted]

[redacted]

[redacted]

[redacted]

[redacted]

[redacted]

[redacted]

[redacted]

[redacted]

[redacted]

[redacted]

[redacted]

[redacted]
